# Genus *Cistus:* a model for exploring labdane-type diterpenes' biosynthesis and a natural source of high value products with biological, aromatic, and pharmacological properties

**DOI:** 10.3389/fchem.2014.00035

**Published:** 2014-06-11

**Authors:** Dimitra Papaefthimiou, Antigoni Papanikolaou, Vasiliki Falara, Stella Givanoudi, Stefanos Kostas, Angelos K. Kanellis

**Affiliations:** ^1^Group of Biotechnology of Pharmaceutical Plants, Laboratory of Pharmacognosy, Department of Pharmaceutical Sciences, Aristotle University of ThessalonikiThessaloniki, Greece; ^2^Department of Chemical Engineering, Delaware Biotechnology Institute, University of DelawareNewark, DE, USA; ^3^Department of Floriculture, School of Agriculture, Aristotle University of ThessalonikiThessaloniki, Greece

**Keywords:** *Cistus*, biosynthesis, labdane-type diterpenes, phenylpropanoids, biological action, genomic approaches

## Abstract

The family Cistaceae (Angiosperm, Malvales) consists of 8 genera and 180 species, with 5 genera native to the Mediterranean area (*Cistus, Fumara, Halimium, Helianthemum*, and *Tuberaria*). Traditionally, a number of *Cistus* species have been used in Mediterranean folk medicine as herbal tea infusions for healing digestive problems and colds, as extracts for the treatment of diseases, and as fragrances. The resin, ladano, secreted by the glandular trichomes of certain *Cistus* species contains a number of phytochemicals with antioxidant, antibacterial, antifungal, and anticancer properties. Furthermore, total leaf aqueous extracts possess anti-influenza virus activity. All these properties have been attributed to phytochemicals such as terpenoids, including diterpenes, labdane-type diterpenes and clerodanes, phenylpropanoids, including flavonoids and ellagitannins, several groups of alkaloids and other types of secondary metabolites. In the past 20 years, research on *Cistus* involved chemical, biological and phylogenetic analyses but recent investigations have involved genomic and molecular approaches. Our lab is exploring the biosynthetic machinery that generates terpenoids and phenylpropanoids, with a goal to harness their numerous properties that have applications in the pharmaceutical, chemical and aromatic industries. This review focuses on the systematics, botanical characteristics, geographic distribution, chemical analyses, biological function and biosynthesis of major compounds, as well as genomic analyses and biotechnological approaches of the main *Cistus* species found in the Mediterranean basin, namely *C. albidus, C. creticus, C. crispus, C. parviflorus, C. monspeliensis, C. populifolius, C. salviifolius, C. ladanifer, C. laurifolius*, and *C. clusii*.

## Introduction

*Cistus* L. (from the Greek word kistos-κíστoς) or rock rose, is a genus of dicotyledonous perennial herbaceous plants that have hard leaves and grow in open areas of stony and infertile soils. They are indigenous to the Mediterranean region and are known for their durability. Even after natural regional forest fires, these plants are capable to grow due to their increased seed germinability after exposure of the seeds to high temperatures (Thanos et al., 1992). In some species seasonal dimorphism is observed, enabling the plants' adaptation to drought conditions, which induces leaves to decrease in size and grow more hair (Aronne and Micco, 2001). The characteristic feature of the genus is a combination of diverse hair types on the leaf, stem, and calyx including non-glandular trichomes. The tufted and stellate as well as the elongate glandular trichomes produce and secrete a resin. In some species (e.g., *C. creticus* subsp. *creticus*), this resin is rich in biologically-active and pharmacologically-interesting metabolites, such as flavonoid aglycones, glycosides, and terpenoids including labdane-type diterpenes. Since the original description of the genus in 1753 by Linnaeus, a number of species and subspecies have been categorized within *Cistus*. After several taxonomic re-evaluations, about 21 species of *Cistus* are now recognized spreading within the white and pink-flowered lineages (Guzmán and Vargas, 2005). *Cistus* species are distributed both in the eastern and western Mediterranean, where the highest diversity is observed, and are also widespread in the Balearic and Canarian islands (Guzmán and Vargas, 2005, 2010). Several of them have been employed in Mediterranean folk medicine as herbal tea infusions for healing digestive problems and colds, as extracts for the treatment of diseases, and as fragnances. The resin, ladano, produced by *C. creticus* in Crete, Greece and Cyprus, and *C. ladanifer* in Spain, is exported to a number of Arabic countries where it is used as insence.

In the past 20 years, research on *Cistus* was of chemical, biological, and phylogenetic nature. Added to this list are the recent genomic and molecular studies. Metabolomic analyses using chromatographic and spectroscopic tools allowed the identification of several chemical groups with distinct biological activities. Among the most important compounds are terpenoids, including diterpenes, labdane-type diterpenes and clerodanes, phenylpropanoids, including flavonoids and ellagitannins, several groups of alkaloids and some other secondary metabolites.

A plentiful of biological functions have been attributed to the resin produced by these species. Pharmacological studies on *Cistus* extracts have demonstrated their action as antioxidants (Attaguile et al., 2000; Hernández et al., 2004; Sadhu et al., 2006; Sarić et al., 2009; Amensour et al., 2010; Barrajón-Catalán et al., 2010; Akkol et al., 2012; Riehle et al., 2013; Zidane et al., 2013), antibacterial and antifungal (Chinou et al., 1994; Bouamama et al., 1999; Barrajón-Catalán et al., 2010; Barros et al., 2013), antiviral (Droebner et al., 2007; Ehrhardt et al., 2007), anti-cancer (Chinou et al., 1994; Demetzos et al., 1994a, 2001; Dimas et al., 1998; Angelopoulou et al., 2001a; Dimas et al., 2006; Hatziantoniou et al., 2006; Barrajón-Catalán et al., 2010; Skorić et al., 2012), and other functions discussed later in the review.

The ability of several *Cistus* species to produce high amounts of natural metabolites makes them attractive models for the elucidation of their biosynthetic pathways. The pathway leading to the production of terpenes, especially labdane-type diterpenes, has been investigated in *C. creticus* subsp. *creticus* and several genes have been characterized (Falara et al., 2008, 2010; Pateraki and Kanellis, 2008, 2010). Among these are the germacrene B synthase (*CcGrB*), (Falara et al., 2008), the 3-hydroxy-3-methylglutaryl-coenzyme A reductase (*CcHMGR*), DXP reductoisomerase (*CcDXR*) and 1-deoxy-D-xylulose-5-phosphate synthase (*CcDXS*) (Pateraki and Kanellis, 2010), two active homologs of geranyl-geranyl diphosphate synthase (*CcGGDPS1, CcGGDPS2*) (Pateraki and Kanellis, 2008), and copal-8-ol diphosphate diterpene synthase (Falara et al., 2010).

In this review we focus on the major representatives of *Cistus* species: *C. albidus, C. creticus, C. crispus, C. parviflorus, C. monspeliensis, C. populifolius, C. salviifolius, C. ladanifer, C. laurifolius*, and *C. clusii*, which are commonly found in the Mediterranean basin. Due to their plethora of uses and potential valuable therapeutical activities they have been extensively studied.

## Systematics of *Cistus* species

The family Cistaceae (Angiosperm, Malvales) consists of 8 genera (Arrington and Kubitzki, 2003) and 180 species, with 5 genera native to the Mediterranean area (*Cistus, Fumara, Halimium, Helianthemum*, and *Tuberaria*). The taxonomic separation of the genus is based on phenotypic observations, including morphological characters like shape, nerve number, color and trichomes of leaves and stems, and reproductive characteristics such as petal and sepal number, shape and color of flowers, number of fruit valves and style size. The phenotype-based genus taxonomy was confirmed recently using plant chemotype and molecular approaches.

Taxonomic classification of *Cistus* was formed prior to 1800 (Linnaeus, 1753), but the first integrated separation was implemented in 1824 by Dunal (1824), who described 28 species divided in 2 sections, *Erythrocistus* and *Ledonia*. Shortly thereafter, Sweet (1830) described 33 species, also divided into *Erythrocistus* and *Ledonia*, where 3 additional species in section *Erythrocistus* and 7 species in section *Ledonia* were included. Spach (1836) separated them in 5 genera, named *Ladanium, Rhodocistus, Stephanocarpus, Ledonia* and *Cistus*, further divided into sections *Rhodopsis, Eucistus*, and *Ledonella*. The plant species divided in subgenera *Erythrocistus* and *Ledonia* were further separated into 7 sections: *Macrostylia, Brachystylia*, and *Astylia* in subgenus *Erythrocistus* and *Stephanocarpus, Ledonia, Ladanium*, and *Halimioides* in subgenus *Leucocistus* (Willkomm, 1856). Grosser (1903) described 3 groups distributed into 16 species in 7 sections: Group A contained *Rhodocistus, Eucistus*, and *Ledonella* while Groups B and C, respectively, made up of *Stephanocarpus* and *Ledonia*, and *Ladanium* and *Halimioides*. Dansereau (1939) classified the species in subgenera *Erythrocistus* and *Ledonia*, like Willkomm, and then separated them in 8 sections, with naming *Macrostylia, Erythrocistus*, and *Ledonella* for sections of subgenus *Erythrocistus*, and *Stephanocarpoidea, Stephanocarpus, Ledonia, Ladanium*, and *Halimioides* for sections of subgenus *Leucocistus*.

More recently, Demoly and Montserrat (1993) described the distribution of 12 species of genus *Cistus* that grow in Iberia. In this approach, 3 subgenera were classified: I. subgenus *Cistus*, containing *C. albidus, C. creticus, C. crispus*, and *C. heterophyllus*; II. subgenus *Leucocistus*, containing *Ledonia* with species *C. monspeliensis, C. salviifolius, C. psilosepalus*, and *C. populifolius*, and section *Ladanium* with *C. ladanifer* and *C. laurifolius*; and III. subgenus *Halimioides* containing *C. clusii* and *C. libanotis*. From this study, it became apparent that most *Cistus* species grow in western Mediterranean. The same conclusion was reached for the species distribution (Table S1), where the main 10 *Cistus* species, discussed in this review, are distributed in 28 areas. Specifically, numerous species grow in Spain followed by Morocco, Italy, Portugal, Algeria, and France. Conversely, in the eastern Mediterranean the number of species is low with the most widespread species being *C. creticus, C. palviflorus*, and *C. salvifolius*.

**Figure 1:**
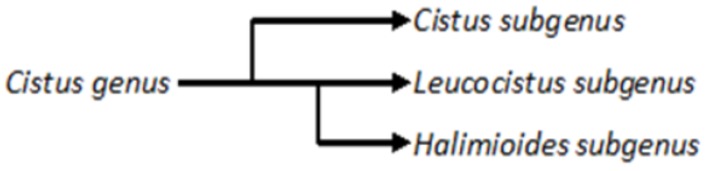
**Classification of three subgenus of Cistus genus based on analysis of *trnF, matK* and ITS sequences (Guzmán and Vargas, 2005) and plastid *rbcL* and *trnL-trnF* sequences (Guzmán and Vargas, 2009)**.

A recent classification of *Cistaceae* is based on combined nuclear (ncp*GS*, ITS) and plastidic (*trnL-trnF, trnK-matK, trnS-trnG, rbcL*) DNA sequence comparisons, which divided *Cistus* into 3 subgenera (similar to Demoly and Montserrat, 1993): the purple flowered subgenus *Cistus* and the white flowered subgenera *Leucocistus* and *Halimioides* (Figure 1) (Guzmán and Vargas, 2005; Guzmán et al., 2009). Interestingly, *C. palviflorus* appeared most closely related to subgenus *Leucocistus* (white flowers), although it possesses light purple flowers. Similar observations led in the past to the creation of a separate section for *C. palviflorus*, namely Ledonella. In another work, the evolution of family Cistaceae was studied by the phylogenetic analysis of plastid *rbcL* and *trnL-trnF* sequences (Guzmán and Vargas, 2009). This study confirmed the clear separation of the genus into 2 groups, with purple (excluding *C. palviflorus*) and white flowers and certified the family Cistaceae as monophyletic, sisterly to families Dipterocarpaceae and Sarcolaenaceae (Guzmán et al., 2009). A similar classification was achieved by analyzing polyphenolic composition of aerial parts of the most common species, which separated *Cistus* subgenus from the two other subgenera by its higher flavonoid content (Barrajón-Catalán et al., 2011).

Another phylogenetic study confirmed the chemical and genetic (*ISSR*—PCR amplification) differentiation between the *C. creticus* subspecies *eriocephalus* and *corsicus* (Paolini et al., 2009). The affinity and distance estimation between individual plants of *C. creticus* L. in Corsica and Sardinia, as inferred by the *trn-F and RPL32-TRNL* sequences of *cpDNA*, showed that the plants were divided into 4 groups having evident correlation with the region (Falchi et al., 2009). In another taxonomic approach the composition of essential oils of *C. salviifolius* in 15 Cretan (Greece) populations was studied, dividing the plants into 3 groups, most of them belonging to the group with high camphor production (Demetzos et al., 2002b). Also, a chemometric interpopulation study of *C. creticus* in the East and West parts of Crete showed the existence of a high variability within the essential oils. The plants were geographically divided into three groups, two in the West and one in the Eastern part of Crete (Demetzos et al., 2002a).

## Botanical characteristics

### Genus *Cistus L*

*Cistus* plants are small, woody shrubs with a straight stem that has opposite rich-spreading branches and can reach an average of one meter in height (Sweet, 1830). On branches grow usually corrugated leaves, simple and indivisible, either petiolate or sessile, growing opposite or alternate, and carry simple epidermal hair (trichomes) either asteroid or in bunches. Two types of trichomes appear in *Cistus*, the non-glandular or stellate and the glandular which secrete a resinous exudate, the ladano, to which they owe their distinctive aromatic scent (Gulz et al., 1996). The plants have a terminal or axillary cymose inflorescence, in some species racemose or umbel, with unilateral scorpioid cyme, and by reduction solitary flowers (Demoly and Montserrat, 1993). The flowers are ephemeral, stimulated by the morning light, have either white or pink/purple petals and 3–5 petals. There are numerous stamens, while the ovary has 5 carpels (but can also be 6–12), the style is straight, usually long and inconspicuous, and the stigma is large, discoid with 5–12 lobes (Demoly and Montserrat, 1993). *Cistus* plants have numerous polyhedral seeds with two linear cotyledons. Their chromosome number is *n* = 9 (2*n* = 18) (Demoly and Montserrat, 1993). The plants form ectomychorrizal roots through strict symbiotic associations with various mychorrizal fungal species, mainly belonging to genus *Lactarius* (Comandini et al., 2006). These are characterized by a multilayered mantle and a Hartig net hyphae physiology that involves both epidermal and cortical cell layers (Comandini and Rinaldi, 2008).

The specific botanical characteristics such as are color of petals, number of sepals, and fruit compartments, type of leaf base and size of the styles are the most commonly used in *Cistus* systematic classification (Table S2).

Their general morphological characters as well as their adaptability mechanisms to various harsh environmental conditions will be briefly discussed.

#### Subgenus I: Cistus L

Flower morphology is characteristic in this subgenus. Specifically, each flower consists of five sepals, with pink or purple petals, 80–150 stamens with exine, rugulate pollen about 1.4 μm thick, a long style similar or exceeding the stamens in height with polyspermous placentas (Demoly and Montserrat, 1993).

Within section *Erythrocistus*, resin excreting glandular trichomes appear in *C. albidus, C. creticus* subsp. *creticus*, the short, curled-leaved *C. crispus* (Gulz et al., 1996) and *C. parviflorus*. Peculiarly, *C. creticus* subsp *. eriocephalus* leaves seem to contain only non-glandular trichomes (Paolini et al., 2009). This can explain the different chemical profile between the two sub-species, *C. creticus* subsp *. creticus* (CC) and *C. creticus* subsp *. eriocephalus* (CE). This observation can be of valuable help in applying modern genomic approaches that allow the distinction between the two sub-species in order to isolate and characterize biosynthetic genes leading to the production of active labdane diterpenes present in the former sub-species.

*C. albidus* has bright purple flowers (June to August), *C. creticus* purplish-pink (mid-April to mid-June) and so are those of *C. crispus* (June to August), while *C. parviflorus* has small light pink flowers (Sweet, 1830).

*C. albidus* displays ecotypic differentiation, at least when growing in semi-arid climates, being able to adapt the growth of its branches and leaf dimensions, acquiring the greatest growth under plentiful water availability, while slowing growth and tending to phenotypically converge under drier environments (Grant et al., 2005).

The leaves of the Cretan rock rose *C. creticus* exhibit the phenomenon of seasonal dimorphism, as an adjustment mechanism for acclimatization to the Mediterranean climate. During summertime, when water is limited, brachyblasts are developed that have leaves five-times shorter than the ones in winter, with stomata located abaxially inside crypts (Aronne and Micco, 2001). During wintertime, the newly developed dolichoblasts are fourteen times longer, bearing a bigger number of leaves with stomata distributed across the lower surface.

The curled-leaved *C. crispus* reaches only up to 70 cm in height (Sweet, 1830) and is covered by whitish hair (Sweet, 1830). *C. parviflorus* on the other hand, is one of the tall *Cistus* plants, that can reach a height of 1.5 m. According to Guzmán et al. (2009), *C. parviflorus* is actually classified within the white flowered lineage of the *C. salviifolius* species group.

#### Subgenus II: Leucocistus WILLK

Plants in this subgenus carry white flowers with 3–5 sepals, and have exine pollen around 4.2 microns thick, crosslinked, or shallow mesh foveolae and polyspermous placentas (Demoly and Montserrat, 1993).

In section Ledonia belong the two most widespread *Cistus* species, *C. monspeliensis* and *C. salviifolius*, together with *C. populifolius*. Characteristics of this section are the five sepals, which are either subequal or the two external are longer, and the style being slightly shorter than the stamens (Demoly and Montserrat, 1993). Both *C. monspeliensis* and *C. salviifolius* carry glandular and non-glandular trichomes, while distinctive to *C. populifolius* is the existence of only the glandular type (Gulz et al., 1996). *C. populifolius* is further divided in subspecies *populifolius* and *major* Dunal.

*C. monspeliensis*, also known as the Montpelier rock-rose, is characterized by its aromatic leaves and its small white flowers (Angelopoulou et al., 2001a; Kalpoutzakis et al., 2003). It also exhibits seasonal leaf dimorphism with alternating wide and thin late autumn/early winter and thicker late spring/early summer leaves with larger trichome density, while both types coexist on the same plant during early spring (de Dato et al., 2013). Summer leaves have high leaf mass area and tissue density, low leaf surface area and thick adaxial cuticle, traits that contribute to the plants endurance to drought conditions and resistance to fire (Catoni et al., 2012). As expected, long-term experimental drought conditions during the transition to summer leaves can have significant effect on leaf functioning (de Dato et al., 2013). In a relevant study, over-imposed drought resulted in early leaf litter and a reduction of spring-leaf lifespan, thus a shorter vegetative season, which can have a negative effect on *C. monspeliensis*' survival in Mediterranean shrubland (De Dato et al., 2008; de Dato et al., 2013).

Characteristics of section *Ladanium* include three sepals, large petals and an inconspicuous style (Demoly and Montserrat, 1993). It consists of the laurel-leaved *C. laurifolius*, which is the tallest (1–2 even 3 m) and the very short (50–400 cm) flat-leaved Gum *C. ladanifer*, which is further divided in three subspecies, *ladanifer, africanus* Dans, and *sulcatus* Demoly. Both species carry non-glandular and resin producing glandular trichomes on the abaxial surface of the leaves (Gulz et al., 1996). In *C. ladanifer*, wavy lamina-forming crypts are arranged on the abaxial side, near which non-glandular trichomes are mostly gathered (Tattini et al., 2007).

#### Subgenus III: Hamilioides (WILLK)

The general characteristics of this subgenus are the three sepals, surrounding small, white petals, 30–40 stamens, grooved or fluted-reticulate exine pollen about 2.8 μm thick, and a short style, slightly exceeded the stamens (Demoly and Montserrat, 1993).

*C. clusii*, also called Clusius's rock rose, is a vigorously growing shrub, highly resistant to drought (Pugnaire and Lozano, 1997). It belongs to the *C. clusii* group, comprising one of the two white flowered *Cistus* lineage species, together with *C. munbyi*, and is divided in two subspecies, namely subsp. *clusii* and subsp. *multiflorus* Willk (Guzmán et al., 2009).

## Geographical distribution

### Genus *Cistus L*

*Cistus* plants are extensively distributed in the Mediterranean region, covering most areas from the Canary Islands and Madeira to Caucasus and Israel, colonizing the Iberian, Apennine, Balcan, Crimean, and Anatolian peninsulas and North Africa. In this review we have studied the ten most prominent *Cistus* species, which are widely spread within the Mediterranean basin (Table S1). The geographical distribution of the species and subspecies can be further explored by visiting the following webpage: Interactive Map of *Cistus* distribution. In the next paragraphs we describe the distribution, diversification and habitat preference of these species.

#### Subgenus I: Cistus L

*C. albidus* grows in evergreen shrublands, is partially drought-deciduous (Grant et al., 2005) and a non-strict calcicole (Soriano and Gómez Miguel, 2009) that prefers calcareous and basic soils (De Vega et al., 2008) and woodlands with plenty *Pinus* and *Quercus* compost, in dry areas with altitude up to 1200 m (Guzmán and Vargas, 2010). Phylogenetic studies have shown that it is closely related to the endemic species forming the Canarian *Cistus* lineage, while still forming a monophyletic group with two other purple-flowered species exclusive in the Mediterranean basin, *C. creticus and C. heterophyllus* (Guzmán and Vargas, 2010).

Several populations of *C. creticus* are spread in central-eastern Mediterranean, including Corsica and Sardinia (Falchi et al., 2009) and the island of Crete in Greece (Demetzos et al., 2002a). Among the three *C. creticus* subspecies identified, subsp. *corsicus* is limited to the islands of Corsica and Sardinia (Falchi et al., 2009). More than twenty five populations of subsp. *creticus* are endemic to the coastal areas of Crete (Greece) (Demetzos et al., 2002a). Subspecies *eriocephalus* is exclusive to the Mediterranean area (Demetzos et al., 2001), mainly located on the islands of Corsica, Sardinia (Paolini et al., 2009), and Crete (Demetzos et al., 1997).

*C. crispus* is endemic to southern France, Spain, Iberian, and Apennine Peninsulas and to northwest Africa (Guzmán et al., 2009) and grows in clay or stony soils.

The purple-flowered *C. parviflorus* is a distinctive member of the white-flowered species lineage of *Cistus* that seems to have diverged in the Middle Pliocene (3.13 ± 0.08 Ma) (Guzmán and Vargas, 2010). Though it appears that early divergence of *Cistus* species occurred in the western Mediterranean, *C. parviflorus* is distributed exclusively in the eastern Mediterranean (Guzmán et al., 2009; Guzmán and Vargas, 2010). It prefers dry climates and shrublands with calcicolous soils (Guzmán et al., 2009).

#### Subgenus II: Leucocistus WILLK

Species of *Leucocistus* are widespread in the Mediterranean basin and Madeira, the Canary and Balearic Islands, reflecting their successful adaptation and colonization in Mediterranean habitats (Robles and Garzino, 2000; Guzmán and Vargas, 2005).

*C. monspeliensis* is spread from the western Mediterranean to the Canary Islands and Madeira where it seems to have occurred naturally without any human intervention (Guzmán and Vargas, 2010). The origin of this colonization and further diversification in the Canaries appears to be on the islands of Tenerife and Gran Canaria that have favorable ecological conditions for the growth of the species, and where *C. monspeliensisis* is widely distributed today (Fernández-Mazuecos and Vargas, 2011). Comparative phylogeography was used to demonstrate that the migration of the species to La Gomera and El Hierro occurred via long-distance dispersal from Tenerife to the southwest. *C. monspeliensis* is dominant in evergreen garrigue vegetation, inhabiting acidic, limestone, silicolous and calcareous hills and colonizing areas that are rich in *Quercus* and *Pinus* trees compost or have been disturbed by fire (Angelopoulou et al., 2001a; Guzmán and Vargas, 2005; Guzmán et al., 2009; Catoni et al., 2012). It has also been demonstrated to be a non-strict calcifuge (Soriano and Gómez Miguel, 2009).

*C. populifolius* inhabits areas of the western Mediterranean basin and prefers volcanic and silicolous soils (Guzmán et al., 2009).

*C. salviifolius* is the most widely spread species of the genus *Cistus* around the Mediterranean basin. At least three intercontinental colonizations are responsible for its wide distribution, leading to little geographical isolation with high genetic diversity within populations, but no genetic differentiation between the different populations of *C. salviifolius* (Farley and McNeilly, 2000; Fernández-Mazuecos and Vargas, 2010). The factors that caused the dispersion of *C. salviifolius* around the Mediterranean were mostly ecological, such as the climate and the soil. It grows in silicolous and calcicolous soils and occurs on sandy soils of a wide range of habitats (Guzmán et al., 2009), while it is often located within the understorey in wooded areas (Farley and McNeilly, 2000).

***Section 2: Ladanium (SPACH)***. Natural habitats of *C. ladanifer* are located exclusively in the western Mediterranean. The subspecies of *C. ladanifer* are distributed in close and overlapping geographical regions. It grows in volcanic and silicolous soils in habitats with dry and hot climate (Guzmán et al., 2009).

The adaptation of this species in dry, hot areas is due to its hairy, amphistomatous, and wavy leaves with stomata mostly concentrated in the crypts formed on the abaxial surface of the leaf (Tattini et al., 2007).

*C. laurifolius* prefers silicolous soils and mesic and high altitudes with Mediterranean mountain climate. This ecological preference for habitat has isolated the European and African populations, which were produced by a single, eastward migration event (Fernández-Mazuecos and Vargas, 2010).

#### Subgenus III: *Hamilioides* WILLK

The species in this subgenus are exclusive in the western Mediterranean (Guzmán and Vargas, 2005).

*C. clusii* is highly efficient in surviving in harsh environments colonizing post-fire and perturbed areas (Pugnaire and Lozano, 1997). It prefers calcicolous soils and dry to semi-arid environments, and can grow in high altitudes, up to 1500 m from the coastline (Guzmán et al., 2009).

## Chemical analyses

A large variety of secondary metabolites occurs in different tissues of the 10 *Cistus* species covered in this review. In total, 733 chemical substances have been reported, 397 of which are terpenes (101 monoterpenes, 178 sesquiterpenes, and 118 diterpenes), 162 are of phenypropanoid nature (128 flavonoids, 17 phenolics, and 12 tannins), 24 hydrocarbons, 35 fatty acids, 36 carbonylic compounds, and 18 phytohormones and vitamins (Tables S3, S4). Specifically, *C. albidus* is one of the most studied species and contains 140 terpenes (34 monoterpenes, 101 sesquiterpenes, and 5 diterpenes), 24 phenylpropanoids (18 flavonoids, 2 phenolics, and 4 tannins), 9 hydrocarbons, 24 fatty acids, 7 carbonylic compounds, and 18 phytohormones and vitamins.

In *C. creticus* subsp. *creticus* 92 terpenes (36 monoterpenes, 35 sesquiterpenes, and 21 diterpenes) and 12 phenylpropanoids-flavonoids have been reported. In *C. creticus* subsp. *eriocephalous* 47 terpenes (consisting of 17 monoterpenes, 19 sesquiterpenes, and 11 labdane-type diterpenes) and 2 carbonylic compounds have been detected. The main secondary metabolites identified in *C. clussi* and *C. crispus* are phenylpropanoids: 23 phenylpropanoids (15 flavonoids, 3 phenolics, and 5 tannins) and one labdane-type diterpene for the former and 10 phenylpropanoids (6 flavonoids, 2 phenolics, and 2 tannins) for the latter. In *C. ladanifer*, 72 terpenes (47 monoterpenes, 18 sesquiterpenes, and 7 labdane-type diterpenes), 43 phenylpropanoids (27 flavonoids, 3 phenolics, and 12 tannins) plus an additional 6 carbonylic compounds have been identified.

*C. laurifolius* is a main source of phenylpropanoids (44 flavonoids, 6 phenolics, and 7 tannins), and also contains 4 terpenes (1 labdane-type diterpene and 3 clerodanes) and 1 carbonylic compound. A plethora of studies conducted using *C. monspeliensis* revealed a high content of secondary metabolites, especially terpenoids (22 monoterpenes, 33 sesquiterpenes, and 52 diterpenes). It also produces 17 phenylpropanoids (6 flavonoids, 6 phenolics, and 4 tannins), 20 hydrocarbons, 17 fatty acids, and 10 carbonylic compounds.

In *C. parviflorus*, 99 terpenes (17 monoterpenes, 44 sesquiterpenes, and 38 diterpenes), 19 phenylpropanoids (17 flavonoids and 2 phenolics), 8 hydrocarbons, 5 fatty acids, and 7 carbonylic compounds were identified. *C. populifolius* contains 10 clerodane diterpenes, 10 phenylpropanoids (out of which 1 flavonoid, 1 phenolic compound, and 8 tannins). Another species rich in secondary metabolites, terpenes and phenylpropanoids, is *C. salviifolius:* terpenes consist of 32 monoterpenes, 85 sesquiterpenes and 43 diterpenes, while the phenylpropanoid content includes 39 flavonoids, 8 phenolic compounds, and 9 tannins. Finally, 14 hydrocarbons, 5 fatty acids, and 22 carbonylic compounds complete the metabolic profile of the species. The following sections are devoted to the main chemical constituents of *Cistus* species.

### Teprenes

Metabolite content and volatiles in *Cistus* are influenced by several factors including diurnal, seasonal, ecological, drought, temperature, plant age, and precipitation. Also, depending on the type of trichomes they contain, *Cistus* species can be high producers of monoteprenes and sesquiterpenes, while others in diterpenes and clerodanes.

#### Monoterpenes

Annual presence of several monoterpenes, including α-pinene and limonene, was observed in *C. albidus* grown in Catalonia, Spain (Llusià et al., 2010). Minor amounts were identified mostly in flower tops and other tissues (leaves, petals, sepals) collected from plants that grow in nature, in Italy (Maccioni et al., 2007). High amounts of thymol and carvacrol were exclusive to the pollen (Maccioni et al., 2007). Only a small proportion of oxygenated monoterpenes (0.2%), but no monoterpene hydrocarbons, was identified in *C. albidus* leaves that grow in nature in France (Paolini et al., 2008). In another work, many monoterpenes, including 3-carene, camphene, *cis*-linalool oxide, *cis*-thujone, tricyclene, verbenone, and α-thujene and traces of borneol were detected in Mediterranean *C. albidus* leaves (Ormeño et al., 2007). Similarly, many monoterpenes and oxygenated monoterpenes were found in *C. creticus* subsp. *creticus* leaf and essential oil, collected from different regions of Crete in Greece (Demetzos et al., 1994b, 1995). The most abundant leaf volatiles collected from aerial parts of Cretan *C. creticus* subsp. *eriocephalus* from different regions of Corsica (France) and North Sardinia were monoterpenes, especially myrcene and limonene (Paolini et al., 2009). Several monoterpenes were also present in essential oils, but in significantly smaller concentrations (Demetzos et al., 1997). Similarly, monoterpene compounds constitute the majority of terpenes identified in essential oils of several *C. ladanifer* populations found in northern Portugal (Ramalho et al., 1999; Gomes et al., 2005; Teixeira et al., 2007), Spain (Alías et al., 2012), Morocco (Zidane et al., 2013), and south France (Mariotti et al., 1997; Robles and Bousquet-Mélou, 2003).

The monoterpene phenol carvacrol was a major constituent of Tunisian *C. monspeliensis* leaves and essential oils, while other polyphenolic compounds such as diisobutyl ester (phthalic acid) and benzyl benzoate were also strongly represented (Jemia et al., 2013; Loizzo et al., 2013). Moreover, carvacrol and α-terpineol were the only monoterpene compounds isolated from leaf essential oil of *C. monspeliensis* plants found in Crete (Greece) (Angelopoulou et al., 2001a, 2002), while several phenolic acid derivatives were identified in plants grown in Spain (Barrajón-Catalán et al., 2011). Several monoterpenes, including hydrocarbons and oxygenated monoterpenes were identified as the major components in leaves and essential oils of Tunisian and French *C. monspeliensis* plants (Rivoal et al., 2010; Jemia et al., 2013; Loizzo et al., 2013). Monoterpenes were 14.47% of the total essential oil extracted from Cretan *C. parviflorus* (Demetzos et al., 1990b). The highest concentration of monoterpenes in the essential oil from *C. parviflorus* plants grown in Crete were mainly oxygenated monoterpenes while no traces of monoterpene hydrocarbons were reported. Carvacrol was identified as the major constituent in all samples (Angelopoulou et al., 2001b). Mostly oxygenated monoterpenes and only a small fraction of monoterpene hydrocarbons have been detected in a large number of *C. salviifolius* populations from Crete (Greece) and Tunisia (Demetzos et al., 2002b; Loizzo et al., 2013).

#### Sesquiterpenes

Chemical analysis of *C. albidus* tissue and essential oil preparations in north-eastern Spain, France and Italy showed high content of sesquiterpenes, in particular, β-sesquiphellandrene, β-caryophyllene, β-bourbonene, α-zingiberene, and germacrene D (Robles and Garzino, 1998; Maccioni et al., 2007; Paolini et al., 2008; Llusià et al., 2010). Among them, *α*-zingiberene and germacrene D were the most abundant, while *epi*-10-γ-eudesmol was detected uniquely in flowers, 1-*epi*-cubenol only in leaves, and β-himachalene exclusively in flower tops (Maccioni et al., 2007). In a study conducted on Mediterranean *C. albidus* (Ormeño et al., 2007), germacrene D, *ar*-curcumene and *allo*-aromadendrene constituted the highest content of emissions from leaves. Analyses of essential oils secreted from the resin of the Cretan plant *C. creticus* subsp. *creticus* revealed the presence of several sesquiterpenes and oxygenated sesquiterpenes (Demetzos et al., 1994b, 1999). The sesquiterpene fraction was the largest in the essential oils of *C. creticus* subsp. *eriocephalus* endemic in the Cretan flora in Greece (Demetzos et al., 1997).

High concentrations of sesquiterpenes have been measured in aerial parts and essential oils of several *C. ladanifer* populations in France, Portugal, Spain and Morocco, with vidiflorol being the most abundant molecule (Mariotti et al., 1997; Ramalho et al., 1999; Robles and Bousquet-Mélou, 2003; Gomes et al., 2005; Teixeira et al., 2007; Zidane et al., 2013). Analysis of essential oil composition of *C. monspeliensis* grown in Tunisia led to the identification of accountable amounts of oxygenated sesquiterpenes and sesquiterpene hydrocarbons (Loizzo et al., 2013). Another study conducted with plants at the same site detected a large variety of sesquiterpenes but at low concentrations (Jemia et al., 2013). Several sesquiterpenes were also isolated from *C. monspeliensis* plants naturally growing in areas of France and Greece (Robles and Garzino, 2000; Angelopoulou et al., 2001a, 2002; Rivoal et al., 2010). Sesquiterpene compounds identified in *C. monspeliensis* plants located in Crete (Greece), accounted for a total content of 38.5% in leaf and 6.6% in fruit essential oil (Angelopoulou et al., 2001a).

Mainly oxygenated sesquiterpenes were identified in high percentages in the essential oil of nine populations of *C. parviflorus* endemic to the island of Crete, with caryophyllene oxide and α-epi-cadinol being the most abundant (Angelopoulou et al., 2001b). High concentrations of sesquiterpenes were also found in aerial parts and essential oils of several *C. salviifolius* Cretan and Tunisian populations, among which oxygenated sesquiterpenes were the most abundant (Demetzos et al., 2002b; Loizzo et al., 2013).

#### Diterpenes

Only a small fraction of the essential oil composition of *C. albidus* plants collected in south France and Spain contained diterpenes (Paolini et al., 2008; Llusià et al., 2010). Among them the largest concentration was measured for the labdane-type compound 13-*epi*-manoyl oxide (Paolini et al., 2008). Chemical analysis of the aerial parts and essential oil of *Cistus creticus* subsp. *creticus* plants native to Crete, Greece, revealed the presence of several labdane-type diterpenes (especially manoyl oxide and 13-*epi*-manoyl oxide) (Demetzos et al., 1990a, 1994b,c, 1995, 1999, 2002c; Anastasaki et al., 1999; Falara et al., 2010). The first metabolomic analysis of isolated trichomes from this species (Falara et al., 2010) detected the presence of several labdane-type diterpenes (8,13-*epoxy*-15,16-dinorlabd-12-ene, 13-*epi*-manoyl oxide, 3β-hydroxy-13-*epi*-manoyl oxide, labd-7,13-dien-15-ol, labd-7,13-dien-15-yl acetate, 3β-acetyl-13-*epi*-manoyl oxide, labd-13-en-8α,15-diol, and labd-13-en-8α-ol-15-yl acetate). Further experiments in this study demonstrated that the production of labdane-type diterpenes in *Cistus* is trichome-specific (Falara et al., 2010).

The labdane-type diterpenes manoyl oxide (9.9%) and 13-*epi*-manoyl oxide (3.4%) constituted a significant fraction of components in Cretan *C. creticus* subsp. *eriocephalus* leaves' extracts (Demetzos et al., 1997). The diterpenes that comprise the major diterpene fraction of Spanish *C. ladanifer* are 6-acetoxy-7-oxo-8-labden-15-oic acid, 7-oxo-8-labden-15-oic acid and oxocativic acid (Alías et al., 2012), while more labdane-type diterpenes were found in other studies conducted in France, Portugal and Spain (Mariotti et al., 1997; Gomes et al., 2005; Tomás-Menor et al., 2013).

The labdane-type diterpene 6β,8-dihydroxy-*ent*-13*E*-labden-15-oic acid (laurifolic acid) (De Pascual Teresa et al., 1986) as well as the clerodanes salmantic acid and its methyl ester, salmantidiol (de Pascual Teresa et al., 1983) were isolated and characterized from *C. laurifolius* extracts. Leaves and essential oils from *C. monspeliensis* plants collected in France, Greece, Morocco, and Tunisia were rich in diterpenes (Berti et al., 1967; Robles and Garzino, 2000; Angelopoulou et al., 2001a, 2002; Oller-López et al., 2005; Loizzo et al., 2013). The labdane-type diterpenes 13-*epi*-manoyl oxide, manoyl oxide and epimers were the most abundant in all studies. In *C. monpeliensis* leaves from plants grown in Tunisia, diterpenes constituted only a small fraction (3.8%) of the total secondary metabolite content (Jemia et al., 2013). The analysis of the aerial parts and essential oils of *C. monspeliensis* plants grown in Greece revealed the existence of several clerodanes (Berti et al., 1970; Angelopoulou et al., 2001a; Demetzos et al., 2001; Kalpoutzakis et al., 2003). The labdane-type diterpenes manoyl oxide mixture of isomers and 13-*epi*-manoyl oxide were detected in all the nine populations of *C. parviflorus* grown in Crete (Angelopoulou et al., 2001b). In a study by Demetzos et al. (1990b), 37.78% of the essential oil constituents were diterpene compounds. Clerodanes were the only diterpenes detected and characterized from *C. populifolius* (Urones et al., 1994, 1995).

Diterpenes were also a significant fraction of the metabolites identified in *C. salviifolius* populations. The most abundant diterpene in plants endemic in Tunisia was manoyl oxide (Loizzo et al., 2013). In Cretan populations, manoyl oxide and 13-*epi*-manoyl oxide were also detected, but *cis*-ferruginol was the most abundant metabolite (Demetzos et al., 2002b).

### Phenylpropanoids

Flower scent is a vital strategy that plants use for attracting pollinators and ensuring their reproduction and survival (Gang, 2005). Volatile phenylpropanoids have a significant role among the compounds emitted by the plants in order to contribute to this aroma. Moreover, as a defensive mechanism of the plants against high solar radiation and drought, the content of the antioxidant flavonoids in *Cistus* species is highly variable. In most species, increased concentrations of flavonoids are observed during summer and in younger leaves. In addition to emissions, light, especially UV-B radiation, positively influences in a periodic manner the absorbing capacity of epicuticular phenolic substances in *C. creticus*, contributing to the plants photoprotective mechanism (Stephanou and Manetas, 1997). High concentrations of the non-volatile group of tannins have also been measured in various *Cistus* species, including the hydrolysable gallic and ellagic acids and ellagitannins (Barrajón-Catalán et al., 2011).

#### Flavonoids

Several flavonoids, including quercetin, myricetin, kaempferol, apigenin and their derivatives, were isolated from leaves and resin of Cretan *C. creticus* subsp. *creticus* (Demetzos et al., 1989, 1990a). The same compounds were also detected in young leaves of Mediterranean *C. laurifolius* (Orhan et al., 2013), aerial parts of Spanish *C. salviifolius* plants (Danne et al., 1994; Saracini et al., 2005; Qa'Dan et al., 2006; Barrajón-Catalán et al., 2011; Loizzo et al., 2013; Tomás-Menor et al., 2013) and aqueous extracts of Spanish *C. crispus* plants (Barrajón-Catalán et al., 2011). The anti-oxidant polyphenolic flavonoids catechin, gallocatechin and several of their derivatives, as well as other flavonols, flavanols, flavonoid glycosides and proanthocyanidin compounds have been identified in *C. albidus* leaves (Qa'Dan et al., 2003; Barrajón-Catalán et al., 2011; Tomás-Menor et al., 2013). Flavonoid related compounds were detected in aerial parts of *C. clusii* grown naturally in Spain, including kaempferol diglucoside and the (−)-(epi)gallocatechin-(epi)gallocatechin dimer (Barrajón-Catalán et al., 2011). The latter compound was also identified in the leaves of field-grown *C. clusii* plants together with significant amounts of flavan-3-ols and the simpler proanthocyanidins (Hernández et al., 2011).

Kaempferol and its derivatives were also detected in *C. ladanifer*, together with several flavonoids belonging to the groups of flavones, flavonols and flavon-3-ols (Chaves et al., 1993; Fernández-Arroyo et al., 2010; Barrajón-Catalán et al., 2011). In addition, plants of this species accumulate these flavonoids (particularly acylated kaempferol 3-O-glycosides) in the non-glandular trichomes.

Many biologically active flavonoids were also identified in tissue extracts of *C. laurifolius* (Vogt et al., 1988; Vogt and Gerhard Gul, 1994; Enomoto et al., 2004; Küpeli et al., 2006; Sadhu et al., 2006; Küpeli and Yesilada, 2007; Akkol et al., 2012; Orhan et al., 2013). The flavonol aglycones quercetin 5,3′–dimethyl ether and quercetin 3,5,3′-trimethyl ether, detected in the leaf resin of young *C. laurifolius* leaves, have been identified and characterized (Vogt et al., 1988). Variable amounts and types of flavonoid compounds were identified in hexane leaf extracts of *C. monspeliensis* plants naturally growing in Spain and Tunisia (Pomponio et al., 2003; Barrajón-Catalán et al., 2011; Jemia et al., 2013), while only few flavonoid substances were isolated from *C. populifolius* aqueous extracts (Barrajón-Catalán et al., 2011). *C. parviflorus* leaves' resin contained several flavonoids including kaempferol, quercetin methyl ethers and 6- and 8-*O*-methylated flavonols (Vogt et al., 1987).

#### Phenolic acids and tannins

Gallic acid and hexahydroxydiphenoyl-glucose, together with variable gallic acid-derived hydrolysable ellagitannins were identified in *C. albidus, C. clusii, C. crispus, C. creticus, C. ladanifer, C. laurifolius, C. monspeliensis, C. populifolius*, and *C. salviifolius* leaf samples collected in Spain, with evident ecological variation (Barrajón-Catalán et al., 2011). *C. laurifolius*, is a high producer of tannins throughout the year, especially in younger leaves (Ammar et al., 2004). Several phenolic acids and derivatives have also been detected in aerial parts of *C. albidus, C. clusii, C. crispus, and C. ladanifer* (Ramalho et al., 1999; Qa'Dan et al., 2003; Teixeira et al., 2007; Barrajón-Catalán et al., 2011). In addition, high amounts of elemicin and several phenolic compounds were detected in *C. salviifolius* leaves (Loizzo et al., 2013).

### Hydrocarbons

Among volatile organics isolated from leaves of *C. albidus* collected from southern Catalonia, Spain were docosane, octacosane, and tetracosane (Llusià et al., 2010). Linear hydrocarbons including *n*-tetradecane and *n*-hexadecane, were identified exclusively in the pollen and not in other flower parts or leaves of *C. albidus* growing in Italy in late spring (Maccioni et al., 2007). Only tricosane was detected in the essential oil of *C. albidus* plants growing in France (Paolini et al., 2008). Several hydrocarbons such as heptacosane, nonacosane, pentacosane, and tricosane were also produced by *C. monspeliensis* endemic in the South of France, Greece, and Tunisia (Robles and Garzino, 2000; Angelopoulou et al., 2001a; Jemia et al., 2013; Loizzo et al., 2013). Tertadecene, pentacosane, pentadecane, neophytadiene, heptadecene, docosane, heneicosane, and dodecane were all detected in *C. parviflorus* (Angelopoulou et al., 2001b), while neophytadiene and pentacosane were identified in several Greek and Tunisian populations of *C. salviifolius* (Demetzos et al., 2002b; Loizzo et al., 2013).

### Fatty acids

Several fatty acids were identified in aerial parts and essential oils of *C. albidus* growing in north-eastern Spain, including tetradecanoic acid and pentadecanoic acid (Llusià et al., 2010; Müller et al., 2014). Fatty acid composition of *C. albidus* seeds was studied in both young and old plants. High concentrations of linoleic acid, and generally polyunsaturated as well as very long chain saturated fatty acids were found in seeds of the older plants (Müller et al., 2014). Various fatty acids and esters were identified as minor or major components of aerial parts and essential oil content of French, Greek, and Tunisian *C. monspeliensis, C. creticus* subsp. *creticus*, and *C. salviifolius* plants (Demetzos et al., 1994b, 2002b; Robles and Garzino, 2000; Angelopoulou et al., 2001a; Jemia et al., 2013; Loizzo et al., 2013).

### Carbonylic compounds

Aliphatic aldehydes including octanal, nonanal, decanal and 6-methyl-5-hepten-2-one were present only in the pollen and not in other flower parts or leaves of *C. albidus* plants growing in Italy in late spring (Maccioni et al., 2007). Nonanal, decanal, undecanal, and dodecanal were detected in the essential oil of *C. albidus* plants growing wild in France (Paolini et al., 2008). Nonanal and β-ionone were also detected in *C. creticus* subsp. *eriocephalus* leaves. Among the secondary metabolites produced by French, Greek and Tunisian *C. monspeliensis* plants, the norisoprenoids (E)-β-damascenone and members of the ionone family, used in the fragrance industry, as well as several other carbonylic compounds were identified (Robles and Garzino, 2000; Angelopoulou et al., 2001a, 2002; Demetzos et al., 2002b; Jemia et al., 2013; Loizzo et al., 2013).

### Phytohormones and vitamins

The production of phytohormones and vitamins were studied in *C. albidus* seeds (Müller et al., 2014), leaves, and flowers (Oñate and Munné-Bosch, 2010), in relation to plant maturity. The main vitamin E compound in *C. albidus* seeds was α-tocopherol, whose content was higher in mature plants than the younger ones (Müller et al., 2014). Similarly, seeds of mature plants had higher concentrations of both salicylic acid and jasmonic acid, which was not the case in flowers and leaves, where no concentration differences were observed between the age groups (Oñate and Munné-Bosch, 2010). The adaptability of the species to drought involved an increase in abscisic (ABA) and ascorbic acid (AA) levels, as well as leaf H_2_O_2_ concentrations, localized mainly in mesophyll cell walls, xylem vessels, and differentiating sclerenchyma cells (Jubany-Marí et al., 2009). Recovery from drought implicated the readjustment of ABA, AA, and H_2_O_2_ to their basal concentrations (Jubany-Marí et al., 2009). Moreover, efficient acclimation to drought was only achievable in the first of three consecutive cycles of experimental drought and re-watering applications of *C. albidus* plants (Galle et al., 2011).

*C. creticus* is also a highly drought-resistant plant, and was used as a model to study drought-induced changes in the expression of genes encoding enzymes involved in isoprenoid biosynthesis, as well as the corresponding metabolites (chlorophylls, carotenoids, tocopherols, and abscisic acid) and endogenous concentrations of other growth regulators (jasmonic and salicylic acids, JA and SA, respectively) (Munné-Bosch et al., 2009). Among the genes studied, those encoding homogentisate phytyl-transferase (HPT) and 9-*cis*-epoxycarotenoid dioxygenase (NCED) were induced even at early stages of drought, and were strongly correlated to the levels of the corresponding metabolites. The simultaneous increase in concentrations of ABA and α-tocopherol (but not JA and SA), led the authors to suggest that the genes encoding HPT and NCED may play a key role in drought stress resistance by modulating ABA and tocopherol biosynthesis (Munné-Bosch et al., 2009).

## Biological functions

Various preparations from *Cistus* species have traditionally been used as remedies in folk medicine around the Mediterranean basin, especially in Greece, Italy, Spain, and Turkey. The targeted conditions and diseases include anxiety, arthrosis, asthma, bronchosis, various types of cancer, bacterial and fungal infections, cardiopathies, catarrh, corn, diarrhea, duodenosis, dysendery, dyspnea, fracture, gastrosis, headache, hepatosis, hernia, hysteria, induration, infection, inflammation, insomnia, leukorrhea, myalgia, neuralgia, osteoarthritis, polyp, proctosis, rhinosis, sore, spasm, splenosis, ulcer, uterosis (Duke et al., 2008). A considerable amount of studies have thus explored the pharmacological properties of the resin secreted by *Cistus* leaves. These properties include allergenic, anti-aggregant, anti-leukemic, anti-oxidant, anti-peroxidant, anti-proliferant, anti-radicular, antiseptic, anti-ulcer, astringent, bactericide, candidicide, cardio-protective, cytotoxic, dermo-protective, dipeptidylpeptidase-IV inhibitor, alanyl-aminopeptidase inhibitor, diuretic, emmenagogue, expectorant, fungicide, gastro-protective, hemostat, myorelaxant, nervine, purgative, revulsive, sedative, spasmolytic, stimulant, vulcenary (Duke et al., 2008). Below, we discuss some of the most studied biological functions of *Cistus* species and the chemical nature of the potent corresponding compound groups produced by the plants.

### Antibacterial, antifungal

Organic and aqueous leaf extracts of *C. monspeliensis*, and also *C. villosus (=incanus)*, growing naturally in Morocco and Tunisia were shown to have antimicrobial and antifungal properties that were mostly active against *Staphylococcus aureus, Enterococcus hirae*, and *Pseudomonas aeruginosa* and the yeast *Candita glabrata* (Bouamama et al., 2006). Flower extracts of *C. monspeliensis* were active against gram-positive bacteria species of genus *Staphylococcus* and had significant growth-inhibitory effects on *Staphylococcus epidermis* (Sassi et al., 2007). Furthermore, a *cis*-clerodane diterpene isolated in large amounts and characterized from *C. monspeliensis* was very active against *Staphylococci* species and had four times higher activity than the labdane-type diterpene sclareol (Kolocouris et al., 2001).

Labdane-type diterpenes mainly represented by *ent*-13-*epi*-manoyl oxide, manoyl oxide and its isomers were found in significant concentrations in hexane extracts of leaves (16.6%) and fruits (25%) of *C. monspeliensis* growing in Crete, Greece (Angelopoulou et al., 2001a). Antimicrobial and antifungal activities of *C. creticus* subsp. *creticus* and subsp. *eriocephalus* have been evaluated (Chinou et al., 1994; Demetzos et al., 1995, 1997; Anastasaki et al., 1999; Bouamama et al., 1999; Hutschenreuther et al., 2010). The essential oil extracted from leaves of *C. creticus* subsp. *eriocephalus*, which has manoyl oxide and 13-*epi*-manoyl oxide as its main constituents, exhibited a rather weak activity against *E. coli* and *P. aeruginosa*, moderate against *Candita albicans, Micrococcus luteus*, and *S. epidermidis*, and most active against *S. aureus and Bacillus subtillis* (Demetzos et al., 1995), while the volatile oil fraction had a high *in vitro* activity against *Borrelia burgdorferi sensu stricto* (Hutschenreuther et al., 2010). The antimicrobial activity of manoyl oxide extracts from *C. creticus* subsp. *creticus* fruits, leaves and resin was found dose-dependent against gram-positive *Staphylococci* species, inactive against gram-negative bacteria (Anastasaki et al., 1999; Demetzos et al., 1999, 2001, 2002c).

Among several tested labdane-type diterpenes, labd-13-en-8α,15-diol was the only diterpene reported to be active against *Candida albicans*, while labd-7,13-dien-15-ol did not exhibit any antibacterial or/and antifungal activity (Chinou et al., 1994; Bouamama et al., 2006). Several semisynthetic labdane-type diterpenes from the resin “ladano” of *C. creticus* were reported for their antimicrobial activity both against gram positive and gram negative bacteria and also against pathogenic fungi with the highest effects exhibited by two chloroethyl carbamidic esters derivatives (Kalpoutzakis et al., 2001). *C. ladanifer* and *C. populifolius* aqueous extracts had antibacterial activity against *E. coli* (Barrajón-Catalán et al., 2010).

Antimicrobial properties have been demonstrated for several phenolic monoterpenes, such as thymol and carvacrol, and other carbonylic and phenolic compounds identified among *C. creticus* and *C. albidus* volatiles (Maccioni et al., 2007; Hutschenreuther et al., 2010). Phenolic compounds present in extracts from *C. ladanifer* aerial tissues displayed antifungal activity against *Candida* species (Barros et al., 2013) in a dose-dependent manner (Barrajón-Catalán et al., 2010). Strong antimicrobial activity against gram-positive bacteria was also demonstrated for *C. ladanifer*, and against gram-negative bacteria for *C. populifolius* (Barrajón-Catalán et al., 2010).

### Antiviral

The polyphenol rich extract CYSTUS052 derived from *C. incanus* was shown to exhibit potent anti-influenza virus activity without causing toxic side effects or inducing viral resistance (Ehrhardt et al., 2007). The first clinical study showed that *C. incanus* extract (CYSTUS052) could be applied for the treatment of upper and lower respiratory tract infections (Kalus et al., 2009). Moreover, *C. ladanifer* and *C. populifolius* extracts were able to inhibit the replication of the vesicular stomatitis virus (VSV) (Abad et al., 1997).

### Antioxidant

*Cistus* species rich in phenolic compounds, especially flavonoids, demonstrate significant antioxidant properties. Preparations of *C. creticus, C. incanus, C. libanotis, C. salviifolius, C. monspeliensis, C. parviflorus, C. laurifolius, C. ladanifer*, and *C. populifolius* aqueous extracts were able to generate strong antioxidant activities in a dose-dependent manner, using several free radical scavenging methods (Attaguile et al., 2000; Sadhu et al., 2006; Teixeira et al., 2007; Amensour et al., 2010; Barrajón-Catalán et al., 2010; Alsabri et al., 2012; Loizzo et al., 2013). Among all studied species, *C. monspeliensis* appeared to have the highest antioxidant activity (Attaguile et al., 2000, 2004; Loizzo et al., 2013). Potential protective and antioxidant activity was suggested for the pollen of *C. incanus*, after evaluating the induction of anti-estrogenic properties and differential expression of genes involved in the apoptosis pathway and chemotaxis of mice fed with rich in pollen bees in Croatia (Sarić et al., 2009).

Plant-derived remedies for human use need to be carefully prepared in order to result in active antioxidant substances. Indeed, *C. incanus* beverages exhibit decreased amounts of phenolic substances and reduced antioxidant activity if an incorrect selection of brewing process parameters (brewing water, temperature, and duration) is made (Riehle et al., 2013).

### Cytotoxic/anticancer

Cytotoxic activity of shoot extracts from an *in vitro* culture of *C. creticus* subsp. *creticus* against human HeLa cells was shown (Skorić et al., 2012). Shoot extracts (rich in labdane diterpenes) were able to exert cytotoxic activity on HeLa (cervix), MDA-MD-453 (breast), and FemX (melanoma) cancer cells. Cytotoxic and antitumor activities of the bioactive, highly lipophilic, and naturally produced labdane-type diterpenes was improved by incorporating them in liposomal formulations, a process that constitutes these compounds suitable for testing in *in vivo* experiments (Kyrikou et al., 2005; Matsingou et al., 2005; Hatziantoniou et al., 2006). The labdane-type diterpene sclareol, which is used today as a certified drug, has antitumor activity against human breast cancer cell lines and enhances the activity of known anticancer drugs (Dimas et al., 2006). Sclareol is activated through a p53-independent mechanism that targets the G_1_ phase of the cell cycle and therefore apoptosis of human cancer cells is induced through the activation of caspases (Mahaira et al., 2011).

Among nine labdane-type diterpenes isolated from the resin of *C. creticus* subsp. *creticus*, labd-13-en-8α-ol-15-diol was active against 13 of the 14 cell lines, while labd-7,13-dien-15-ol showed activity only in HL60 human promyelocytic leukemia cells (Dimas et al., 1998). Pure *ent*-13-*epi*-manoyl oxide and mixtures of manoyl oxide isomers isolated from *C. monspeliensis* and *C. creticus* aerial parts of plants from Crete were shown to exert moderate cytotoxic activity (Angelopoulou et al., 2001a; Demetzos et al., 2001). Their activity was evaluated against nine leukemic cell lines, where all tested compounds caused growth inhibition at the highest tested concentration of 10^−4^ M (Angelopoulou et al., 2001a). In a previous study, however, *ent*-13-*epi*-manoyl-oxide did not show any antiproliferative effect on the tested human leukemia cell lines (Demetzos et al., 1994a). A dose- and time- dependent inhibition of DNA synthesis by ent-3β-hydroxy-13-*epi*-manoyl oxide, was associated with its conversion to a thiomidazolide derivative (Dimas et al., 1999). Both sclareol and the thiomidazolide derivative induced apoptosis of T-cell leukemic cell lines (Dimas et al., 2001). A number of labdane-type diterpenes isolated from aerial parts of *C. creticus* in Greece, including sclareol, were tested *in vitro* for their cytotoxic activity against human rhinopharynx cancer, murine leukemia and human bronchial epidermoid carcinoma cell lines (Chinou et al., 1994). Among the tested diterpenes, labd-13-en-8α-ol-15-diol was the only one unable to exhibit nearly any cytotoxic activity. Labd-7,13-dien-15-ol, 8,13-epoxylabd-14-ene (manoyl oxide), 13-*epi*-8,13-epoxylabd-14-ene (13-*epi*-manoyl oxide) caused moderate inhibition against the proliferation of the cell lines, while labd-13-en-8α-ol-15-yl acetate, labd-14-en-8,13-diol (sclareol) and 13-*epi*-sclareol were highly active (Chinou et al., 1994). Moreover, the *cis*-clerodane (+)-19-acetoxy-*cis*-clerodan-3-en-15-oic acid, isolated from leaf extracts, was tested against several human leukemic lines and did not exhibit any activity (Demetzos et al., 2001). However, none of the diterpenes tested in the above-mentioned studies from Cretan *C. creticus* had any anti-inflammatory activity or any effect on skin repair (Demetzos et al., 2001).

Three flavonoids were also tested against eleven leukemic cell lines. Myricetin had no activity, a myricetin ether that was isolated from the hexane extract of *C. monspeliensis* exhibited significant cytostatic and cytotoxic activities, and its 3′,5-diacetyl derivative, which was chemically synthesized, had lower cytotoxic activity (Dimas et al., 2000).

### Myorelaxant

*Cistus* extracts have been traditionally used in Mediterranean countries for the treatment of diarrhea, peptic ulcers and as antispasmodic agents. Myorelaxant effects of *C. incanus* and *C. monspeliensis* aqueous extracts were illustrated on strips of longitudinal smooth muscle of rat ileum and aorta in a concentration-dependent and reversible manner (Attaguile et al., 2004). The authors attributed the inhibition of intestinal mobility by polyphenolic compounds to the high concentrations of flavonoids produced by *Cistus* plants (Attaguile et al., 2004). Moreover, *C. populifolius* and *C. ladanifer* aqueous extracts were evaluated *in vitro* on animal models and their significant, dose-dependent spasmolytic (Sánchez de Rojas et al., 1995) and analgesic effects (de Andrés et al., 1999) were demonstrated.

### Toxicity

Some secondary metabolites produced by *Cistus* sp. exert toxic effects on mammals. The most toxic compounds are gallic acid and tannins, which are detrimental to liver and kidneys. Several cases of lethal toxicoses in cattle have been reported, caused by ingestion of *Cistus* sp., including *C. salviifolius* (Yeruham et al., 2002). Convulsions and lipofuscinosis in the central nervous system have been reported in sheep as directly linked to grazing on *Cistus* sp. (Riet-Correa et al., 2009). In addition, flavonoid-rich extract of *C. laurifolius* was responsible for degenerative effects on the periferic nerval system causing convulsive syndrome in mice (Bregante et al., 1981).

As discussed earlier in this section, many secondary metabolites produced by *Cistus* sp. exhibit toxicity and therefore can be used in low concentrations in order to exert their biological functions. Direct consumption of the plants or their extracts (as tea infusions) in larger amounts can become harmful and even lethal.

## Biosynthesis of compounds of interest

### Biosynthetic pathway for terpenes including labdane-type diterpenes

Isoprenoids are one of the largest classes of metabolites with more than 50,000 representatives identified to date in existing organisms with a central role in both primary and specialized metabolism (Thulasiram et al., 2007). Despite the fact that the building block of all isoprenoids is a C5 isoprene unit, they exhibit an extensive diversity in terms of their size and structure that reflects a wide diversity in their physiological roles. Isoprene is the smallest known 5-carbon terpene, a volatile emitted by the foliage of many tree species. Isoprene emission is considered to enhance tolerance against heat stress and to confer resistance to reactive oxygen species. Monoterpenes are C10-carbon, highly volatile terpenes that contribute to plant odors; they are among the main constituents of flower aromas with an essential role in pollinator attraction. Sesquiterpenes (C15) are also volatile compounds known to play a signaling role in plant defense mechanisms, directly as repellants for herbivores or indirectly through the attraction of herbivore predators and/or pathogens. Diterpenes (C20) are produced by plants for defense purposes (phytoalexins), in addition to serving as precursors of plant hormones (e.g., tocopherols and gibberellins). Triterpenes (C30) are synthesized by the head to tail condensation of C15-carbon terpenes and they are the precursors of phytosterols, brassinosteroids, phytoalexins and waxes. Carotenoids are C40-carbon terpenes that contribute to the pigment of flowers and fruit and play an essential role in pollination and seed dispersal as well as protection against UV light. Higher orders of isoprenoids, the polyprenyls, are used for co-translational modification of proteins called prenylation that promotes their interaction with cell membranes.

There are two distinct pathways responsible for the biosynthesis of terpenes (Figure S1) in the plant cell (Vranová et al., 2012). The mevalonate pathway (MVA) that operates in the cytosol and starts from acetylCoA, and the 2-C-methyl-d-erythritol 4-phosphate (MEP) pathway that operates in the plastid and is initiated by C-5 sugars. Their names come from intermediate precursors of the pathways; the mevalonate in the cytosolic pathway and the 2-C-methyl-d-erythritol 4-phosphate in the plastidial pathway. The final product of both metabolic routes is isopentelyl pyrophosphate (IPP), the universal precursor of all isoprenoids produced in the plant cell. IPP gets isomerized to dimethylallyl pyrophosphate (DMAPP) by the enzyme isopentenyl pyrophosphate isomerase.

In the MVA pathway, acetyl-CoA from the citric acid cycle undergoes condensation with another acetyl-CoA to produce acetyloacetyl-CoA via the enzyme acetyl-CoA transferase (AACT). Next, HMG-CoA synthatase catalyzes the condensation of one more molecule of acetyl-CoA with acetyloacetyl-CoA to form 3-hydroxy-3-methyl-gloutaryl-CoA (HMG-CoA). HMG-CoA is then reduced to mevalonate by the HMG-CoA reductase (HMGR) using NADPH as co-factor. Mevalonate kinase (MK) activity yields 5-phospho-mevalonate, which then gets decarboxylated to produce IPP. IPP is then isomerized to DMAPP by the enzyme isopentenyl pyrophosphate isomerase (Flesch and Rohmer, 1988; Rohmer et al., 1993).

The first step of the plastidial pathway involves the condensation of pyruvate with 3-phosphoglycerinaldehyde to produce 1-deoxy-D-xylulose 5-phosphate (DXP) (McGarvey and Croteau, 1995). This reaction is catalyzed by 1-deoxy-5D-xylulose synthase (DXS) that functions as a transacetolase (Lange et al., 1998; Lange and Croteau, 1999; Eisenreich et al., 2001). Then 1-deoxy-D-xylulose 5-phosphate is converted to 2-C-methyl-D-erythritol 4-phosphate (MEP) via the reductase DXR (Takahashi et al., 1998). MEP is then converted to 2-C-methyl-D-erythritol 2,4-cyclodiphosphate (MECPP) in three sequential steps. MEC is converted to 1-hydroxy-2-methyl-2-(E)- butenyl 4-diphosphate by the enzyme 1-hydroxy-2-methyl-2-(E)- butenyl 4-diphosphate synthase (HDS), which is then reduced to 1-hydroxy-2-methyl-2-(E)-butenyl 4-diphosphate via the enzyme 1-hydroxy-2-methyl-2-(E)-butenyl 4-diphosphate reductase (HDR). 1-hydroxy-2-methyl-2-(E)-butenyl 4-diphosphate is finally converted to IPP with the enzyme HMBPP reductase. IPP is then isomerized to DMAPP (Lichtenthaler, 1999).

In both cytosolic and plastidic compartments, IPP and DMAPP are used by prenyltransferases to produce prenyl-diphosphates, the universal precursors of all isoprenoids. A head to tail condensation of IPP and DMAPP results in the formation of geranyldiphosphate (GPP) or neryldiphosphate (NPP), the *trans* and *cis* prenyldiphosphate precursor of monoterpenes, respectively. Sequential addition of one more molecule of IPP by farnesyl-diphosphate synthase results in the formation of all-*trans* or *cis*-trans farnesyldiphosphate (*ee*-FPP and *zz*-FPP), the precursors of sesquiterpenes. For the synthesis of the precursor of diterpenes, geranylgeranyldiphosphatase (GGDP), the addition of one more molecule of IPP is required that is catalyzed by the prenyltransferase geranylgeranyldiphosphate synthase. In *C. creticus* subsp. *creticus* two genes that encode active GGDP synthases, *CcGGDPS1* and *CcGGDPS2*, were cloned and functionally characterized (Pateraki and Kanellis, 2008), as also the DXR cDNA from trichomes (Pateraki and Kanellis, 2004).

The prenyldiphosphate precursors are converted to the basic terpene skeletons through the activity of a class of enzymes called terpene synthases. In the lower orders of terpenes these enzymes are further categorized into monoterpene, sesquiterpene and diterpene synthases. The basic skeletons are often further processed by a variety of enzymes including acyltransferases, hydroxylases and dehydrogenases to produce the thousands of different terpenes encountered in nature. There are both cyclic and acyclic terpenes whose exact structure is determined by the specific activity of terpene synthases and the subsequent modifying enzymes. Terpene synthases are classified into two groups based on their catalytic mechanisms: class I and class II reviewed in Chen et al. (2011). In class I enzymes, the prenyldiphosphate precursor is ionized resulting in the formation of a carbocation intermediate, which undergoes cyclizations, hydride shifts and other rearrangments that result in the formation of the basic terpene skeletons of the products. Depending on these rearrangments, some class I terpene synthases specifically give rise to a single product, while others are characterized as multiproduct enzymes. This class of terpene synthases includes primarily monoterpene and sesquiterpene synthases and some diterpene synthases. Class II terpene synthases catalyze a protonation-induced cyclization of the prenyldiphosphate precursor. The reaction is terminated either via deprotanation or nucleophile capture. Characteristic enzyme of this category is the copalyldiphosphate synthase responsible for the synthesis of copalyldiphosphate, the intermediate precursor of *ent*-kaurene and several labdane-type diterpenes.

Most diterpenes are cyclic and their carbon-rings are formed by two different mechanisms. One is similar to that of class II monoterpene and sesquiterpene synthases. Examples of such reactions are those that generate macrocyclic diterpenes such as casbene and taxadiene (Dueber et al., 1978; Koepp et al., 1995). The second mechanism includes the biosynthesis of a cyclic diphosphate intermediate via class II terpene synthases, the final product being achieved through the class I terpene synthase.

Labdane-type diterpenes represent a distinct class of terpenoids with a characteristic basic bicyclic skeleton connected to an additional six-carbon chain (cyclic or acyclic) that may or may not contains an oxygen atom. Initial data on labdane-type diterpene biosynthesis came from studies on the biosynthesis of *ent*-kaurene, the diterpenoid precursor of gibberellins, and other labdane-type diterpenes that do not contain oxygen in their skeleton. *Ent*-kaurene biosynthesis involves a two-step reaction, first the cyclization of GGDP to *ent*-copalyl diphosphate (*ent*-CPP) by copalyl diphospate synthase (CPS), and then its conversion to *ent*-kaurene by *ent*-kaurene synthase (KS). Copalyl diphosphate synthase performs a protonation-initiated cyclization (class II) while *ent*-kaurene synthase performs an ionization-initiated cyclization of CPP (class I) (Sakamoto et al., 2004).

The biosynthesis of labdane-type diterpenes that function as phytoalexins in rice (*Oryza sativa*) involves *ent*-CPP or *syn*-CPP as intermediates (Otomo et al., 2004; Prisic et al., 2004; Xu et al., 2004). These phosphorylated intermediates are then further cyclized to the final diterpene products namely oryzalexins, momilactones, and phytocassanes (Nemoto et al., 2004; Otomo et al., 2004; Wilderman et al., 2004; Kanno et al., 2006). All angiosperm terpene synthases may have evolved from the gymnosperm group of bifunctional class II/I terpene synthases producing labdane-related diterpenes. Characteristic examples are: the abietadiene synthase from *Abies grandis* (Vogel et al., 1996), the levopimaradiene synthase from *Ginkgo biloba* (Schepmann et al., 2001), and the levopimaradiene/abietadiene from *Picea abies* (Martin et al., 2004). These bifunctional enzymes catalyze both reactions, first cyclizing GGDP to CPP and then converting CPP to the final tricyclic terpene, that can be further modified by additional enzymes like hydroxylases, methyltranferases, etc. (Ro et al., 2005; Hamberger and Bohlmann, 2006; Ro and Bohlmann, 2006).

The biosynthesis of oxygen-containing labdane-type diterpenes was only recently unraveled. First, it was shown that protein extracts from *Nicotiana glutinosa* and *N. tabacum* trichomes that contain labdane-type diterpenes such as abienol, labdenediol, and sclareol, could be converted to all the above oxygen-containing diterpenes *in vitro* with externally supplied GGDP (Guo et al., 1994; Guo and Wagner, 1995). This observation led to the hypothesis that their synthesis involved a copal-8-ol diphosphate intermediate, the synthesis of which is initiated by protonation of the terminal double bond of GGDP, and the formation of a bicyclic carbocation followed by capture of a hydroxyl anion (Guo and Wagner, 1995). Indeed, it was later shown that this type of enzyme exists.

**Figure 2:**
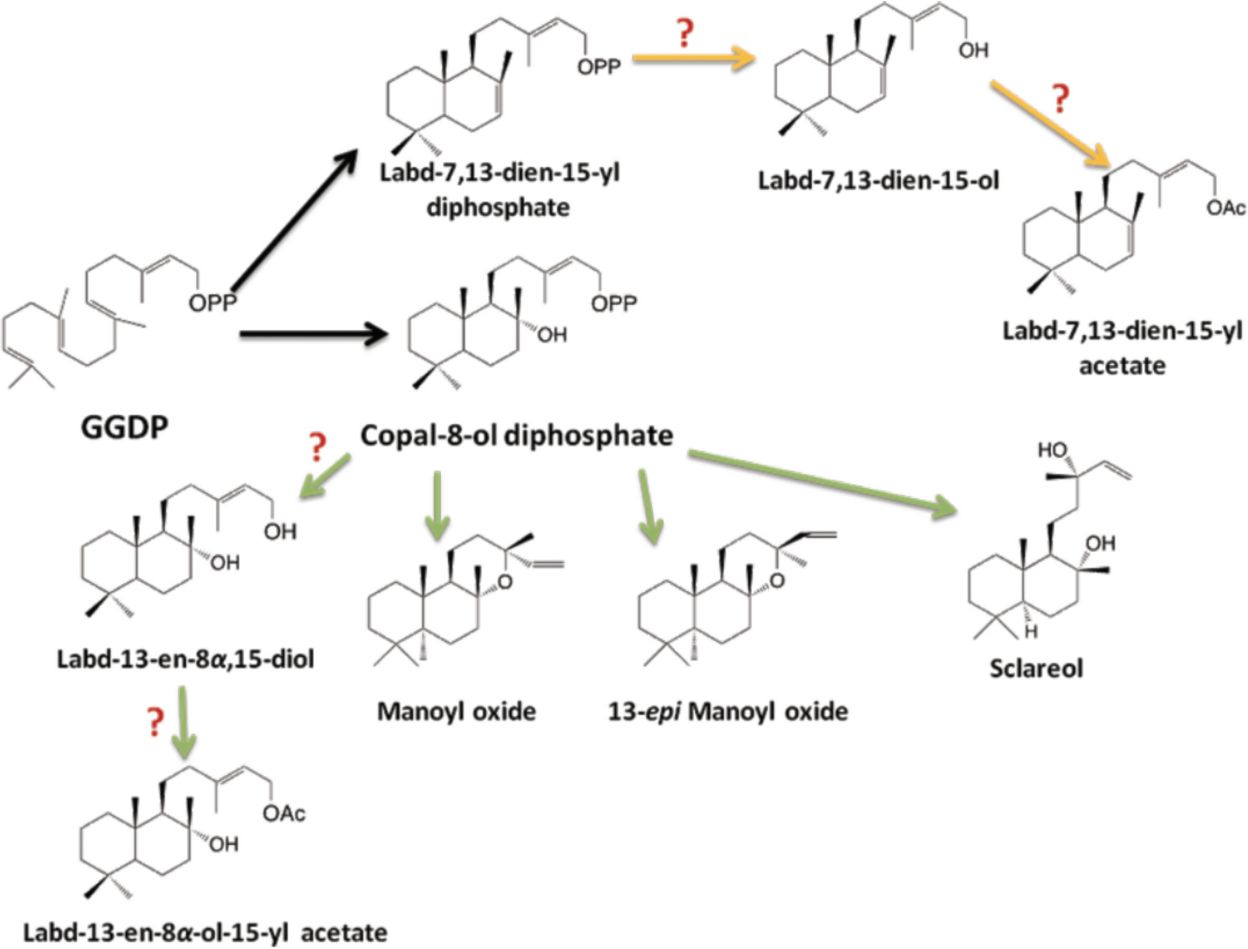
**Proposed pathway to labdane-type diterpenes predominant in *C. creticus* resin**. A protonation-initiated cyclization catalyzed by CcCLS converts GGDP to the stable bicyclic intermediate copal-8-ol diphosphate. A second ionization-initiated cyclization of copal-8-ol diphosphate results in the formation of manoyl oxide isomers, while labd-13-en-8α,15-diol could be formed either by phosphatase activity or type A diterpene synthase activity. Similarly a different phosphorylated intermediate, labd-7,13-dien-15-yl diphosphate is hypothesized to be converted to labd-7,13-dien-15-ol and then further processed to produce its derivative labd-7,13-dien-15-yl acetate. The acetylated products need the function of novel acetyltransferase(s). Black arrows indicate the biosynthetic steps that involve already characterized enzymes. Question marks indicate pathway steps that have not yet been characterized.

*C. creticus* copal-8-ol diphosphate synthase (CLS) is a type II terpene synthase expressed in the trichomes of *C. creticus*, which catalyzes the formation of the copal-8-ol diphosphate from GGDP (Falara et al., 2010) (Figure 2). Copal-8-ol diphosphate can then be cyclized to labda-13-en-8α,15-diol, labda-14-en-8,13-diol (sclareol), manoyl-oxide and 13-*epi*-manoyl-oxide. A similar pathway employing copal-8-ol diphosphate was shown to operate in the trichomes of *N. tabacum* for the biosynthesis of Z-abienol (Sallaud et al., 2012).

In *Salvia sclarea*, a class I diTPS (SsSS) has been characterized that transforms the copal-8-ol diphosphate intermediate into sclareol (Caniard et al., 2012; Schalk et al., 2012). In *Coleus forskohlii*, CfTPS2 catalyzes the synthesis of copal-8-ol diphosphate, which is then utilized by CfTPS3 to its stereospecifically form (13R)-manoyl oxide, the precursor of the highly complex labdene diterpene forskolin (Pateraki et al., 2014). A different phosphorylated intermediate, labd-7,13-dien-15-yl diphosphate, is hypothetically converted to labd-7,13-dien-15-ol and then further processed to produce its derivative labd-7,13-dien-15-yl acetate (Figure 2). In fact, we have recently functionally characterized in *E. coli* and in yeast a gene from *C. creticus* that converts GGDP to labd-7,13-dien-15-yl diphosphate (Papaefthimiou et al., 2013). Taken together with the function of CcCLS, we suggest that for each labdane-type diterpene class in *Cistus*, a unique class II synthase may be required. Recently, a bifunctional labdane-type diterpene synthase producing labd-7,13-dien-15-ol has been characterized from the gymnosperm *Selaginella moelendorfii* (Mafu et al., 2011).

### Phenylpropanoids

Phenylpropanoid compounds are abundant in the plant kingdom, either providing plants with a valuable defensive arsenal against pathogens, herbivores, and environmental stressors or facilitating the plants reproductive machinery. In addition, these molecules have important applications in the fragnance industry and in medicine. The precursor of all plant phenylpropanoids is *trans*-cinnamic acid, derived from the amino acid phenylalanine, the first enzymatic step being catalyzed by phenylalanine ammonia-lyase (PAL) leading to the synthesis of *p*-coumaroyl-CoA, the substrate of more complex aromatic phenypropanoids (Vogt, 2010). Numerous chemicals are classified as plant phenolic secondary metabolites, which are further categorized within several distinct groups based on their basic skeleton as found in simple phenols (C_6_). The more complex phenylpropanoid compounds include catechol (C_6_)*n*, phenolic acids (C_6_-C_1_), phenylacetic acids (C_6_-C_2_), coumarins (C_6_-C_3_), lignans (C_6_-C_3_)_2_, lignins (C_6_-C_3_)*n*, naphthoquinones (C_6_-C_4_), xanthones (C_6_-C_1_-C_6_), stilbenes (C_6_-C_2_-C_6_), flavonoids (anthocyanins, isoflavonoids, flavones, flavanes, proanthocyanidins, etc.) (C_6_-C_3_-C_6_) and tannins (C_6_-C_3_-C_6_)*n*. The biosynthetic pathways of several groups belonging to the very diverse phenylpropanoid compounds have been studied in several model plants, including *Arabidopsis thaliana, Meditago truncatula, Nicotiana benthamiana, Oryza sativa* (Vogt, 2010). In *Cistus*, phenolic compounds have been the target of many chemical, biological and taxonomic studies, already discussed in earlier sections of this review and summarized in Table S3. On the basis of these studies, a general pathway of the phenylrpopanoid biosynthesis in *Cistus* can be drawn (Figure S2). Several genes involved in the plant phenylpropanoid pathway have been isolated from various plant genera, while some important enzymes were further characterized and novel pathways were discovered (Cheynier et al., 2013). However, the biosynthetic pathway leading to the production of phenylpropanoids and phenolic compounds has not been yet studied in *Cistus*.

## Genomic analyses and biotechnological approaches

### Genomic analyses

*Cistus* plants are very rich sources of secondary metabolites, which make it hard to isolate quality nucleic acids in sufficient amounts. A protocol for the efficient isolation of high quality DNA and RNA from *C. creticus* subsp. *creticus* was published (Pateraki and Kanellis, 2004). This paved the way for a series of molecular studies in *C. creticus* subsp. *creticus* aiming for the elucidation of the terpenoid biosynthetic pathway. In view of the valuable properties and possible future exploitation of these natural products it was deemed necessary to study their biosynthesis and its regulation at the molecular level. In this direction, sequence based and functional genomic approaches were initiated. Initial work characterized the expression of genes coding for *CcHMGR* in the MVA pathway, *CcDXS* and *CcDXR* in the MEP pathway, and followed by the characterization of the two *CcGGDPS1* and *CcGGDPS2* (Pateraki and Kanellis, 2008). This work suggested that leaf trichomes are very active biosynthetically for terpenoids, and the pathway is regulated at the transcriptional level. Moreover, *CcHMGR* and *CcDXS* transcripts (the rate-limiting steps of the isoprenoids' pathways) increase during mechanical wounding or upon treatment with stress hormones such as JA and SA, which possibly reflects an increased need of the plant tissues for the corresponding metabolites (Pateraki and Kanellis, 2010).

The first genes isolated and functionally characterized from *C. creticus* subsp. *creticus* were *CcGGDPS1* and *2* coding for synthases of geranyl-geranyl diphosphate, the precursor of all diterpenes (Pateraki and Kanellis, 2008). Heterologous expression in *Saccharomyces cerevisiae* revealed that these full-length cDNAs possessed GGDPS enzyme activity. Gene and protein expression investigations proposed that this enzyme is developmentally and tissue-regulated showing maximum expression in trichomes and smallest leaves (0.5–1.0 cm).

Next, in order to search for putative terpene synthases, an EST library was built using RNA extracted from trichomes isolated from young leaves (Falara et al., 2008). This was decided on the basis of chemical profiling which showed that young leaf trichomes were richer in labdane-type diterpenes compared to mature ones. The subsequent EST analysis that was conducted produced 2022 clones (Falara et al., 2008; http://www.ests-pharm.web.auth.gr/ests.php). Functional annotation of the 2022 expressed sequence tags (ESTs) from the trichome cDNA library, based on homology to *A. thaliana* genes, showed that 8% of the putative identified sequences belonged to secondary metabolism pathways mainly in flavonoid and terpenoid biosynthesis. Custom DNA microarrays assembled with 1248 individual clones from the cDNA library enabled transcriptome comparisons between trichomes and trichome-free tissues. Verification of the DNA microarrays data by RT-PCR pinpointed a germacrene B synthase (*CcGerB*) as a trichome specific gene (Falara et al., 2008). Further, the isolation of the promoter of this gene was achieved (Saramourtsi, 2013).

The full-length cDNA of copal-8-ol diphosphate diterpene synthase (*CcCLS)* was functionally characterized from *C. creticus*, elucidating the novel first step in the labdane-type diterpenes biosynthetic pathway not only in *Cistus* but in all angiosperms (Falara et al., 2010, Figure 2). Gene expression analysis revealed that *CcCLS* is preferentially expressed in trichomes, with higher transcript levels measured in the glandular trichomes of young leaves compared to fully expanded leaves. Interestingly, *CcCLS* transcript levels increased after mechanical wounding used to simulate herbivore attack. Chemical analyses revealed that labdane-type diterpene production in general followed a similar pattern, with higher concentrations in trichomes of young leaves and increased accumulation upon wounding, indicating that increased diterpene biosynthesis is related to the plant's defense mechanisms. Application of New Generation Sequencing (NGS) in *C. creticus* trichomes RNA resulted in a total of 385,143 contig sequences (114,239 unigenes) with a mean length of 207 nucleotides. Among those, 2000 unigenes were related to the biosynthesis, transport and catabolism of secondary metabolites. Twenty partial sequences were phylogenetically related to putative diterpene synthases (Papaefthimiou et al., 2013). Among those, two diterpene synthases have already been functionally characterized (see Biosynthetic Pathway for Terpenes Including Labdane-Type Diterpenes).

Another protein isolated and characterized from *C. creticus* was the key transcriptional regulator *TRANSPARENT TESTA GLABRA1* (*CcTTG1*) (Ioannidi, 2009). In *Arabidopsis*, TTG1 is involved in trichome differentiation, in the regulation of flavonoids and in seed mucilage production (Walker et al., 1999). The glabrous phenotype of the loss of function *A. thaliana ttg1* mutants was restored upon transformation with CcTTG1. The gene was also able to restore the seed coat pigmentation defect of the mutant. The yeast two-hybrid screen resulted in the identification of seven CcTTG1 interactors. Four of them were further analyzed using the yeast-two-hybrid system. Three protein-protein interactions were tested *in planta*, using a transient expression system in tobacco epidermal cells. The analysis was based on the Bi-molecular Fluorescent Complementation (BiFC) and verified the interaction of CcTTG1 with two Squamosa promoter Binding Proteins (SBP). Also the interactions were detected in the nucleus of the tobacco epidermal cells. This work reported the first protein-protein interaction of the SBP family of transcription factors and a novel interaction of the CcTTG1 protein (Ioannidi, 2009).

Other genetic data available for *Cistus* is restricted to genes used as molecular markers in taxonomic and phylogenetic studies. Several studies include the isolation and use of partial sequences from a variety of commonly used molecular markers in order to achieve the delimitation of *Cistus* species. These include the nuclear (*ncp*GS, *ITS*) and plastid (*trnL-trnF, trnK-matK, trnS-trnG, rbcL*) DNA sequences, the *trn-F and RPL32-TRNL* sequences of *cpDNA* (Falchi et al., 2009) RNA polymerase subunits (Pawluczyk et al., 2012), as well as genetic markers (*ISSR*—PCR amplification) (Paolini et al., 2009).

### *In vitro* cultivation of Cistus

The first *in vitro* cultivation of *Cistus* was reported in 1991 (M'Kada et al., 1991) on stem nodal segments for the multiplication of *Cistus* X *purpureus* Lam, using MS medium (Murashige and Skoog, 1962) supplemented with cytokinins. Later, a protocol was established for *in vitro* propagation of *Helianthemum almeriense* (Cistaceae), using MS with plant growth elicitors for shoot generation (Morte and Honrubia, 1992). Soon after, an improved protocol for *in vitro* propagation was established with high concentrations of growth elicitors in MS medium leading to successful propagation of six rock-rose species (*C. albidus* L., *C. clusii* Dunal, *C. ladanifer* L., *C. laurifolius* L., *C. psilosepalus* L., and *C. salviifolius* L) (Iriondo et al., 1995). *In vitro* propagation of *C. creticus* was successfully established by three separate approaches. In the first approach, a combination of growth elicitors was used on shoots proliferation and on callus regeneration (Pela et al., 2000). In the second approach, shoot formation was obtained 30 days after the first subculture in WPM. The segments were rooted, after supplementing the medium with IBA (0.98–3.94 μM) or NAA (0.1–0.5 μM), and zeatin (0.2–0.5 mg l-1) was used for callus induction (Zygomala et al., 2003). In the third and most recent work, studying the micropropagation of *C. creticus* (Madesis et al., 2011), rapid proliferation of shoot-tips was achieved using MS, supplemented with growth elicitors, and after 4 weeks, shoots were transferred to MS for rooting or further development. In order to achieve rooting, shoots longer than 1 cm were used and cultured on MS without growth regulators. *In vitro* proliferation of *Cistus* has also been studied in *C. clusii*, with satisfactory results (Ruta and Morone-Fortunato, 2010). In conclusion, methodologies are in place for *in vitro* cultivation *of Cistus* plants.

### Conflict of interest statement

The authors declare that the research was conducted in the absence of any commercial or financial relationships that could be construed as a potential conflict of interest.
