# Supplementary material for: Genus Cistus: a model for exploring labdane-type diterpenes' biosynthesis and a natural source of high value products with biological, aromatic, and pharmacological properties
Source: Front Chem. 2014 Jun 11;2:35. doi: 10.3389/fchem.2014.00035 (PMC4052220; doi:10.3389/fchem.2014.00035)
Supplement: Supplementary file 1 [file DataSheet1.ZIP › Supp Table 2.PDF]

Table S2. The specific botanical characteristics of the main 10 species of genus *Cistus*: color of the flower petal, purple (P) or white (W); number of sepals (3 or 5); number of fruit compartments containing seeds (5 or 6-12); type of leaf base, petiolate (P) or sessile (S) and size of the style (XS, S, M, L) compared with flower stamens (Modified from Guzmán and Vargas, 2005).

		Main Mediterranean species	Petal color **P**urple or **W**hite	Number of sepals	Number of fruit com-partments	Leaf base **P**etiolate or **S**essile	Style size **XS**, **S**, **M**, **L** (compared with stamens)
**Subg. I: *Cistus* D.**	**Sec. 1: *Erythrocistus***	*C. albidus* L.	P	5	5	S	M
		C. creticus L.	P	5	5	P	M
		C. crispus L.	P	5	5	S	M
		C. parviflorus Lam.	P	5	5	P	XS
**Subg. II: *Leucocistus* Willk**	**Sec. 1: *Ledonia***	*C. monspeliensis* L.	W	5	5	S	S
		C. populifolius L.	W	5	5	P	XS
		C. salviifolius L.	W	5	5	P	S
	**Sec. 2: *Ladanium***	*C. ladanifer* L.	W	3	6-12	S	XS
		C. laurifolius L.	W	3	5	P	XS
**Subg. III:**	***Halimioides***	*C. clusii* Dunal	W	3	5	S	S
