# Supplementary material for: Genus Cistus: a model for exploring labdane-type diterpenes' biosynthesis and a natural source of high value products with biological, aromatic, and pharmacological properties
Source: Front Chem. 2014 Jun 11;2:35. doi: 10.3389/fchem.2014.00035 (PMC4052220; doi:10.3389/fchem.2014.00035)
Supplement: Supplementary file 1 [file DataSheet1.ZIP › Supp Table 3.PDF]

Supplementary Table S3. Total number of secondary metabolites detected in the 10 *Cistus* species covered in this Review

	C. albidus	*C. creticus* subsp. *creticus*	*C. creticus* subsp. *eriocephalus*	*C. clusii*	*C. crispus*	*C. ladanifer*	*C. laurifolius*	*C. monspeliensis*	*C. parviflorus*	*C. populifolius*	*C. salviifolius*	**All species**
**TERPENES**	**140**	**92**	**47**	**1**	**-**	**72**	**4**	**107**	**99**	**10**	**160**	**397**
Monoterpenes	**34**	**36**	**17**	**-**	**-**	**47**	**-**	**22**	**17**	**-**	**32**	**101**
Sesquiterpenes	**101**	**35**	**19**	**-**	**-**	**18**	**-**	**33**	**44**	**-**	**85**	**178**
Diterpenes	**5**	**21**	**11**	**1**	**-**	**7**	**4**	**52**	**38**	**10**	**43**	**118**
Other Diterpenes	**3**	**1**	**-**	**-**	**-**	**-**	**-**	**13**	**21**	**-**	**25**	**36**
Labdane-type diterpenes	**2**	**20**	**11**	**1**	**-**	**7**	**1**	**17**	**17**	**-**	**18**	**47**
Clerodane Diterpenes	**-**	**-**	**-**	**-**	**-**	**-**	**3**	**22**	**-**	**10**	**-**	**35**
**PHENYLPROPANOIDS**	**24**	**12**	**-**	**23**	**10**	**43**	**58**	**17**	**19**	**10**	**58**	**162**
Flavonoids	**18**	**12**	**-**	**15**	**6**	**27**	**44**	**6**	**17**	**1**	**39**	**128**
Phenolic compounds	**2**	**-**	**-**	**3**	**2**	**3**	**6**	**6**	**2**	**1**	**8**	**17**
Tannins	**4**	**-**	**-**	**5**	**2**	**12**	**7**	**4**	**-**	**8**	**9**	**12**
**HYDROCARBONS**	**9**	**-**	**-**	**-**	**-**	**-**	**-**	**20**	**8**	**-**	**14**	**24**
**FATTY ACIDS**	**24**	**-**	**-**	**-**	**-**	**-**	**-**	**17**	**5**	**-**	**5**	**35**
**CARBONYLIC COMPOUNDS**	**7**	**-**	**2**	**-**	**-**	**6**	**1**	**10**	**7**	**-**	**22**	**36**
**PHYTOHORMONES & VITAMINS**	**18**	**-**	**-**	**-**	**-**	**-**	**-**	**-**	**-**	**-**	**-**	**18**
**OTHERS**	**11**	**-**	**3**	**2**	**-**	**11**	**11**	**2**	**11**	**-**	**29**	**61**
