# Supplementary material for: Genus Cistus: a model for exploring labdane-type diterpenes' biosynthesis and a natural source of high value products with biological, aromatic, and pharmacological properties
Source: Front Chem. 2014 Jun 11;2:35. doi: 10.3389/fchem.2014.00035 (PMC4052220; doi:10.3389/fchem.2014.00035)
Supplement: Supplementary file 1 [file DataSheet1.ZIP › Supp Table 4.PDF]

Supplementary Table S4. Chemical composition of aerial parts and essential oils of *Cistus* species

	C. albidus	***C. creticus* subsp. *creticus***	***C. creticus* subsp. *eriocephalus***	***C. clusii***	***C. crispus***	***C. ladanifer***	***C. laurifolius***	***C. monspeliensis***	***C. parviflorus***	***C. populifolius***	***C. salviifolius***
**TERPENES**											
Monoterpenes											
**(*E*)-Ocimene**	(Maccioni et al., 2007)										
**(*E*)-Ocimenone**						(Gomes et al., 2005)					
**(*Z*)-Ocimene**	(Maccioni et al., 2007)										
**(*Z*)-Ocimenone**						(Gomes et al., 2005)					
**1,8-Cineole (Eucalyptol)**	(Ormeño et al., 2007)	(Demetzos et al., 1995)				(Robles et al., 2003)		(Rivoal et al., 2010; Loizzo et al., 2013; Jemia et al., 2013)			
**2,3,3-Trimethyl-bicyclo[2.2.1]-heptan-2-ol**		(Demetzos et al., 1994b)									
**3-Carene**	(Ormeño et al., 2007)					(Ramalho et al., 1999)		(Rivoal et al., 2010)			
**3-Methyl camphenilanol**			(Demetzos et al., 1997)								
**3-Phenylpropionic acid**		(Demetzos et al., 1995)									
**4-*δ*-Carene**											(Demetzos et al., 2002a)
**Borneol**	(Ormeño et al., 2007)	(Demetzos et al., 1994b; Demetzos et al., 1995)	(Demetzos et al., 1997)			(Mariotti et al., 1997; Ramalho et al., 1999; Robles et al., 2003; Gomes et al., 2005; Teixeira et al., 2007; Zidane et al., 2013)		(Rivoal et al., 2010;Loizzo et al., 2013)	(Demetzos et al., 1990b)		(Loizzo et al., 2013)
**Bornyl acetate**		(Demetzos et al., 1994b)	(Demetzos et al., 1997)			(Mariotti et al., 1997; Gomes et al., 2005; Teixeira et al., 2007)		(Rivoal et al., 2010;Loizzo et al., 2013)	(Demetzos et al., 1990b)		
**Camphene**	(Ormeño et al., 2007)	(Demetzos et al., 1994b; Demetzos et al., 1995)				(Mariotti et al., 1997; Robles et al., 2003; Gomes et al., 2005; Zidane et al., 2013)		(Rivoal et al., 2010;Loizzo et al., 2013)			

	C. albidus	*C. creticus* subsp. *creticus*	*C. creticus* subsp. *eriocephalus*	*C. clusii*	*C. crispus*	*C. ladanifer*	*C. laurifolius*	*C. monspeliensis*	*C. parviflorus*	*C. populifolius*	*C. salviifolius*
**Camphor**	(Ormeño et al., 2007; Llusià et al., 2010)	(Demetzos et al., 1994b)	(Demetzos et al., 1997)			(Ramalho et al., 1999; Zidane et al., 2013)		(Rivoal et al., 2010)	(Angelopoulou et al., 2001b)		(Demetzos et al., 2002a; Loizzo et al., 2013)
**Carvacrol**	(Maccioni et al., 2007)	(Demetzos et al., 1995)						(Angelopoulou et al., 2001a; Angelopoulou et al., 2002; Paolini et al., 2009; Jemia et al., 2013)	(Angelopoulou et al., 2001b)		(Demetzos et al., 2002a; Loizzo et al., 2013)
**Carveol**						(Robles et al., 2003)					
**Carvone**						(Mariotti et al., 1997)					
**Chrysanthenone**											(Loizzo et al., 2013)
**Cinnamyl alcohol**		(Demetzos et al., 1995)									
***cis*-Carveol**			(Demetzos et al., 1997)								
***cis*-Linalool oxide**	(Ormeño et al., 2007)										
***cis*-Rose oxide**						(Robles et al., 2003)					
***cis*-Sabinene hydrate**	(Maccioni et al., 2007)							(Loizzo et al., 2013)			
***cis*-Thujone**	(Ormeño et al., 2007)										
***cis*-Verbenol**						(Ramalho et al., 1999)					
***cis*-*α*-Ambrinol**									(Angelopoulou et al., 2001b)		(Demetzos et al., 2002a)
***cis*-*β*-Terpineol**											(Demetzos et al., 2002a)
**Citronellol**						(Teixeira et al., 2007)					
**Dehydro-*p*-cymene**		(Demetzos et al., 1994b)									
**Endoborneol**											(Demetzos et al., 2002a)
**Eugenol**	(Paolini et al., 2008)					(Ramalho et al., 1999; Teixeira et al., 2007)		(Loizzo et al., 2013)	(Angelopoulou et al., 2001b)		(Demetzos et al., 2002a; Loizzo et al., 2013)
***exo*-Methyl-camphenilol**						(Zidane et al., 2013)					
**Fenchol**			(Demetzos et al., 1997)								

	C. albidus	*C. creticus* subsp. *creticus*	*C. creticus* subsp. *eriocephalus*	*C. clusii*	*C. crispus*	*C. ladanifer*	*C. laurifolius*	*C. monspeliensis*	*C. parviflorus*	*C. populifolius*	*C. salviifolius*
**Geraniol**						(Teixeira et al., 2007)					
**Geranyl acetate**						(Mariotti et al., 1997; Teixeira et al., 2007)					
**Geranyl acetone**									(Angelopoulou et al., 2001b)		(Demetzos et al., 2002a)
**I-Bornyl acetate**						(Zidane et al., 2013)					
**Isoborneol**		(Demetzos et al., 1994b)	(Demetzos et al., 1997)			(Mariotti et al., 1997)					
**iso-neo Carvomenthyl acetate**											(Demetzos et al., 2002a)
***iso-neo* Menthol**									(Angelopoulou et al., 2001b)		(Demetzos et al., 2002a)
**Isopinocamphone**						(Mariotti et al., 1997)					
**Isopulegyl acetate**											(Demetzos et al., 2002a)
**Limonene**	(Maccioni et al., 2007; Ormeño et al., 2007; Llusià et al., 2010)	(Demetzos et al., 1994b)	(Paolini et al., 2009)			(Mariotti et al., 1997; Robles et al., 2003; Gomes et al., 2005)		(Rivoal et al., 2010; Jemia et al., 2013)			(Loizzo et al., 2013)
**Linalool**		(Demetzos et al., 1994b)	(Demetzos et al., 1997)			(Teixeira et al., 2007)					(Demetzos et al., 2002a; Loizzo et al., 2013)
**Linalyl acetate**								(Jemia et al., 2013)			
**Menthol**											(Demetzos et al., 2002a)
**Methyl chavicol**											(Demetzos et al., 2002a)
**Methyl eugenol**									(Angelopoulou et al., 2001b)		(Demetzos et al., 2002a)
**Myrcene**	(Maccioni et al., 2007)		(Paolini et al., 2009)								
**Myrtenal**						(Mariotti et al., 1997)					(Demetzos et al., 2002a)
**Myrtenol**		(Demetzos et al., 1995)	(Demetzos et al., 1997)			(Mariotti et al., 1997; Robles et al., 2003; Gomes et al., 2005)					(Loizzo et al., 2013)
**Myrtenyl acetate**						(Mariotti et al., 1997)					

	C. albidus	*C. creticus* subsp. *creticus*	*C. creticus* subsp. *eriocephalus*	*C. clusii*	*C. crispus*	*C. ladanifer*	*C. laurifolius*	*C. monspeliensis*	*C. parviflorus*	*C. populifolius*	*C. salviifolius*
***neo*-Menthol**											(Demetzos et al., 2002a)
**Neral**											(Demetzos et al., 2002a)
**Neryl acetate**	(Paolini et al., 2008)										
**Neryl acetone**									(Angelopoulou et al., 2001b)		(Demetzos et al., 2002a)
***ortho*-Guaiacol**											(Demetzos et al., 2002a)
***para*-Methyl anisol**											(Demetzos et al., 2002a)
***p*-Cymen-8-ol**		(Demetzos et al., 1995)				(Robles et al., 2003; Zidane et al., 2013)					
***p*-Cymene**	(Maccioni et al., 2007; Ormeño et al., 2007)	(Demetzos et al., 1994b; Demetzos et al., 1995)				(Mariotti et al., 1997; Robles et al., 2003; Gomes et al., 2005; Zidane et al., 2013)		(Jemia et al., 2013)			
***p*-Cymenene**						(Gomes et al., 2005)					
**Perilla alcohol**		(Demetzos et al., 1994b)									
**Pinocarvone**						(Mariotti et al., 1997; Gomes et al., 2005)					
**Piperitone**	(Maccioni et al., 2007)										
***p*-Mentha-1,4-dien-7-ol**						(Zidane et al., 2013)					
**Sabinene**	(Maccioni et al., 2007; Ormeño et al., 2007)					(Mariotti et al., 1997; Zidane et al., 2013)					
**Safranal**	(Paolini et al., 2008)							(Loizzo et al., 2013)			
**Terpinen-4-ol**	(Maccioni et al., 2007; Ormeño et al., 2007; Paolini et al., 2008)	(Demetzos et al., 1994b; Demetzos et al., 1995)	(Demetzos et al., 1997)			(Mariotti et al., 1997; Robles et al., 2003; Gomes et al., 2005; Zidane et al., 2013)		(Loizzo et al., 2013)	(Angelopoulou et al., 2001b)		(Demetzos et al., 2002a; Loizzo et al., 2013)
**Terpineol**									(Demetzos et al., 1990b)		
**Terpinolene**		(Demetzos et al., 1994b)				(Mariotti et al., 1997; (Zidane et al., 2013)					

	C. albidus	*C. creticus* subsp. *creticus*	*C. creticus* subsp. *eriocephalus*	*C. clusii*	*C. crispus*	*C. ladanifer*	*C. laurifolius*	*C. monspeliensis*	*C. parviflorus*	*C. populifolius*	*C. salviifolius*
**Thujol**		(Demetzos et al., 1995)									
**Thymol**	(Maccioni et al., 2007)	(Demetzos et al., 1994b; Demetzos et al., 1995)							(Demetzos et al., 1990b; Angelopoulou et al., 2001b)		(Demetzos et al., 2002a)
***trans*-Carveol**		(Demetzos et al., 1995)				(Mariotti et al., 1997)					
***trans*-Isopinocarveol**			(Demetzos et al., 1997)								
***trans*-Perillyl alcohol**									(Demetzos et al., 1990b)		
***trans*-Pinocarveol**						(Mariotti et al., 1997; Robless et al., 2003; Gomes et al., 2005)					
***trans-p*-Menth-2-en-1-ol**											(Loizzo et al., 2013)
***trans-p*-Menth-2-en-7-ol**		(Demetzos et al., 1995)									
***trans-α*-Ambrinol**									(Angelopoulou et al., 2001b)		(Demetzos et al., 2002a)
**Tricyclene**	(Ormeño et al., 2007)					(Mariotti et al., 1997; Robless et al., 2003; Gomes et al., 2005)					
**Valeric acid**		(Demetzos et al., 1995)									
**Verbenone**	(Ormeño et al., 2007)	(Demetzos et al., 1995)	(Demetzos et al., 1997)			(Mariotti et al., 1997; Ramalho et al., 1999; Robles et al., 2003; Zidane et al., 2013)					
***α*-Campholenic aldehyde**						(Mariotti et al., 1997; Robles et al., 2003)					
***α*-Fenchene**		(Demetzos et al., 1994b)									
***α*-Fenchyl alcohol**		(Demetzos et al., 1994b)									

	C. albidus	*C. creticus* subsp. *creticus*	*C. creticus* subsp. *eriocephalus*	*C. clusii*	*C. crispus*	*C. ladanifer*	*C. laurifolius*	*C. monspeliensis*	*C. parviflorus*	*C. populifolius*	*C. salviifolius*
***α*-Phellandrene**	(Maccioni et al., 2007; Ormeño et al., 2007)	(Demetzos et al., 1994b)				(Ramalho et al., 1999)		(Rivoal et al., 2010)			
***α*-Pinene**	(Maccioni et al., 2007; Ormeño et al., 2007; Llusià et al., 2010)	(Demetzos et al., 1994b; Demetzos et al., 1995)	(Paolini et al., 2009)			(Mariotti et al., 1997; Robles et al., 2003; Gomes et al., 2005; Teixeira et al., 2007; Zidane et al., 2013)		(Rivoal et al., 2010;Loizzo et al., 2013)			
***α*-Terpinene**	(Maccioni et al., 2007; Ormeño et al., 2007)	(Demetzos et al., 1994b)				(Mariotti et al., 1997; Robles et al., 2003; Zidane et al., 2013)		(Rivoal et al., 2010)			
***α*-Terpineol**	(Maccioni et al., 2007; Paolini et al., 2008)	(Demetzos et al., 1994b; Demetzos et al., 1995)	(Demetzos et al., 1997)			(Mariotti et al., 1997; Teixeira et al., 2007; Zidane et al., 2013)		(Angelopoulou et al., 2002; Rivoal et al., 2010)	(Angelopoulou et al., 2001b)		(Demetzos et al., 2002a; Loizzo et al., 2013)
***α*-Terpinyl acetate**		(Demetzos et al., 1995)									
***α*-Thujene**	(Ormeño et al., 2007)	(Demetzos et al., 1994b)				(Zidane et al., 2013)					
***β*-Cyclocitral**	(Paolini et al., 2008)							(Loizzo et al., 2013)			(Loizzo et al., 2013)
***β*-Cymene**								(Rivoal et al., 2010)			
***β*-Myrcene**	(Ormeño et al., 2007)							(Rivoal et al., 2010)			
***β*-Phellandrene**	(Maccioni et al., 2007; Ormeño et al., 2007)					(Mariotti et al., 1997)					
***β*-Pinene**	(Ormeño et al., 2007; Llusià et al., 2010)	(Demetzos et al., 1994b)				(Mariotti et al., 1997; Robles et al., 2003; Zidane et al., 2013)		(Rivoal et al., 2010;Loizzo et al., 2013);			
***β*-Thujaplicinol**									(Angelopoulou et al., 2001b)		(Demetzos et al., 2002a)
***γ*-Terpinene**	(Maccioni et al., 2007; Ormeño et al., 2007)	(Demetzos et al., 1994b)	(Paolini et al., 2009)			(Mariotti et al., 1997; Robles et al., 2003; Zidane et al., 2013)					
***γ*-Terpineol**		(Demetzos et al., 1995)									

	C. albidus	*C. creticus* subsp. *creticus*	*C. creticus* subsp. *eriocephalus*	*C. clusii*	*C. crispus*	*C. ladanifer*	*C. laurifolius*	*C. monspeliensis*	*C. parviflorus*	*C. populifolius*	*C. salviifolius*
***δ*-Terpinene**	(Ormeño et al., 2007)										
***δ*-Verbenone**						(Gomes et al., 2005)					
Sesquiterpenes											
**(-)-*α*-Cedrene**	(Llusià et al., 2010)										
**(*E*)-Caryophyllene**									(Angelopoulou et al., 2001b)		(Demetzos et al., 2002a)
**(*E*)-Nerolidol**											(Loizzo et al., 2013)
**(*E*)-Nuciferol**	(Paolini et al., 2008)										(Loizzo et al., 2013)
**(*E*)-*α*-Atlantone**											(Loizzo et al., 2013)
**(*E*)-*β*-Caryophyllene**	(Paolini et al., 2008)										
**(*E*)-*β*-Farnesene**						(Mariotti et al., 1997)					(Loizzo et al., 2013)
**(*E, E*)-Farnesyl acetate**									(Angelopoulou et al., 2001b)		(Demetzos et al., 2002a)
**(*E,Z*)-Farnesol**											(Loizzo et al., 2013)
**(*Z*)-Caryophyllene**											(Demetzos et al., 2002a)
**(*Z*)-Nerolidol**											(Loizzo et al., 2013)
**(*Z*)-*α*-*trans*-Bergamotol**								(Loizzo et al., 2013)			
**1-(2,3,6-trimethylphenyl)-3-Buten-2-one**		(Demetzos et al., 1994b)									
**1,10-di-*epi*-Cubenol**	(Maccioni et al., 2007; Paolini et al., 2008)								(Angelopoulou et al., 2001b)		(Demetzos et al., 2002a)
**1,5,5-Trimethyl-9-oxa-bicyclo[4,3,0]-non-2-ene**								(Robles and Garzino, 2000)			
**1,5-di-*epi*-Bourbonene**	(Paolini et al., 2008)										
**14(*E*)–Hydroxy-9-*epi*-caryophyllene**									(Angelopoulou et al., 2001b)		(Demetzos et al., 2002a)

	C. albidus	*C. creticus* subsp. *creticus*	*C. creticus* subsp. *eriocephalus*	*C. clusii*	*C. crispus*	*C. ladanifer*	*C. laurifolius*	*C. monspeliensis*	*C. parviflorus*	*C. populifolius*	*C. salviifolius*
**14-Hydroxy-*β*-caryophyllene**											(Loizzo et al., 2013)
**1-*epi*-Cubenol**	(Maccioni et al., 2007; Paolini et al., 2008)								(Angelopoulou et al., 2001b)		(Demetzos et al., 2002a)
**1H-3*α*,7-Methanoazulene-octahydro[1,9,9]-trimethyl**									(Demetzos et al., 1990b)		
**4-*epi*-Cubebol**	(Paolini et al., 2008)										
**5-*neo*-Cedranol**											(Demetzos et al., 2002a)
**6,10,11,11-Tetramethyl-tricyclo-[5.3.0.1-(2,3)]-undec-7-ene**		(Demetzos et al., 1999)									
**6,7-Dihydrofarnesol**		(Demetzos et al., 1995)									
**6-*epi*-Shyobunone**	(Paolini et al., 2008)										
**7-*epi*-*α*-Eudesmol**									(Angelopoulou et al., 2001b)		(Demetzos et al., 2002a)
**7-*epi*-*β*-Eudesmol**								(Angelopoulou et al., 2001a)			
**9-*epi*-(*E*)-Caryophyllene**											(Demetzos et al., 2002a)
***α*-Caryophyllene**	(Ormeño et al., 2007; Llusià et al., 2010)										
***α*-Corocalene**	(Paolini et al., 2008)										
***allo*-Aromadendrene**	(Maccioni et al., 2007; Ormeño et al., 2007; Paolini et al., 2008)	(Demetzos et al., 1994b)				(Mariotti et al., 1997; Gomes et al., 2005)			(Angelopoulou et al., 2001b)		(Demetzos et al., 2002a)
***allo*-Aromadendrene epoxide**	(Paolini et al., 2008)										

	C. albidus	*C. creticus* subsp. *creticus*	*C. creticus* subsp. *eriocephalus*	*C. clusii*	*C. crispus*	*C. ladanifer*	*C. laurifolius*	*C. monspeliensis*	*C. parviflorus*	*C. populifolius*	*C. salviifolius*
***α*-Quaiene**		(Demetzos et al., 1994b; Demetzos et al., 1995)									
***ar*-Curcumen-15-al**	(Paolini et al., 2008)										
***ar*-Curcumene**	(Maccioni et al., 2007; Paolini et al., 2008)										(Loizzo et al., 2013)
**Aromadendrene**	(Paolini et al., 2008; Llusià et al., 2010)	(Demetzos et al., 1995)	(Demetzos et al., 1997)								
**Arteannuic alcohol**											(Demetzos et al., 2002a)
**Bicyclogermacrene**	(Paolini et al., 2008)										
**Bisabola-2,10-diene-[1-9]-oxide**	(Paolini et al., 2008)										
**Bulnesol**	(Maccioni et al., 2007)		(Demetzos et al., 1997)								
**Cadalene**	(Paolini et al., 2008)		(Demetzos et al., 1997)						(Angelopoulou et al., 2001b)		(Demetzos et al., 2002a)
**Cadina-1,4-diene**	(Paolini et al., 2008)							(Angelopoulou et al., 2002)			(Demetzos et al., 2002a)
**Calacorene**		(Demetzos et al., 1994b; Demetzos et al., 1995)							(Demetzos et al., 1990b)		
**Calamene**									(Demetzos et al., 1990b)		
**Calamenene**	(Paolini et al., 2008)	(Demetzos et al., 1995)	(Demetzos et al., 1997)								
**Caryophylla-3,8(13)-dien-5-*β*-ol**								(Loizzo et al., 2013)			(Loizzo et al., 2013)
**Caryophylla-4(12),8(13)-dien-5-ol**								(Angelopoulou et al., 2002)			
**Caryophylladienol I**	(Paolini et al., 2008)										
**Caryophylladienol II**	(Paolini et al., 2008)										(Loizzo et al., 2013)
**Caryophyllene**									(Demetzos et al., 1990b)		

	C. albidus	C. creticus subsp. creticus	C. creticus subsp. eriocephalus	C. clusii	C. crispus	C. ladanifer	C. laurifolius	C. monspeliensis	C. parviflorus	C. populifolius	C. salviifolius
**Caryophyllene alcohol**											(Demetzos et al., 2002a)
**Caryophyllene oxide**	(Maccioni et al., 2007; Ormeño et al., 2007; Paolini et al., 2008)	(Demetzos et al., 1994b)				(Mariotti et al., 1997; Gomes et al., 2005)		(Angelopoulou et al., 2001a; Angelopoulou et al., 2002)	(Angelopoulou et al., 2001b)		(Demetzos et al., 2002a; Loizzo et al., 2013)
**Caryophyllenol II**	(Paolini et al., 2008)										(Loizzo et al., 2013)
**Cedr-8(15)en-9-yl-acetate**									(Angelopoulou et al., 2001b)		(Demetzos et al., 2002a)
**Cedr-8(15)-en-9*α*-ol**									(Angelopoulou et al., 2001b)		(Demetzos et al., 2002a)
**Cedryl acetate**	(Maccioni et al., 2007)										
***cis*-14-Muurol-5-en-4-one**											(Demetzos et al., 2002a)
***cis*-Calamenene**											(Demetzos et al., 2002a)
***cis*-Muurola-4(14),5-diene**	(Maccioni et al., 2007)								(Angelopoulou et al., 2001b)		(Demetzos et al., 2002a)
***cis*-*α*-Bergamotene**	(Maccioni et al., 2007; Paolini et al., 2008)										
***cis*-*α*-Copaen-8-ol**											(Loizzo et al., 2013)
***cis*-*γ*-Cadinene**	(Maccioni et al., 2007)										
**Copaene**	(Ormeño et al., 2007; Llusià et al., 2010)										
**Cubebol**	(Paolini et al., 2008)										
**Cubenol**	(Paolini et al., 2008)								(Angelopoulou et al., 2001b)		(Demetzos et al., 2002a; Loizzo et al., 2013)
**Curcuphenol**	(Paolini et al., 2008)										
**Cyclosativene**	(Maccioni et al., 2007)					(Mariotti et al., 1997; Zidane et al., 2013)					(Demetzos et al., 2002a)

	C. albidus	*C. creticus* subsp. *creticus*	*C. creticus* subsp. *eriocephalus*	*C. clusii*	*C. crispus*	*C. ladanifer*	*C. laurifolius*	*C. monspeliensis*	*C. parviflorus*	*C. populifolius*	*C. salviifolius*
**Dehydrosesquicineole**	(Paolini et al., 2008)										
**Drima-7,9(11)-diene**		(Demetzos et al., 1999)						(Angelopoulou et al., 2001a;Angelopoulou et al., 2002; Loizzo et al., 2013)			(Demetzos et al., 2002a)
**Drimane sesquiterpene**		(Demetzos et al., 1994b)-									
**Drimenol**											(Demetzos et al., 2002a)
**Elemol**	(Maccioni et al., 2007)							(Angelopoulou et al., 2001a)	(Angelopoulou et al., 2001b)		(Demetzos et al., 2002a)
***epi*-10-*γ*-Eudesmol**	(Maccioni et al., 2007)										
***epi*-Cedrol**									(Angelopoulou et al., 2001b)		(Demetzos et al., 2002a)
***epi*-Cubenol**								(Angelopoulou et al., 2002)			
***epi*-*α*-Bisabolol**	(Maccioni et al., 2007; Paolini et al., 2008)										
***epi*-*β*-Cadinol**								(Angelopoulou et al., 2001a)			
**Eremophilene**			(Demetzos et al., 1997)								
**Eudesma-4(15),7-dien-1*β*-ol**	(Paolini et al., 2008)										
**Eugenyl acetate**									(Demetzos et al., 1990b)		(Demetzos et al., 2002a)
**Farnesol**		(Demetzos et al., 1995)									
**Germacra-1(10),4-dien-6*β*-ol**	(Paolini et al., 2008)										
**Germacrene B**	(Llusià et al., 2010)										(Loizzo et al., 2013)

	C. albidus	*C. creticus* subsp. *creticus*	*C. creticus* subsp. *eriocephalus*	*C. clusii*	*C. crispus*	*C. ladanifer*	*C. laurifolius*	*C. monspeliensis*	*C. parviflorus*	*C. populifolius*	*C. salviifolius*
**Germacrene D**	(Maccioni et al., 2007; Ormeño et al., 2007; Paolini et al., 2008; Llusià et al., 2010)	(Demetzos et al., 1994b)							(Demetzos et al., 1990b)		(Loizzo et al., 2013)
**Globulol**		(Demetzos et al., 1994b)				(Mariotti et al., 1997; Gomes et al., 2005)		(Angelopoulou et al., 2002)	(Angelopoulou et al., 2001b)		(Demetzos et al., 2002a; Loizzo et al., 2013)
**Guaia-6,10(14)-diene-4*β*-ol**	(Paolini et al., 2008)										
**Guaiazulene**			(Demetzos et al., 1997)								
**Guaiol**	(Maccioni et al., 2007)										(Loizzo et al., 2013)
**Humulene epoxide**									(Angelopoulou et al., 2001b)		(Demetzos et al., 2002a)
**Humulene oxide**											(Loizzo et al., 2013)
**Humulene-6,7-epoxide**	(Paolini et al., 2008)										
***iso*-Calamendiol**	(Paolini et al., 2008)										
**Isocaryophyllene**	(Ormeño et al., 2007)										
**Isogermacrene-D**	(Paolini et al., 2008)										
**Isoledene**											(Demetzos et al., 2002a)
***iso*-Shyobunone**	(Paolini et al., 2008)										
**Italicene**	(Maccioni et al., 2007)										
**Juniper camphor**	(Maccioni et al., 2007)										
**Juniperol acetate**	(Maccioni et al., 2007)										
**Khusinol**									(Angelopoulou et al., 2001b)		(Demetzos et al., 2002a)
**Ledene**		(Demetzos et al., 1994b)				(Mariotti et al., 1997)					

	C. albidus	*C. creticus* subsp. *creticus*	*C. creticus* subsp. *eriocephalus*	*C. clusii*	*C. crispus*	*C. ladanifer*	*C. laurifolius*	*C. monspeliensis*	*C. parviflorus*	*C. populifolius*	*C. salviifolius*
**Ledol**	(Paolini et al., 2008)	(Demetzos et al., 1995)	(Demetzos et al., 1997)			(Mariotti et al., 1997; Ramalho et al., 1999; Robles et al., 2003)		(Loizzo et al., 2013)			
**Longiborneol**									(Angelopoulou et al., 2001b)		(Demetzos et al., 2002a)
**Longifolene**		(Demetzos et al., 1994b)									
**Muurola-4,11-diene**	(Paolini et al., 2008)										
**Muurola-4,14-diene**								(Angelopoulou et al., 2002)			
**Nootkatone**											(Loizzo et al., 2013)
**Occidol**											(Demetzos et al., 2002a)
**Patchoulene**		(Demetzos et al., 1995)									
**Pregeijerene**	(Maccioni et al., 2007)										
**Salvial-4(14)-en-1-one**	(Paolini et al., 2008)										
**Shyobunone**	(Paolini et al., 2008)										
**Spathulenol**	(Maccioni et al., 2007; Paolini et al., 2008)		(Demetzos et al., 1997)			(Mariotti et al., 1997; Gomes et al., 2005)		(Robles and Garzino, 2000)	(Angelopoulou et al., 2001b)		(Demetzos et al., 2002a; Loizzo et al., 2013)
**Torreyol**											(Loizzo et al., 2013)
***trans-α*-Bergamotene**	(Llusià et al., 2010)										(Loizzo et al., 2013)
**Valerianol**	(Maccioni et al., 2007)										
**Viridiflorene**			(Demetzos et al., 1997)			(Gomes et al., 2005)					
**Viridiflorol**	(Paolini et al., 2008)	(Demetzos et al., 1994b)	(Demetzos et al., 1997)			(Mariotti et al., 1997; Robles et al., 2003; Gomes et al., 2005; Zidane et al., 2013)		(Angelopoulou et al., 2002; Maccioni et al., 2007)	(Angelopoulou et al., 2001b)		(Demetzos et al., 2002a; Loizzo et al., 2013)
**Vulgarol B**											(Loizzo et al., 2013)
**Widdrene**								(Jemia et al., 2013)			
**Widdrol**											(Loizzo et al., 2013)

	C. albidus	C. creticus subsp. creticus	C. creticus subsp. eriocephalus	C. clusii	C. crispus	C. ladanifer	C. laurifolius	C. monspeliensis	C. parviflorus	C. populifolius	C. salviifolius
**Xanthorrhizol**	(Paolini et al., 2008)										
**Zingiberene**	(Llusià et al., 2010)										
**Zingiberenol**	(Paolini et al., 2008)										
**α-Amorphene**			(Demetzos et al., 1997)								
**α-Bisabolol**	(Paolini et al., 2008)	(Demetzos et al., 1995)						(Robles and Garzino, 2000)			
**α-Cadinene**	(Paolini et al., 2008)		(Demetzos et al., 1997)						(Demetzos et al., 1990b; Angelopoulou et al., 2001b)		(Demetzos et al., 2002a)
**α-Cadinol**	(Maccioni et al., 2007; Paolini et al., 2008)							(Robles and Garzino, 2000 ; Angelopoulou et al., 2002; Loizzo et al., 2013);	(Angelopoulou et al., 2001b)		(Demetzos et al., 2002a; Loizzo et al., 2013)
**α-Calacorene**	(Paolini et al., 2008)							(Loizzo et al., 2013)	(Angelopoulou et al., 2001b)		(Demetzos et al., 2002a; Loizzo et al., 2013)
**α-Cedrene**	(Llusià et al., 2010)										
**α-Cedrol**											(Demetzos et al., 2002a)
**α-Copaene**	(Maccioni et al., 2007; Paolini et al., 2008)	(Demetzos et al., 1994b; Demetzos et al., 1995)	(Demetzos et al., 1997)			(Mariotti et al., 1997)		(Loizzo et al., 2013)	(Demetzos et al., 1990b)		(Demetzos et al., 2002a; Loizzo et al., 2013)
**α-Cubebene**	(Maccioni et al., 2007; Ormeño et al., 2007; Paolini et al., 2008)	(Demetzos et al., 1994b)	(Demetzos et al., 1997)			(Zidane et al., 2013)					
**α-Curcumene**	(Paolini et al., 2008; Llusià et al., 2010)										
**α-Elemene**											(Loizzo et al., 2013)

	C. albidus	*C. creticus* subsp. *creticus*	*C. creticus* subsp. *eriocephalus*	*C. clusii*	*C. crispus*	*C. ladanifer*	*C. laurifolius*	*C. monspeliensis*	*C. parviflorus*	*C. populifolius*	*C. salviifolius*
**α-*epi*-Cadinol**									(Angelopoulou et al., 2001b)		(Demetzos et al., 2002a)
**α-*epi*-Muurolol**									(Angelopoulou et al., 2001b)		(Demetzos et al., 2002a)
**α-Eudesmol**		(Demetzos et al., 1994b)						(Angelopoulou et al., 2002)			(Demetzos et al., 2002a)
**α-Guaiene**		(Demetzos et al., 1994b; Demetzos et al., 1995)							(Angelopoulou et al., 2001b)		(Demetzos et al., 2002a)
**α-Gurjunene**	(Ormeño et al., 2007; Paolini et al., 2008)					(Mariotti et al., 1997)		(Loizzo et al., 2013)			(Demetzos et al., 2002a)
**α-Humulene**	(Maccioni et al., 2007; Paolini et al., 2008)	(Demetzos et al., 1994b)				(Mariotti et al., 1997)			(Angelopoulou et al., 2001b)		(Demetzos et al., 2002a)
**α-Longipilene**	(Llusià et al., 2010)										
**α-Muurolene**	(Maccioni et al., 2007; Ormeño et al., 2007; Paolini et al., 2008)							(Angelopoulou et al., 2002)	(Demetzos et al., 1990b; Angelopoulou et al., 2001b)		(Demetzos et al., 2002a; Loizzo et al., 2013)
**α-Muurolol**	(Maccioni et al., 2007)								(Angelopoulou et al., 2001b)		(Demetzos et al., 2002a)
**α-Selinene**		(Demetzos et al., 1994b)									
**α-Sinensal**											(Loizzo et al., 2013)
**α-Ylangene**		(Demetzos et al., 1994b)							(Demetzos et al., 1990b)		
**α-Zingiberene**	(Maccioni et al., 2007; Ormeño et al., 2007; Paolini et al., 2008; Llusià et al., 2010)										
***β*-Bisabolene**	(Ormeño et al., 2007)		(Demetzos et al., 1997)								

	C. albidus	*C. creticus* subsp. *creticus*	*C. creticus* subsp. *eriocephalus*	*C. clusii*	*C. crispus*	*C. ladanifer*	*C. laurifolius*	*C. monspeliensis*	*C. parviflorus*	*C. populifolius*	*C. salviifolius*
***β*-Bourbonene**	(Maccioni et al., 2007; Ormeño et al., 2007; Paolini et al., 2008; Llusià et al., 2010)	(Demetzos et al., 1994b)							(Demetzos et al., 1990b)		
***β*-Cadinol**								(Loizzo et al., 2013)			
***β*-Calacorene**	(Paolini et al., 2008)							(Angelopoulou et al., 2001a)			(Demetzos et al., 2002a)
***β*-Caryophyllene**	(Maccioni et al., 2007; Ormeño et al., 2007; Paolini et al., 2008; Llusià et al., 2010)	(Demetzos et al., 1994b; Demetzos et al., 1995)						(Robles and Garzino, 2000; Rivoal et al., 2010; Loizzo et al., 2013)			(Loizzo et al., 2013)
***β*-Caryophyllene oxide**	(Paolini et al., 2008)										
***β*-Copaen-4-ol**								(Angelopoulou et al., 2001a)			(Demetzos et al., 2002a)
***β*-Copaene**	(Paolini et al., 2008)							(Angelopoulou et al., 2001a)			
***β*-Cubebene**	(Maccioni et al., 2007; Ormeño et al., 2007)										
***β*-Elemene**	(Ormeño et al., 2007; Paolini et al., 2008)		(Demetzos et al., 1997)								
***β*-Eudesmol**		(Demetzos et al., 1994b; Demetzos et al., 1995)				(Mariotti et al., 1997)			(Angelopoulou et al., 2001b)		(Demetzos et al., 2002a)
***β*-Germacrenol**	(Paolini et al., 2008)										
***β*-Gurjunene**	(Maccioni et al., 2007; Ormeño et al., 2007)	(Demetzos et al., 1995)						(Loizzo et al., 2013)			(Demetzos et al., 2002a)
***β*-Himachalene**	(Maccioni et al., 2007)	(Demetzos et al., 1994b)									(Demetzos et al., 2002a)

	C. albidus	*C. creticus* subsp. *creticus*	*C. creticus* subsp. *eriocephalus*	*C. clusii*	*C. crispus*	*C. ladanifer*	*C. laurifolius*	*C. monspeliensis*	*C. parviflorus*	*C. populifolius*	*C. salviifolius*
***β*-Oplopenone**	(Paolini et al., 2008)										
***β*-Selinene**		(Demetzos et al., 1994b; Demetzos et al., 1995)	(Demetzos et al., 1997)								(Demetzos et al., 2002a)
***β*-Sesquiphellandrene**	(Maccioni et al., 2007; Ormeño et al., 2007; Paolini et al., 2008; Llusià et al., 2010)										
***β*-trans-Farnesene**	(Ormeño et al., 2007)										
***β*-Yanglene**	(Paolini et al., 2008)										
***β*-Ylangene**									(Demetzos et al., 1990b)		
***γ*-Cadinene**	(Ormeño et al., 2007; Paolini et al., 2008)		(Demetzos et al., 1997)			(Mariotti et al., 1997)		(Angelopoulou et al., 2001a)			(Demetzos et al., 2002a; Loizzo et al., 2013)
***γ*-Curcumene**	(Maccioni et al., 2007)										
***γ*-Eudesmol**	(Maccioni et al., 2007)								(Angelopoulou et al., 2001b)		(Demetzos et al., 2002a)
**γ-Gurjunene**									(Angelopoulou et al., 2001b)		(Demetzos et al., 2002a)
***γ*-Muurolene**	(Maccioni et al., 2007; Ormeño et al., 2007)					(Mariotti et al., 1997)					(Demetzos et al., 2002a; Loizzo et al., 2013)
***δ*-Cadinene**	(Maccioni et al., 2007; Ormeño et al., 2007; Paolini et al., 2008)	(Demetzos et al., 1994b; Demetzos et al., 1995)	(Demetzos et al., 1997)			(Mariotti et al., 1997; Gomes et al., 2005; Zidane et al., 2013)		(Angelopoulou et al., 2001a)	(Demetzos et al., 1990b; Angelopoulou et al., 2001b)		(Demetzos et al., 2002a; Loizzo et al., 2013)

	C. albidus	*C. creticus* subsp. *creticus*	*C. creticus* subsp. *eriocephalus*	*C. clusii*	*C. crispus*	*C. ladanifer*	*C. laurifolius*	*C. monspeliensis*	*C. parviflorus*	*C. populifolius*	*C. salviifolius*
**δ-Elemene**	(Maccioni et al., 2007)										
**δ-Selinene**		(Demetzos et al., 1995)									
**τ-Cadinol**	(Maccioni et al., 2007; Paolini et al., 2008)										(Loizzo et al., 2013)
**τ-Muurolol**	(Maccioni et al., 2007; Paolini et al., 2008)							(Loizzo et al., 2013)			(Loizzo et al., 2013)
Diterpenes											
**(*E*)-Phytol**								(Jemia et al., 2013)			(Loizzo et al., 2013)
**(*Z*)-Phytol**								(Loizzo et al., 2013)			(Loizzo et al., 2013)
**4-*epi*-Abietal**									(Angelopoulou et al., 2001b)		(Demetzos et al., 2002a)
**4-*epi*-Dehydroabietol**									(Angelopoulou et al., 2001b)		(Demetzos et al., 2002a)
**Abieta-7,13-dien-3-one**									(Angelopoulou et al., 2001b)		(Demetzos et al., 2002a)
**Abieta-8,11,13-trien-7-one**									(Angelopoulou et al., 2001b)		(Demetzos et al., 2002a)
**Abietadiene**								(Angelopoulou et al., 2002)	(Angelopoulou et al., 2001b)		(Demetzos et al., 2002a)
**Abietal**									(Angelopoulou et al., 2001b)		(Demetzos et al., 2002a)
**Abietatriene**									(Angelopoulou et al., 2001b)		(Demetzos et al., 2002a; Loizzo et al., 2013)
**Abietol**									(Angelopoulou et al., 2001b)		
**Cembrene**								(Angelopoulou et al., 2002; Jemia et al., 2013)			

	C. albidus	*C. creticus* subsp. *creticus*	*C. creticus* subsp. *eriocephalus*	*C. clusii*	*C. crispus*	*C. ladanifer*	*C. laurifolius*	*C. monspeliensis*	*C. parviflorus*	*C. populifolius*	*C. salviifolius*
***cis*-Ferruginol**									(Angelopoulou et al., 2001b)		(Demetzos et al., 2002a)
***cis*-Totarol**								(Angelopoulou et al., 2002)	(Angelopoulou et al., 2001b)		(Demetzos et al., 2002a)
**Dehydro abietal**								(Angelopoulou et al., 2002)	(Angelopoulou et al., 2001b)		(Demetzos et al., 2002a)
**Dehydro abietic acid**								(Angelopoulou et al., 2002)			
**Dehydro abietol**									(Angelopoulou et al., 2001b)		(Demetzos et al., 2002a)
**Geranyl *α*-terpinene**	(Paolini et al., 2008)										
**Geranyl linalool**	(Paolini et al., 2008)										
**Geranyl *p*-cymene**	(Paolini et al., 2008)										
**Isohibaene**								(Angelopoulou et al., 2001a)	(Angelopoulou et al., 2001b)		(Demetzos et al., 2002a)
**Isokaurene**									(Angelopoulou et al., 2001b)		(Demetzos et al., 2002a)
**Isopimara-9(11),15-diene**									(Angelopoulou et al., 2001b)		(Demetzos et al., 2002a)
**Kaurene**											(Demetzos et al., 2002a)
**Kaur-15-ene**								(Angelopoulou et al., 2001a; Loizzo et al., 2013)			
**Kaur-16-ene**		(Demetzos et al., 1995)						(Angelopoulou et al., 2001a; Angelopoulou et al., 2002; Loizzo et al., 2013)			
**Methyl-*neo*-abietane**								(Angelopoulou et al., 2002)			
**Nezukol**								(Angelopoulou et al., 2002)	(Angelopoulou et al., 2001b)		(Demetzos et al., 2002a)
**Phyllocladanol**									(Angelopoulou et al., 2001b)		(Demetzos et al., 2002a)
**Phyllocladene**									(Angelopoulou et al., 2001b)		(Demetzos et al., 2002a)

	C. albidus	C. creticus subsp. creticus	C. creticus subsp. eriocephalus	C. clusii	C. crispus	C. ladanifer	C. laurifolius	C. monspeliensis	C. parviflorus	C. populifolius	C. salviifolius
**Pimara-8(9)15-diene**											(Demetzos et al., 2002a)
**Pimaradiene**									(Angelopoulou et al., 2001b)		
**Rimuene**											(Demetzos et al., 2002a)
***trans*-Ferruginol**									(Angelopoulou et al., 2001b)		(Demetzos et al., 2002a)
***trans*-Ferruginyl acetate**											(Demetzos et al., 2002a)
***trans*-Totarol**									(Angelopoulou et al., 2001b)		(Demetzos et al., 2002a)
**Vulgarol A**								(Loizzo et al., 2013)			
Labdane type diterpenes											
**2-Keto-13-*epi*-manoyl oxide**									(Angelopoulou et al., 2001b)		(Demetzos et al., 2002a)
**2-Keto-manoyl oxide**								(Jemia et al., 2013)	(Angelopoulou et al., 2001b)		(Demetzos et al., 2002a)
**3*β*-Acetoxy 8,13-diepi manoyl oxide**									(Angelopoulou et al., 2001b)		
**3*β*-Acetoxy-manoyl oxide**									(Angelopoulou et al., 2001b)		(Demetzos et al., 2002a)
**3*β*-Acetoxy-13-*epi*-manoyl oxide**		(Demetzos et al., 1994a; Anastasaki et al., 1999; Demetzos et al., 2002b; Falara et al., 2010)						(Angelopoulou et al., 2001a; Angelopoulou et al., 2002)	(Angelopoulou et al., 2001b)		(Demetzos et al., 2002a)
**3*β*-Hydroxy-manool**									(Angelopoulou et al., 2001b)		(Demetzos et al., 2002a)
**3*β*-Hydroxy-manoyl oxide**											(Demetzos et al., 2002a)

	C. albidus	*C. creticus* subsp. *creticus*	*C. creticus* subsp. *eriocephalus*	*C. clusii*	*C. crispus*	*C. ladanifer*	*C. laurifolius*	*C. monspeliensis*	*C. parviflorus*	*C. populifolius*	*C. salviifolius*
**3*β*-Hydroxy-13-*epi*-manoyl-oxide**		(Demetzos et al., 1994a; Anastasaki et al., 1999; Demetzos et al., 1999; Demetzos et al., 2002b; Falara et al., 2010)						(Angelopoulou et al., 2002); (Jemia et al., 2013)	(Angelopoulou et al., 2001b)		(Demetzos et al., 2002a)
**6*β*,8-Dihydroxy-*ent*-13*(E)*-labden-15-oic acid (laurifolic acid)**							(De Pascual Teresa et al., 1986)				
**6-acetoxy-7-oxo-8-Labden-15-oic acid**						(Alías et al., 2012)					
**7*α*-Hydroxy manool**									(Angelopoulou et al., 2001b)		(Demetzos et al., 2002a)
**7-oxo-8-Labden-15-oic acid**						(Alías et al., 2012)					
**8(17)-Labden-15-ol**								(Angelopoulou et al., 2002)			
**8*α*,15-Labdanediol**								(Berti et al., 1970)			
**8,13-Epoxy-15,16-dinor-labda-12-en**		(Falara et al., 2010)						(Angelopoulou et al., 2002)			
**10-*α*-Labdan-14-oic acid methyl ether**				(Tomás-Menor et al., 2013)							
**11,13-Labdadien-8-yl-acetate**								(Angelopoulou et al., 2002)			
**13-*epi*-Manool**		(Demetzos et al., 1995)						(Angelopoulou et al., 2002)	(Angelopoulou et al., 2001b)		(Demetzos et al., 2002a)
**13-*epi*-Manoyl oxide (13-epi-8,13-epoxylabd-14-en)**	(Paolini et al., 2008)	(Demetzos et al., 1990a; Demetzos et al., 1994a; Demetzos et al., 1994b; Demetzos et al., 1995; Anastasaki et al., 1999; Falara et al., 2010)	(Demetzos et al., 1997)					(Angelopoulou et al., 2001a; Angelopoulou et al., 2002; Demetzos et al., 2002b; Loizzo et al., 2013)	(Angelopoulou et al., 2001b)		(Demetzos et al., 2002a; Loizzo et al., 2013)

	C. albidus	*C. creticus* subsp. *creticus*	*C. creticus* subsp. *eriocephalus*	*C. clusii*	*C. crispus*	*C. ladanifer*	*C. laurifolius*	*C. monspeliensis*	*C. parviflorus*	*C. populifolius*	*C. salviifolius*
**15-Acetoxylabdan-8-ol**						(Mariotti et al., 1997)					
**15-nor-Labdan-8-ol**						(Gomes et al., 2005)					
**15,16-Dinor-labd-8(20)-ene-13-one**								(Angelopoulou et al., 2001a; Angelopoulou et al., 2002)	(Angelopoulou et al., 2001b)		
**di-[Labda-13-en, 8α-ol-15-yl] malonate**		(Demetzos et al., 1994c)									
**Dinorlabda-8(20)-en-13-one**											(Demetzos et al., 2002a)
**Labda-7,13-dien-15-ol**		(Demetzos et al., 1990a; Demetzos et al., 1994a; Anastasaki et al., 1999; Falara et al., 2010)	(Anastasaki et al., 1999)								(Demetzos et al., 2002a)
**Labda-7,13-dien-15-yl acetate**		(Demetzos et al., 1990a; Demetzos et al., 1994a; Anastasaki et al., 1999; Falara et al., 2010)	(Anastasaki et al., 1999)								
**Labda-7,13-dien-15-yl malonic acid**		(Demetzos et al., 1994c)									
**Labd-7,13-dien-15-yl methyl malonic acid diester**		(Demetzos et al., 1994c)									
**[Labd-7,13-dien-15-yl] malonate**		(Demetzos et al., 1994c)									
**Labda-8,14-dien**								(Angelopoulou et al., 2002)			

	C. albidus	*C. creticus* subsp. *creticus*	*C. creticus* subsp. *eriocephalus*	*C. clusii*	*C. crispus*	*C. ladanifer*	*C. laurifolius*	*C. monspeliensis*	*C. parviflorus*	*C. populifolius*	*C. salviifolius*
**Labda-13-en-8*α*,15-diol**		(Demetzos et al., 1990a; Demetzos et al., 1994a; Anastasaki et al., 1999; Falara et al., 2010)	(Anastasaki et al., 1999; Demetzos et al., 2001b)					(Angelopoulou et al., 2002)	(Angelopoulou et al., 2001b)		(Demetzos et al., 2002a)
**Labda-13-en-8*α*,15-yl acetate**		(Anastasaki et al., 1999; Falara et al., 2010)	(Anastasaki et al., 1999; Demetzos et al., 2001b)					(Angelopoulou et al., 2002)	(Angelopoulou et al., 2001b)		(Demetzos et al., 2002a)
**Labda-13-en-8-ol**		(Demetzos et al., 1995)									
**Labda-13-en-8-yl acetate**		(Demetzos et al., 1995)	(Demetzos et al., 1997)								
**Labda-13-en-8-yl-acetate isomer**			(Demetzos et al., 1997)								
**Labda-13(16),14-dien-8-ol**			(Demetzos et al., 1997)								
**Labdanolic acid**						(Tomás-Menor et al., 2013)					
**Larixol**									(Angelopoulou et al., 2001b)		(Demetzos et al., 2002a)
**Manool**		(Demetzos et al., 1995)	(Demetzos et al., 1997)						(Angelopoulou et al., 2001b)		(Demetzos et al., 2002a)
**Manoyl oxide (8,13-epoxylabd-14-ene)**		(Demetzos et al., 1990a; Demetzos et al., 1994b; Demetzos et al., 1995; Anastasaki et al., 1999)	(Demetzos et al., 1997; Anastasaki et al., 1999)					(Angelopoulou et al., 2001a; Angelopoulou et al., 2002; Loizzo et al., 2013; Jemia et al., 2013)	(Demetzos et al., 1990b; Angelopoulou et al., 2001b)		(Demetzos et al., 2002a; Loizzo et al., 2013)
**Manoyl oxide isomer**		(Demetzos et al., 1995; Anastasaki et al., 1999; Demetzos et al., 1999)	(Anastasaki et al., 1999)					(Angelopoulou et al., 2001a; Angelopoulou et al., 2002)	(Angelopoulou et al., 2001b)		(Demetzos et al., 2002a)

	C. albidus	*C. creticus* subsp. *creticus*	*C. creticus* subsp. *eriocephalus*	*C. clusii*	*C. crispus*	*C. ladanifer*	*C. laurifolius*	*C. monspeliensis*	*C. parviflorus*	*C. populifolius*	*C. salviifolius*
***epi*-Manoyl oxide**								(Robles and Garzino, 2000)			
**Methyl neoabietate**	(Llusià et al., 2010)										
**Methyl-labd-8(17)-en-15-oate**		(Demetzos et al., 1995)									
**Oxocativic acid**						(Alías et al., 2012)					
**Sclareol (Labd-14-ene-8,13-diol)**		(Demetzos et al., 1990a; Demetzos et al., 1994b; Demetzos et al., 1999)						(Angelopoulou et al., 2002)			
**Sclareoloxide**						(Gomes et al., 2005)					
Clerodane Diterpenes											
**19-Acetoxy-*cis*-clerodan-3-en-15-oic acid**								(Demetzos et al., 2001b; Kolocouris et al., 2001)			
**19-Acetoxy-kolavan-3-en-15-oic acid**								(Angelopoulou et al., 2002)			
**15,18,19-Trihydroxy-clerod-3-ene**								(Angelopoulou et al., 2002)			
**15,18-Diacetoxy-3-ene-*cis*-clerodane**								(Kalpoutzakis et al., 2003)			
**15,18-Dihydroxy-*cis*-clerodan-3-ene (Cistodiol)**								(Berti et al., 1970; Kalpoutzakis et al., 2003)			
**Cistodioic acid**								(Berti et al., 1970)			
**15-Acetoxy-3-ene-*cis*-clerodan-18-ol**								(Kalpoutzakis et al., 2003)			

	C. albidus	*C. creticus* subsp. *creticus*	*C. creticus* subsp. *eriocephalus*	*C. clusii*	*C. crispus*	*C. ladanifer*	*C. laurifolius*	*C. monspeliensis*	*C. parviflorus*	*C. populifolius*	*C. salviifolius*
**15-Acetoxy-*cis*-clerodan-3-ene-18-al**								(Kalpoutzakis et al., 2003)			
**15-Acetoxy-*cis*-clerodan-3-ene-18-oic acid**								(Kalpoutzakis et al., 2003)			
**15-Hydroxy-*cis*-clerodan-3-ene-18-al**								(Kalpoutzakis et al., 2003)			
**15-Hydroxy-*cis*-clerodan-3-ene-18-oic acid**								(Kalpoutzakis et al., 2003)			
**18,19-Epoxy-18*β*-methoxy-ent-clerod-3-ene-15-oic acid**								(Angelopoulou et al., 2001a)			
**18-Acetoxy-3-ene-*cis*-clerodan-15-ol**								(Kalpoutzakis et al., 2003)			
**18-Acetoxy-*cis*-clerodan-3-ene-15-oic acid**								(Kalpoutzakis et al., 2003)			
**18-Acetoxy-kolavan-3-en-15-oic acid**								(Angelopoulou et al., 2001a; Angelopoulou et al., 2002)			
**18-Hydroxy-*cis*-clerodan-3-ene-15-oic acid**								(Kalpoutzakis et al., 2003)			
**2*α*,3*β*-Dihydroxy-4(18)-*neo*-cleroden-15-oate**										(Urones et al., 1995b)	
**3,4-Dehydroclerodanoic acid methyl ester**								(Angelopoulou et al., 2002)			
**7*β*-Hydroxy-ent-clerod-3-en-15-oic acid**								(Angelopoulou et al., 2001a)			

	C. albidus	*C. creticus* subsp. *creticus*	*C. creticus* subsp. *eriocephalus*	*C. clusii*	*C. crispus*	*C. ladanifer*	*C. laurifolius*	*C. monspeliensis*	*C. parviflorus*	*C. populifolius*	*C. salviifolius*
***cis*-Clerodan-3-ene-15-oic acid (epi-populifolic acid)**								(Kalpoutzakis et al., 2003)			
**Cistodiolic acid**								(Angelopoulou et al., 2002)			
**Clerod-3-en-15-oic acid methyl ester**								(Angelopoulou et al., 2002)			
**Methyl 1,3-*neo*-clerodadien-15-oate**										(Urones et al., 1994)	
**Methyl 2,4(18)-neoclerodadien-15-oate**										(Urones et al., 1994)	
**Methyl 2-oxo-3-neo-cleroden-15-oate**										(Urones et al., 1994)	
**Methyl 2*α*,3*β*,4*β*-trihydroxy-*neo*-clerodan-15-oate**										(Urones et al., 1995a)	
**Methyl 2*α*-acetoxy-3-neo-cleroden-15-oate,**										(Urones et al., 1994)	
**Methyl 2*α*-hydroxy-3-neo-cleroden-15-oate**										(Urones et al., 1994)	
**Methyl 2*α*-methoxy-3-neo-cleroden-15-oate**										(Urones et al., 1994)	
**Methyl 2*β*-hydroxy-3-neo-cleroden-15-oate**										(Urones et al., 1994)	
**Methyl 3-*neo*-cleroden-15-oate**										(Urones et al., 1994)	

	C. albidus	*C. creticus* subsp. *creticus*	*C. creticus* subsp. *eriocephalus*	*C. clusii*	*C. crispus*	*C. ladanifer*	*C. laurifolius*	*C. monspeliensis*	*C. parviflorus*	*C. populifolius*	*C. salviifolius*
**Nor-infuscaic acid methyl ester**								(Angelopoulou et al., 2002)			
**Salmantic acid**							(de Pascual Teresa et al., 1983)				
**Salmantic acid methyl ester**							(de Pascual Teresa et al., 1983)				
**Salmantidiol**							(de Pascual Teresa et al., 1983)				
**PHENYLPROPANOIDS**											
**2-Phenylethanol**						(Ramalho et al., 1999)					
**Elemicin**											(Loizzo et al., 2013)
**Methylisoeugenol**								(Loizzo et al., 2013)			
**Scopoletin**							(Vogt and Gerhard Gul, 1994)				
**Tetrahydroxystilbene glucoside**											(Tomás-Menor et al., 2013)
Flavonoids											
**(-)-(*epi*)Catechin**	(Barrajón-Catalán et al., 2011; Tomás-Menor et al., 2013)			(Hernández et al., 2004; Hernández et al., 2011)	(Barrajón-Catalán et al., 2011)						(Tomás-Menor et al., 2013)
**(-)-(*epi*)Catechin gallate**				(Hernández et al., 2004)							
**(-)-(*epi*)Catechin-(*epi*)gallocatechin dimer**	(Barrajón-Catalán et al., 2011)				(Barrajón-Catalán et al., 2011)						

	C. albidus	*C. creticus* subsp. *creticus*	*C. creticus* subsp. *eriocephalus*	*C. clusii*	*C. crispus*	*C. ladanifer*	*C. laurifolius*	*C. monspeliensis*	*C. parviflorus*	*C. populifolius*	*C. salviifolius*
**(-)-(*epi*)Gallocatechin**	(Barrajón-Catalán et al., 2011)				(Barrajón-Catalán et al., 2011)		(Barrajón-Catalán et al., 2011)				
**(-)-(*epi*)Gallocatechin gallate**				(Hernández et al., 2004; Hernández et al., 2011)							(Barrajón-Catalán et al., 2011; Tomás-Menor et al., 2013)
**(-)-(*epi*)Gallocatechin-(*epi*)gallocatechin dimer**	(Barrajón-Catalán et al., 2011)			(Barrajón-Catalán et al., 2011)							(Tomás-Menor et al., 2013)
**(−)-Epicatechin-*O*-quinone**				(Hernández et al., 2011)							
**(−)-Epigallocatechin gallate-*O*-quinone**				(Hernández et al., 2011)							
**3,4’-*O*-Dimethyl quercetin**							(Sadhu et al., 2006)				
**3,4'-*O*-Dimethyl kaempferol**							(Sadhu et al., 2006)				
**3,7,4',5'-Tetramethyl ether myricetin**								(Demetzos et al., 2001a)			
**3,7-*O*-Dimethyl kaempferol**							(Sadhu et al., 2006; Akkol et al., 2012)				
**3,7-*O*-Dimethyl quercetin**							(Sadhu et al., 2006; Akkol et al., 2012)				
**3-Methoxyquercetin**							(Orhan et al., 2013)				
**3-*O*-Methyl quercetin**							(Sadhu et al., 2006; Akkol et al., 2012)				
**3-*O*-*p*-Hydroxybenzoyl ester of epigallocatechin**											(Danne et al., 1994)

	C. albidus	*C. creticus* subsp. *creticus*	*C. creticus* subsp. *eriocephalus*	*C. clusii*	*C. crispus*	*C. ladanifer*	*C. laurifolius*	*C. monspeliensis*	*C. parviflorus*	*C. populifolius*	*C. salviifolius*
**6-*OH*-Kaempferol-3,6,4'-trimethyl ether**									(Vogt et al., 1987)		
**6-*OH*-Kaempferol-3,6-dimethyl ether**									(Vogt et al., 1987)		
**Apigenin**		(Demetzos et al., 1990a)		(Tomás-Menor et al., 2013)		(Fernández-Arroyo et al., 2010; Tomás-Menor et al., 2013)	(Vogt and Gerhard Gul, 1994; Sadhu et al., 2006; Orhan et al., 2013)				
**Apigenin diglucoside**	(Barrajón-Catalán et al., 2011)					(Barrajón-Catalán et al., 2011)		(Barrajón-Catalán et al., 2011)			
**Apigenin diglycoside**											
**Apigenin methylether**				(Tomás-Menor et al., 2013)		(Fernández-Arroyo et al., 2010; Tomás-Menor et al., 2013)					
**Apigenin-7-methyl ether**							(Vogt and Gerhard Gul, 1994)				
**Arbutin**											(Tomás-Menor et al., 2013)
**Catechin**	(Qa'dan et al., 2003)							(Pomponio et al., 2003)			(Danne et al., 1994; Qa'Dan et al., 2006)
**Catechin-3-*O*-*α*-*L*-rhamnopyranoside**											(Tomás-Menor et al., 2013)
**di-Coumaroyl kaempferol glucoside**											(Saracini et al., 2005)
**Dimethoxyapigenin**							(Orhan et al., 2013)				
**Dimethoxykaempferol**							(Orhan et al., 2013)				
**Dimethoxyquercetin**							(Orhan et al., 2013)				
**Epicatechin**											(Danne et al., 1994; Qa'Dan et al., 2006)

	C. albidus	*C. creticus* subsp. *creticus*	*C. creticus* subsp. *eriocephalus*	*C. clusii*	*C. crispus*	*C. ladanifer*	*C. laurifolius*	*C. monspeliensis*	*C. parviflorus*	*C. populifolius*	*C. salviifolius*
**Epicatechin-3-*O*-gallate**											(Danne et al., 1994; Qa'Dan et al., 2006)
**Epigallocatechin**	(Tomás-Menor et al., 2013)			(Tomás-Menor et al., 2013)		(Fernández-Arroyo et al., 2010)					(Danne et al., 1994; Qa'Dan et al., 2006; Tomás-Menor et al., 2013)
**Epigallocatechin-(4*β*,6)-epigallocatechin-3-*O*-gallate**											(Qa'Dan et al., 2006)
**Epigallocatechin-(4*β*,8)-epigallocatechin**											(Danne et al., 1994; Qa'Dan et al., 2006)
**Epigallocatechin-(4*β*,8)-epigallocatechin-3-*O*-gallate**											(Danne et al., 1994)
**Epigallocatechin-(4*β*,8)-gallocatechin-(4*α*,8)-catechin**	(Qa'dan et al., 2003)										
**Epigallocatechin-(4*β*,8)-gallocatechin-(4*α*,8)-gallocatechin**	(Qa'dan et al., 2003)										
**Epigallocatechin-3-*O*-(4-hydroxybenzoate)**											(Danne et al., 1994; Qa'Dan et al., 2006)
**Epigallocatechin-3-*O*-gallate**											(Danne et al., 1994; Qa'Dan et al., 2006)
**Epigallocatechin-3-*O*-gallate-(4*β*,8)-epigallocatechin**											(Qa'Dan et al., 2006)
**Esculin**		(Demetzos et al., 1989; Demetzos et al., 1990a)									
**Gallocatechin**	(Qa'dan et al., 2003)							(Pomponio et al., 2003)			(Danne et al., 1994; Qa'Dan et al., 2006)

	C. albidus	*C. creticus* subsp. *creticus*	*C. creticus* subsp. *eriocephalus*	*C. clusii*	*C. crispus*	*C. ladanifer*	*C. laurifolius*	*C. monspeliensis*	*C. parviflorus*	*C. populifolius*	*C. salviifolius*
**Gallocatechin-(4α-8)-gallocatechin**											(Tomás-Menor et al., 2013)
**Gallocatechin-3-*O*-gallate**											(Danne et al., 1994)
**Genkwanin**							(Sadhu et al., 2006)				
**Gossypetin-3,8,3’,4’-tetramethyl ether**									(Vogt et al., 1987)		
**Gossypetin-3,8,3’-trimethyl ether**									(Vogt et al., 1987)		
**Herbacetin-3,8,4’-trimethyl ether**									(Vogt et al., 1987)		
**Herbacetin-3,8-dimethyl ether**									(Vogt et al., 1987)		
**Isorhamnetin-3-glucoside**						(Tomás-Lorente et al., 1992)					
**Isorhamnetin-3-xylosyl-(1,6)-glucoside**						(Tomás-Lorente et al., 1992)					
**Isorhamnetin-*O*-rutinoside**				(Barrajón-Catalán et al., 2011; Tomás-Menor et al., 2013)							
**Kaempferol**		(Demetzos et al., 1990a)				(Tomás-Lorente et al., 1992)					
**Kaempferol diglucoside**				(Barrajón-Catalán et al., 2011)		(Fernández-Arroyo et al., 2010)					
**Kaempferol diglycoside**				(Tomás-Menor et al., 2013)		(Fernández-Arroyo et al., 2010; Tomás-Menor et al., 2013)					(Tomás-Menor et al., 2013)
**Kaempferol dimethylether**				(Tomás-Menor et al., 2013)		(Fernández-Arroyo et al., 2010; Tomás-Menor et al., 2013)					

	C. albidus	*C. creticus* subsp. *creticus*	*C. creticus* subsp. *eriocephalus*	*C. clusii*	*C. crispus*	*C. ladanifer*	*C. laurifolius*	*C. monspeliensis*	*C. parviflorus*	*C. populifolius*	*C. salviifolius*
**Kaempferol methylether**						(Fernández-Arroyo et al., 2010)					
**Kaempferol-3,4'-dimethyl ether**									(Vogt et al., 1987)		
**Kaempferol-3,7,4'-trimethyl ether**									(Vogt et al., 1987)		
**Kaempferol-3,7-dimethyl ether**							(Vogt and Gerhard Gul, 1994)		(Vogt et al., 1987)		
**Kaempferol-3-glucoside**						(Tomás-Lorente et al., 1992)	(Vogt and Gerhard Gul, 1994)				
**Kaempferol-3-methyl ether**		(Demetzos et al., 1990a)					(Vogt and Gerhard Gul, 1994)		(Vogt et al., 1987)		
**Kaempferol-3-*O-β-D*-glucopyranoside**		(Demetzos et al., 1989)									
**Kaempferol-3-rhamnoside-glucoside**							(Vogt and Gerhard Gul, 1994)				
**Kaempferol-3-rutinoside**	(Tomás-Menor et al., 2013)					(Tomás-Lorente et al., 1992)					
**Kaempferol-4-monomethyl ether**									(Vogt et al., 1987)		
**Kaempferol-7-monomethyl ether**									(Vogt et al., 1987)		
**Luteolin**		(Demetzos et al., 1990a)					(Vogt and Gerhard Gul, 1994)				
**Luteolin-7-methyl ether**							(Vogt and Gerhard Gul, 1994)				
**Methoxyapigenin**							(Orhan et al., 2013)				
**Mono-coumaroyl kaempferol glucoside**											(Saracini et al., 2005)
**Myricetin**		(Demetzos et al., 1990a)				(Tomás-Lorente et al., 1992)					(Tomás-Menor et al., 2013)

	C. albidus	*C. creticus* subsp. *creticus*	*C. creticus* subsp. *eriocephalus*	*C. clusii*	*C. crispus*	*C. ladanifer*	*C. laurifolius*	*C. monspeliensis*	*C. parviflorus*	*C. populifolius*	*C. salviifolius*
**Myricetin hexoside**	(Tomás-Menor et al., 2013)				(Barrajón-Catalán et al., 2011)		(Barrajón-Catalán et al., 2011)				(Barrajón-Catalán et al., 2011; Tomás-Menor et al., 2013)
**Myricetin xyloside**											(Saracini et al., 2005)
**Myricetin-3,7,3’,4’-tetramethyl ether**								(Berti et al., 1967)	(Vogt et al., 1987)		
**Myricetin-3-arabinoside**							(Vogt and Gerhard Gul, 1994)				(Tomás-Menor et al., 2013)
**Myricetin-3-galactoside**							(Vogt and Gerhard Gul, 1994)				
**Myricetin-3-glucoside**						(Tomás-Lorente et al., 1992)					
**Myricetin-3-glucoside-rhamnoside**							(Vogt and Gerhard Gul, 1994)				
**Myricetin-3-*O*-(6''-*O*-galloylgalactoside)**											(Saracini et al., 2005)
**Myricetin-3-*O*-galactoside**											(Saracini et al., 2005)
**Myricetin-3-*O*-*α*-*L*-rhamnopyranoside**		(Demetzos et al., 1989)									
**Myricetin-3-*O*-*β*-*D*-galactopyranoside**		(Demetzos et al., 1989)									
**Myricetin-3-rhamnoside**							(Vogt and Gerhard Gul, 1994)				
**Myricetin-3-rutinoside**						(Tomás-Lorente et al., 1992)					
**Myricitrin**	(Barrajón-Catalán et al., 2011)				((Barrajón-Catalán et al., 2011)		(Barrajón-Catalán et al., 2011)	(Barrajón-Catalán et al., 2011)			
**Naringenin**							(Orhan et al., 2013)				
**Naringenin dihexoside**											(Tomás-Menor et al., 2013)

	C. albidus	*C. creticus* subsp. *creticus*	*C. creticus* subsp. *eriocephalus*	*C. clusii*	*C. crispus*	*C. ladanifer*	*C. laurifolius*	*C. monspeliensis*	*C. parviflorus*	*C. populifolius*	*C. salviifolius*
**Phenethyl-*β*-primeveroside**						(Fernández-Arroyo et al., 2010)					
**Quercetagetin-3,6,3’-trimethyl ether**									(Vogt et al., 1987)		
**Quercetin**		(Demetzos et al., 1990a)				(Tomás-Lorente et al., 1992)	(Vogt and Gerhard Gul, 1994;Orhan et al., 2013)				(Tomás-Menor et al., 2013)
**Quercetin coumaroyl hexoside**											(Tomás-Menor et al., 2013)
**Quercetin diglycoside**						(Fernández-Arroyo et al., 2010)					(Tomás-Menor et al., 2013)
**Quercetin glucoside**	(Tomás-Menor et al., 2013)				(Barrajón-Catalán et al., 2011)	(Fernández-Arroyo et al., 2010)	(Barrajón-Catalán et al., 2011)			(Barrajón-Catalán et al., 2011)	(Barrajón-Catalán et al., 2011; Tomás-Menor et al., 2013)
**Quercetin glycoside**				(Tomás-Menor et al., 2013)							(Tomás-Menor et al., 2013)
**Quercetin xyloside**											(Saracini et al., 2005)
**Quercetin xylosil glucoside**											(Saracini et al., 2005)
**Quercetin-3,3’-dimethyl ether**							(Vogt and Gerhard Gul, 1994)		(Vogt et al., 1987)		
**Quercetin-3,4’-dimethyl ether**							(Vogt and Gerhard Gul, 1994)				
**Quercetin-3,5,3’-trimethyl ether**							(Vogt et al., 1988); (Vogt and Gerhard Gul, 1994)				
**Quercetin-3,7,3',4'-tetramethyl ether (Retusin)**									(Vogt et al., 1987)		
**Quercetin-3,7,3’-trimethyl ether**							(Vogt and Gerhard Gul, 1994)		(Vogt et al., 1987)		
**Quercetin-3,7-dimethyl ether**							(Vogt and Gerhard Gul, 1994)				

	C. albidus	*C. creticus* subsp. *creticus*	*C. creticus* subsp. *eriocephalus*	*C. clusii*	*C. crispus*	*C. ladanifer*	*C. laurifolius*	*C. monspeliensis*	*C. parviflorus*	*C. populifolius*	*C. salviifolius*
**Quercetin-3-arabinoside**							(Vogt and Gerhard Gul, 1994)				
**Quercetin-3-arabinoside-glucoside**							(Vogt and Gerhard Gul, 1994)				
**Quercetin-3-galactoside**							(Vogt and Gerhard Gul, 1994)				
**Quercetin-3-galactoside-7-rhamnoside**						(Tomás-Lorente et al., 1992)					
**Quercetin-3-glucoside**						(Tomás-Lorente et al., 1992)					
**Quercetin-3-glucoside-rhamnoside**							(Vogt and Gerhard Gul, 1994)				
**Quercetin-3-methyl ether (Isorhamnetin)**						(Tomás-Lorente et al., 1992)	(Vogt and Gerhard Gul, 1994)				
**Quercetin-3-*O*-(2'-cumaroyl) rutinoside**	(Barrajón-Catalán et al., 2011)										
**Quercetin-3-*O*-(6''-*O*-galloylgalactoside)**											(Saracini et al., 2005)
**Quercetin-3-*O*-galactoside**											(Saracini et al., 2005)
**Quercetin-3-*O*-glucuronide**											(Saracini et al., 2005)
**Quercetin-3-*O*-rutinoside**	(Tomás-Menor et al., 2013)										
**Quercetin-3-*O*-α-rhamnoside (Quercitrin)**	(Barrajón-Catalán et al., 2011)						(Sadhu et al., 2006; Barrajón-Catalán et al., 2011; Orhan et al., 2013)				

	C. albidus	*C. creticus* subsp. *creticus*	*C. creticus* subsp. *eriocephalus*	*C. clusii*	*C. crispus*	*C. ladanifer*	*C. laurifolius*	*C. monspeliensis*	*C. parviflorus*	*C. populifolius*	*C. salviifolius*
**Quercetin-3-*O-β-D*-glucopyranoside**		(Demetzos et al., 1989)									
**Quercetin-3-*O-β-D*-rutinoside**		(Demetzos et al., 1989)									
**Quercetin-3-rhamnoside**							(Vogt and Gerhard Gul, 1994)				
**Quercetin-3-rhamnosyl-(1,2)-galactoside**						(Tomás-Lorente et al., 1992)					
**Quercetin-3-rutinoside**						(Tomás-Lorente et al., 1992)					
**Quercetin-3-sophoroside**						(Tomás-Lorente et al., 1992)					
**Quercetin-3-xylosyl-(1,6)-glucoside**						(Tomás-Lorente et al., 1992)					
**Quercetin-5,3'–dimethyl ether**							(Vogt et al., 1988; Vogt and Gerhard Gul, 1994)				
**Quercetin-7-3'-dimethyl ether**							(Vogt and Gerhard Gul, 1994)				
**Quercetin-7-methyl ether**							(Vogt and Gerhard Gul, 1994)				
**Rutin**	(Barrajón-Catalán et al., 2011)			(Barrajón-Catalán et al., 2011)							

	C. albidus	*C. creticus* subsp. *creticus*	*C. creticus* subsp. *eriocephalus*	*C. clusii*	*C. crispus*	*C. ladanifer*	*C. laurifolius*	*C. monspeliensis*	*C. parviflorus*	*C. populifolius*	*C. salviifolius*
Phenolic compounds											
**1-*O*-*β*-*D* -(6’-*O*-galloyl)-Glucopyranosyl-3-methoxy-5-hydroxybenzene**											(Danne et al., 1994; Tomás-Menor et al., 2013)
**1-*O*-*β*-*D*-Glucopyranosyl-3-methoxy-5-hydroxybenzene**											(Danne et al., 1994)
**3,4’-Dihydroxypropiophenone-3-*β*-*D*-glucoside**				(Barrajón-Catalán et al., 2011)		(Fernández-Arroyo et al., 2010; Barrajón-Catalán et al., 2011; Tomás-Menor et al., 2013)		(Barrajón-Catalán et al., 2011)			(Barrajón-Catalán et al., 2011; Tomás-Menor et al., 2013)
**3-*p*-Coumaroyl-quinic acid**							(Barrajón-Catalán et al., 2011)				
**5-*O*-*p*-Coumaroyl quinic acid methyl ester**							(Sadhu et al., 2006)				
**Benzyl benzoate**								(Loizzo et al., 2013)	(Angelopoulou et al., 2001b)		(Demetzos et al., 2002a; Loizzo et al., 2013)
**Benzyl salicilate**									(Angelopoulou et al., 2001b)		(Loizzo et al., 2013)
**Caffeoyl-quinic acid**					(Barrajón-Catalán et al., 2011)						
**Diisobutyl ester**								(Jemia et al., 2013)			
**Gentisoyl glucoside**				(Barrajón-Catalán et al., 2011; Tomás-Menor et al., 2013)		(Fernández-Arroyo et al., 2010; Tomás-Menor et al., 2013)	(Barrajón-Catalán et al., 2011)	(Barrajón-Catalán et al., 2011)		(Barrajón-Catalán et al., 2011)	

	C. albidus	*C. creticus* subsp. *creticus*	*C. creticus* subsp. *eriocephalus*	*C. clusii*	*C. crispus*	*C. ladanifer*	*C. laurifolius*	*C. monspeliensis*	*C. parviflorus*	*C. populifolius*	*C. salviifolius*
**Chlorogenic acid**							(Orhan et al., 2013)				
**Hydroxy-ferulic acid hexoside**								(Barrajón-Catalán et al., 2011)			
**Hydroxy-ferulic acid rhamnoside**	(Barrajón-Catalán et al., 2011)										
**Methyl salicylate**											(Loizzo et al., 2013)
**Myrciaphenone B**							(Barrajón-Catalán et al., 2011)				
**Uralenneoside**	(Barrajón-Catalán et al., 2011; Tomás-Menor et al., 2013)			(Barrajón-Catalán et al., 2011; Tomás-Menor et al., 2013)	(Barrajón-Catalán et al., 2011)	(Fernández-Arroyo et al., 2010; Tomás-Menor et al., 2013)	(Barrajón-Catalán et al., 2011)	(Barrajón-Catalán et al., 2011)			(Tomás-Menor et al., 2013)
**Vanillic acid *β*-glucoside**											(Tomás-Menor et al., 2013)
Tannins											
**Cornusiin B**						(Fernández-Arroyo et al., 2010; Barrajón-Catalán et al., 2011)				(Barrajón-Catalán et al., 2011)	(Barrajón-Catalán et al., 2011; Tomás-Menor et al., 2013)
**Ellagic acid**						(Fernández-Arroyo et al., 2010)	(Sadhu et al., 2006; Orhan et al., 2013)				
**Ellagic acid-7-xyloside**						(Fernández-Arroyo et al., 2010)				(Barrajón-Catalán et al., 2011)	(Barrajón-Catalán et al., 2011; Tomás-Menor et al., 2013)
**Gallic acid**	(Barrajón-Catalán et al., 2011)			(Barrajón-Catalán et al., 2011)	(Barrajón-Catalán et al., 2011)	(Fernández-Arroyo et al., 2010; Barrajón-Catalán et al., 2011)	(Barrajón-Catalán et al., 2011; Orhan et al., 2013)	(Barrajón-Catalán et al., 2011)		(Barrajón-Catalán et al., 2011)	(Barrajón-Catalán et al., 2011; Tomás-Menor et al., 2013)
**Glucogallin**	(Barrajón-Catalán et al., 2011)					(Fernández-Arroyo et al., 2010; Tomás-Menor et al., 2013)				(Barrajón-Catalán et al., 2011)	(Tomás-Menor et al., 2013)

	C. albidus	*C. creticus* subsp. *creticus*	*C. creticus* subsp. *eriocephalus*	*C. clusii*	*C. crispus*	*C. ladanifer*	*C. laurifolius*	*C. monspeliensis*	*C. parviflorus*	*C. populifolius*	*C. salviifolius*
**Hexahydroxydiphenoyl-*D*-glucose**						(Fernández-Arroyo et al., 2010)					(Tomás-Menor et al., 2013)
**Hexahydroxydiphenoyl-glucose**	(Barrajón-Catalán et al., 2011)			(Barrajón-Catalán et al., 2011)	(Barrajón-Catalán et al., 2011)	(Barrajón-Catalán et al., 2011);	(Barrajón-Catalán et al., 2011)	(Barrajón-Catalán et al., 2011)		(Barrajón-Catalán et al., 2011)	(Barrajón-Catalán et al., 2011)
**Pedunculagin**	(Barrajón-Catalán et al., 2011)					(Fernández-Arroyo et al., 2010)	(Barrajón-Catalán et al., 2011)				
**Punicalagin**				(Barrajón-Catalán et al., 2011)		(Fernández-Arroyo et al., 2010; Barrajón-Catalán et al., 2011; Tomás-Menor et al., 2013)	(Barrajón-Catalán et al., 2011)	(Barrajón-Catalán et al., 2011)		(Barrajón-Catalán et al., 2011)	(Barrajón-Catalán et al., 2011; Tomás-Menor et al., 2013)
**Punicalagin gallate**				(Barrajón-Catalán et al., 2011; Tomás-Menor et al., 2013)		(Barrajón-Catalán et al., 2011; Tomás-Menor et al., 2013)	(Barrajón-Catalán et al., 2011)			(Barrajón-Catalán et al., 2011)	(Barrajón-Catalán et al., 2011; Tomás-Menor et al., 2013)
**Punicalin**				(Barrajón-Catalán et al., 2011)		(Fernández-Arroyo et al., 2010; Barrajón-Catalán et al., 2011; Tomás-Menor et al., 2013)	(Barrajón-Catalán et al., 2011)	(Barrajón-Catalán et al., 2011)		(Barrajón-Catalán et al., 2011)	(Barrajón-Catalán et al., 2011; Tomás-Menor et al., 2013)
**Strictinin**						(Fernández-Arroyo et al., 2010)					
**HYDROCARBONS**											
**2-Phenyl-1,3-butadiene**								(Loizzo et al., 2013)			(Loizzo et al., 2013)
**Dodecane**								(Angelopoulou et al., 2001a)	(Angelopoulou et al., 2001b)		(Demetzos et al., 2002a)
**Heneicosane**									(Angelopoulou et al., 2001b)		(Demetzos et al., 2002a)
**Hentriacontane**								(Jemia et al., 2013)			(Loizzo et al., 2013)

	C. albidus	*C. creticus* subsp. *creticus*	*C. creticus* subsp. *eriocephalus*	*C. clusii*	*C. crispus*	*C. ladanifer*	*C. laurifolius*	*C. monspeliensis*	*C. parviflorus*	*C. populifolius*	*C. salviifolius*
**Heptacosane**								(Robles and Garzino, 2000; Angelopoulou et al., 2001a; Angelopoulou et al., 2002; Loizzo et al., 2013; Jemia et al., 2013)			(Loizzo et al., 2013)
***n*-Docosane**	(Llusià et al., 2010)							(Loizzo et al., 2013)	(Angelopoulou et al., 2001b)		(Demetzos et al., 2002a)
***n*-Heptadecene**									(Angelopoulou et al., 2001b)		(Demetzos et al., 2002a)
***n*-Hexadecane**	(Maccioni et al., 2007)							(Angelopoulou et al., 2001a; Angelopoulou et al., 2002)			
**Nonacosane**								(Robles and Garzino, 2000;Angelopoulou et al., 2001a; Jemia et al., 2013); Loizzo et al., 2013)			(Loizzo et al., 2013)
**Neophytadiene**								(Jemia et al., 2013)	(Angelopoulou et al., 2001b)		(Demetzos et al., 2002a; Loizzo et al., 2013)
***n*-Pentadecane**	(Maccioni et al., 2007)							(Angelopoulou et al., 2002)	(Angelopoulou et al., 2001b)		(Demetzos et al., 2002a)
***n*-Tetradecane**	(Maccioni et al., 2007)							(Angelopoulou et al., 2002)			
***n*-Tridecane**	(Maccioni et al., 2007)										
***n*-Undecane**	(Maccioni et al., 2007)										
**Octacosane**	(Llusià et al., 2010)							(Angelopoulou et al., 2002; Jemia et al., 2013)			
**Octadecane**								(Angelopoulou et al., 2002)			

	C. albidus	*C. creticus* subsp. *creticus*	*C. creticus* subsp. *eriocephalus*	*C. clusii*	*C. crispus*	*C. ladanifer*	*C. laurifolius*	*C. monspeliensis*	*C. parviflorus*	*C. populifolius*	*C. salviifolius*
**Pentacosane**								(Robles and Garzino, 2000; Angelopoulou et al., 2001a; Loizzo et al., 2013; Jemia et al., 2013)	(Angelopoulou et al., 2001b)		(Demetzos et al., 2002a; Loizzo et al., 2013)
**Pentadecene**								(Angelopoulou et al., 2002)			
**Squalene**								(Loizzo et al., 2013)			
**Tetracosane**	(Llusià et al., 2010)							(Angelopoulou et al., 2002)			(Loizzo et al., 2013)
**Tetradecene**								(Angelopoulou et al., 2002)	(Angelopoulou et al., 2001b)		(Demetzos et al., 2002a)
**Triacontane**								(Robles and Garzino, 2000)			
**Tricosane**	(Paolini et al., 2008)							(Robles and Garzino, 2000 Angelopoulou et al., 2002; Jemia et al., 2013; Loizzo et al., 2013)			(Demetzos et al., 2002a)
**Hexadecane**								(Angelopoulou et al., 2001a)			
**FATTY ACIDS**											
**(*Z*,*Z*)-9,12-Octadecadienoic acid**								(Jemia et al., 2013)			
**(*Z*,*Z*,*Z*)-9,12,15-Octadecatrienoic acid**								(Jemia et al., 2013)			
**(*Z*,*Z*,*Z*)-9,12,15-Octadecatrienoic acid methyl ester**											(Loizzo et al., 2013)
**1-Eicosanol (Arachidyl alcohol)**	(Llusià et al., 2010)										
**9-*cis*-Hexadecenoic acid (Palmitoleic acid)**	(Müller et al., 2013)										

	C. albidus	*C. creticus* subsp. *creticus*	*C. creticus* subsp. *eriocephalus*	*C. clusii*	*C. crispus*	*C. ladanifer*	*C. laurifolius*	*C. monspeliensis*	*C. parviflorus*	*C. populifolius*	*C. salviifolius*
***cis*-11,14,17-Eicosatrienoic acid**	(Müller et al., 2013)										
***cis*-11,14-Eicosadienoic acid**	(Müller et al., 2013)										
**Decanoic acid (Capric acid)**	(Müller et al., 2013)								(Angelopoulou et al., 2001b)		
**Docosanoic acid**								(Jemia et al., 2013)			
**Dodecanoic acid (Lauric acid)**	(Müller et al., 2013)							(Angelopoulou et al., 2001a)	(Angelopoulou et al., 2001b)		(Demetzos et al., 2002a)
**Eicosanoic acid (Arachidic acid)**	(Müller et al., 2013)							(Jemia et al., 2013)			
**Eicosenoic acid**	(Müller et al., 2013)										
**Ethyl linoleate**								(Robles and Garzino, 2000)			
**Ethyl myristate**								(Robles and Garzino, 2000)			
**Ethyl palmitate**								(Robles and Garzino, 2000)			
**Heneicosanoic acid (Heneicosylic acid)**	(Müller et al., 2013)										
**Heptacosanoic acid**								(Jemia et al., 2013)			
**Heptadecanoic acid (Margaric acid)**	(Müller et al., 2013)										
**Hexacosanoic acid**								(Jemia et al., 2013)			
**Hexadecanoic acid (Palmitic acid)**	(Paolini et al., 2008; Müller et al., 2013)	(Demetzos et al., 1994b)						(Robles and Garzino, 2000; Angelopoulou et al., 2002)	(Angelopoulou et al., 2001b)		(Demetzos et al., 2002a)

	C. albidus	*C. creticus* subsp. *creticus*	*C. creticus* subsp. *eriocephalus*	*C. clusii*	*C. crispus*	*C. ladanifer*	*C. laurifolius*	*C. monspeliensis*	*C. parviflorus*	*C. populifolius*	*C. salviifolius*
**Linoleic acid**	(Müller et al., 2013)										
**Linolelaidic acid**	(Müller et al., 2013)										
**Nonanoic acid**									(Angelopoulou et al., 2001b)		(Loizzo et al., 2013)
**Octadecanoic acid (Stearic acid)**	(Müller et al., 2013)							(Jemia et al., 2013)			
**Octanoic acid (Caprylic acid)**	(Müller et al., 2013)										
**Oleic acid**	(Müller et al., 2013)										
**Pentacosanoic acid**								(Jemia et al., 2013)			
**Pentadecanoic acid (Pentadecylic acid)**	(Llusià et al., 2010; Müller et al., 2013)							(Jemia et al., 2013)			
**Primaric acid**	(Llusià et al., 2010)										
**Tetracosanoic acid (Lignoceric acid)**	(Müller et al., 2013)							(Jemia et al., 2013)			
**Tetradecanoic acid (Myristic acid)**	(Llusià et al., 2010; Müller et al., 2013)							(Angelopoulou et al., 2001a; Robles and Garzino, 2000; Jemia et al., 2013 Loizzo et al., 2013)	(Angelopoulou et al., 2001b)		(Demetzos et al., 2002a)
**Tricosanoic acid (Tricosylic acid)**	(Müller et al., 2013)							(Jemia et al., 2013)			
**Tridecanoic acid (Tridecylic acid)**	(Müller et al., 2013)										
***α*-Linolenic acid**	(Müller et al., 2013)										
***γ*-Linolenic acid**	(Müller et al., 2013)										

	C. albidus	*C. creticus* subsp. *creticus*	*C. creticus* subsp. *eriocephalus*	*C. clusii*	*C. crispus*	*C. ladanifer*	*C. laurifolius*	*C. monspeliensis*	*C. parviflorus*	*C. populifolius*	*C. salviifolius*
**CARBONYLIC COMPOUNDS**											
**(*E*)- *β*-Damascenone**								(Loizzo et al., 2013)			(Loizzo et al., 2013)
**(*E*)-2-Hexenal**											(Loizzo et al., 2013)
**(*E*)-2-Hexylcinnamaldheyde**								(Loizzo et al., 2013)			
**(*E*)-Geranylacetone**											(Loizzo et al., 2013)
**(*E, E*)-2, 4-Decadienal**									(Angelopoulou et al., 2001b)		(Demetzos et al., 2002a)
**(*E,E*)-Farnesylacetone**											(Loizzo et al., 2013)
**(*Z*)-*β*-Damascenone**											(Loizzo et al., 2013)
**2,2,6-Trimethylcyclohexanone**						(Mariotti et al., 1997; Ramalho et al., 1999; Robles et al., 2003; Gomes et al., 2005; Teixeira et al., 2007; Zidane et al., 2013)					
**2,6,6-Trimethyl-2-cyclohexenone**						(Ramalho et al., 1999)					
**3-Nonen-2-one**								(Robles and Garzino, 2000)			
**4-Hydroxy-3-methylacetophenone**						(Ramalho et al., 1999)					
**4-*β*-D-Glucopyranosiloxyacetophenone**							(De Pascual Teresa et al., 1986)				
**5-Hydroxymethylfurfural**											(Loizzo et al., 2013)
**6-Methyl-5-hepten-2-one**	(Maccioni et al., 2007)										

	C. albidus	*C. creticus* subsp. *creticus*	*C. creticus* subsp. *eriocephalus*	*C. clusii*	*C. crispus*	*C. ladanifer*	*C. laurifolius*	*C. monspeliensis*	*C. parviflorus*	*C. populifolius*	*C. salviifolius*
**Acetophenone**						(Mariotti et al., 1997; Ramalho et al., 1999)					(Demetzos et al., 2002a)
**Benzaldehyde**						(Robles et al., 2003)					(Demetzos et al., 2002a)
**Dihydro-*α*-ionone**								(Angelopoulou et al., 2002)			
**Dihydro-*β*-ionone**								(Robles and Garzino, 2000; Angelopoulou et al., 2001a; Angelopoulou et al., 2002)			
**Dodecanal**	(Paolini et al., 2008)										(Loizzo et al., 2013)
**Hexahydrofarnesyl acetone**											(Loizzo et al., 2013)
***n*-Decanal**	(Maccioni et al., 2007; Paolini et al., 2008)								(Angelopoulou et al., 2001b)		(Demetzos et al., 2002a)
**Nonanal**	(Maccioni et al., 2007; Paolini et al., 2008)		(Paolini et al., 2009)					(Angelopoulou et al., 2001a; Loizzo et al., 2013)			(Demetzos et al., 2002a; Loizzo et al., 2013)
**Octanal**	(Maccioni et al., 2007)										
**Pentadecanal**											(Loizzo et al., 2013)
**Tetradecanal**	(Paolini et al., 2008)										(Loizzo et al., 2013)
**Tridecanal**											(Loizzo et al., 2013)
**Trimenal**									(Angelopoulou et al., 2001b)		(Demetzos et al., 2002a)
**Undec-10-en-al**									(Angelopoulou et al., 2001b)		(Demetzos et al., 2002a)
**Undec-9-en-al**									(Angelopoulou et al., 2001b)		
**Undecan-2-one**								(Loizzo et al., 2013); (Jemia et al., 2013)			
**Undecanal**	(Paolini et al., 2008)										(Loizzo et al., 2013)
**Vitispirane I**								(Loizzo et al., 2013)			

	C. albidus	*C. creticus* subsp. *creticus*	*C. creticus* subsp. *eriocephalus*	*C. clusii*	*C. crispus*	*C. ladanifer*	*C. laurifolius*	*C. monspeliensis*	*C. parviflorus*	*C. populifolius*	*C. salviifolius*
**Vitispirane II**											(Loizzo et al., 2013)
**α-Campholene aldehyde**						(Zidane et al., 2013)					
**α-Ionone**								(Robles and Garzino, 2000; Angelopoulou et al., 2001a; Angelopoulou et al., 2002;Loizzo et al., 2013)	(Angelopoulou et al., 2001b)		(Demetzos et al., 2002a; Loizzo et al., 2013)
**β-Ionone**			(Demetzos et al., 1997)					(Robles and Garzino, 2000; Angelopoulou et al., 2001a; Angelopoulou et al., 2002;Loizzo et al., 2013)	(Angelopoulou et al., 2001b)		(Demetzos et al., 2002a; Loizzo et al., 2013)
**PHYTOHORMONES & VITAMINS**											
**2-Isopentenyladenine**	(Müller et al., 2013)										
**Abscisic acid**	(Oñate and Munné-Bosch, 2010; (Müller et al., 2013)										
**Auxin [Indole-3-acetic acid]**	(Müller et al., 2013)										
**Dihydrozeatin**	(Müller et al., 2013)										
**Dihydrozeatin riboside**	(Müller et al., 2013)										
**Gibberellin A24**	(Müller et al., 2013)										
**Gibberellin A4**	(Müller et al., 2013)										

	C. albidus	*C. creticus* subsp. *creticus*	*C. creticus* subsp. *eriocephalus*	*C. clusii*	*C. crispus*	*C. ladanifer*	*C. laurifolius*	*C. monspeliensis*	*C. parviflorus*	*C. populifolius*	*C. salviifolius*
**Gibberellin A9**	(Müller et al., 2013)										
**Isopentenyladenosine**	(Müller et al., 2013)										
**Jasmonic acid**	(Oñate and Munné-Bosch, 2010; (Müller et al., 2013)										
**Salicylic acid**	(Müller et al., 2013)										
**Zeatin**	(Oñate and Munné-Bosch, 2010; (Müller et al., 2013)										
**Zeatin riboside**	(Oñate and Munné-Bosch, 2010; (Müller et al., 2013)										
***α*-Tocopherol**	(Müller et al., 2013)										
***α*-Tocotrienol**	(Müller et al., 2013)										
***β*-Tocopherol**	(Müller et al., 2013)										
***β*-Tocotrienol**	(Müller et al., 2013)										
***γ*-Tocopherol**	(Müller et al., 2013)										

	C. albidus	*C. creticus* subsp. *creticus*	*C. creticus* subsp. *eriocephalus*	*C. clusii*	*C. crispus*	*C. ladanifer*	*C. laurifolius*	*C. monspeliensis*	*C. parviflorus*	*C. populifolius*	*C. salviifolius*
**OTHERS**											
***β*-Sitosterol-3-*O*-*β*-glucoside**							(Sadhu et al., 2006)				
**Hexadecanol**								(Angelopoulou et al., 2002)	(Angelopoulou et al., 2001b)		(Demetzos et al., 2002a)
**Hexanol**	(Paolini et al., 2008)		(Paolini et al., 2009)								
**(*E*)-2-Hexen-1-ol**	(Maccioni et al., 2007)										
**(*E*)-2-Hexenyl acetate**	(Maccioni et al., 2007)										
**(*E*)-3-Hexen-1-ol**	(Maccioni et al., 2007)										
**(*E*)-3-Hexenyl acetate**	(Maccioni et al., 2007)										
**(E)-Ethyl cinnamate**											(Demetzos et al., 2002a)
**(*Z*)-3-Hexenyl benzoate**									(Angelopoulou et al., 2001b)		(Demetzos et al., 2002a)
**(*Z*)-3-Hexenyl butyrate**	(Maccioni et al., 2007)										
**(*Z*)-3-Hexenyl isovalerate**	(Maccioni et al., 2007)										
**(Z)-Ethyl cinnamate**											(Demetzos et al., 2002a)
**(Z)-Hex-2- en-1-ol**	(Paolini et al., 2008)		(Paolini et al., 2009)								
**(Z)-Hex-3-en-1-ol**	(Paolini et al., 2008)		(Paolini et al., 2009)								
**(Z)-Octadec-9-en-1-ol**											(Loizzo et al., 2013)
**1-(4-Hydroxy-3-methoxyphenyl)-2-[4-(3-*α*-L-rhamnopyranoxypropyl)-2-methoxyphenoxy]-1,3-propanediol**							(Sadhu et al., 2006)				

	C. albidus	*C. creticus* subsp. *creticus*	*C. creticus* subsp. *eriocephalus*	*C. clusii*	*C. crispus*	*C. ladanifer*	*C. laurifolius*	*C. monspeliensis*	*C. parviflorus*	*C. populifolius*	*C. salviifolius*
**1,3-Diacetoxy-5-(tetra-*O*-acetyl-*β*-D-glucopyranosiloxy)-benzene**							(De Pascual Teresa et al., 1986)				
**1,3-Dihydroxy-5-*β*-D-glucopyranosiloxybenzene**							(De Pascual Teresa et al., 1986)				
**1-*O*-Methyl-*epi*-inositol**							(De Pascual Teresa et al., 1986)				
**2,3-Dihydro-2-(4'-*α*-L-rhamnopyranosyloxy-3'-methoxyphenyl)-3-hydroxymethyl-7-methoxy-5-benzofuranpropanol**							(Sadhu et al., 2006)				
**2,3-Dihydrobenzofuran**											(Loizzo et al., 2013)
**2-Cyclohexene-1 methanol [2,6] dimethyl-6(4 methyl-3pentenyl)**									(Demetzos et al., 1990b)		
**2-Nonanol**											(Demetzos et al., 2002a)
**3-Hexen-1-ol**						(Ramalho et al., 1999)					
**Arabonic acid**	(Tomás-Menor et al., 2013)										
**Ascorbic acid glycoside**											(Tomás-Menor et al., 2013)
**Benzyl salicylate**									(Angelopoulou et al., 2001b)		(Demetzos et al., 2002a)
**Berchemol 9-*O*-rhamnoside**							(Sadhu et al., 2006)				
**Betuloside**				(Tomás-Menor et al., 2013)		(Tomás-Menor et al., 2013)					(Danne et al., 1994; Tomás-Menor et al., 2013)

	C. albidus	*C. creticus* subsp. *creticus*	*C. creticus* subsp. *eriocephalus*	*C. clusii*	*C. crispus*	*C. ladanifer*	*C. laurifolius*	*C. monspeliensis*	*C. parviflorus*	*C. populifolius*	*C. salviifolius*
**Bis (2-ethylhexyl) phthalate**						(Zidane et al., 2013)					
**Cuminic alcohol (*p*-Cymen-7-ol)**									(Demetzos et al., 1990b)		
**Cyercene**						(Zidane et al., 2013)					
**Daidzin 6''-*O*-acetate**											(Tomás-Menor et al., 2013)
**Diethyl phthalate**						(Zidane et al., 2013)					
**Digaloil-*β*-D-glucopiranose**						(Fernández-Arroyo et al., 2010)					
**Dihydroctinidiolide**								(Jemia et al., 2013)			
**Dihydrodehydrodiconiferyl alcohol 9'-*O*-*α*-L-rhamnoside**						(Fernández-Arroyo et al., 2010)	(Sadhu et al., 2006)				
**Ducheside A**						(Fernández-Arroyo et al., 2010)					
**Ethyl hexadecanoate**											(Demetzos et al., 2002a)
**Ethylbenzoate**											(Demetzos et al., 2002a)
**Geranyl tiglate**									(Angelopoulou et al., 2001b)		(Demetzos et al., 2002a)
**Hydroxydihydroedulan**											(Loizzo et al., 2013)
**Isosibiricin**											(Tomás-Menor et al., 2013)
**Lyoniresinol-3*α*-*O*-L-rhamnoside**											(Tomás-Menor et al., 2013)
**Methyl hexadecanoate**									(Angelopoulou et al., 2001b)		(Demetzos et al., 2002a)
**Methyl octadecanoate**									(Angelopoulou et al., 2001b)		(Demetzos et al., 2002a)
**Mirciaphenone B**						(Fernández-Arroyo et al., 2010)					
**n-Nonanol**											(Demetzos et al., 2002a)

	C. albidus	*C. creticus* subsp. *creticus*	*C. creticus* subsp. *eriocephalus*	*C. clusii*	*C. crispus*	*C. ladanifer*	*C. laurifolius*	*C. monspeliensis*	*C. parviflorus*	*C. populifolius*	*C. salviifolius*
***n*-Tetradecanol (Myristyl alcohol)**									(Angelopoulou et al., 2001b)		(Demetzos et al., 2002a)
**Octadecanol**											(Demetzos et al., 2002a; Loizzo et al., 2013)
**Olivil-9-*O*-*β*-D-xyloside**							(Sadhu et al., 2006)				
**Prodelphinidin B2, 3'-*O*-gallate**											(Tomás-Menor et al., 2013)
**Quinic acid**	(Tomás-Menor et al., 2013)			(Tomás-Menor et al., 2013)		(Fernández-Arroyo et al., 2010; Tomás-Menor et al., 2013)					(Tomás-Menor et al., 2013)
**Roseoside**							(De Pascual Teresa et al., 1986)				
**Sabinene hydrate acetate**									(Angelopoulou et al., 2001b)		(Demetzos et al., 2002a)
**Sabinyl acetate**									(Demetzos et al., 1990b)		
**Secoisolariciresinol *β*-D-glucoside**											(Tomás-Menor et al., 2013)
**Shikimic acid**						(Fernández-Arroyo et al., 2010; Tomás-Menor et al., 2013)					(Tomás-Menor et al., 2013)
**Tridecanol**											(Loizzo et al., 2013)
**Vomifoliol *β*-D-glucopyranoside**											(Tomás-Menor et al., 2013)
***β*-D-Glucopyranosiloxyethane**							(De Pascual Teresa et al., 1986)				

REFERENCES

Akkol, E. K., Orhan, I. E., and Yeşilada, E. (2012). Anticholinesterase and antioxidant effects of the ethanol extract, ethanol fractions and isolated flavonoids from *Cistus laurifolius* L. leaves. *Food Chem.* 131, 626–631. doi:10.1016/j.foodchem.2011.09.041.

Anastasaki, T., Demetzos, C., Perdetzoglou, D., Gazouli, M., Loukis, A., and Harvala, C. (1999). Analysis of labdane-type diterpenes from *Cistus creticus* (subsp. *creticus* and subsp. *eriocephalus*), by GC and GC-MS. *Planta Med.* 65, 735–739. doi:10.1055/s-1999-14095.

Angelopoulou, D., Demetzos, C., Dimas, C., Perdetzoglou, D., and Loukis, A. (2001a). Essential oils and hexane extracts from leaves and fruits of *Cistus monspeliensis*. Cytotoxic activity of ent-13-epi-manoyl oxide and its isomers. *Planta Med.* 67, 168–171. doi:10.1055/s-2001-11497.

Angelopoulou, D., Demetzos, C., and Perdetzoglou, D. (2001b). An interpopulation study of the essential oils of *Cistus parviflorus* L. growing in Crete (Greece). *Biochem. Syst. Ecol.* 29, 405–415. doi:10.1016/S0305-1978(00)00071-5.

Angelopoulou, D., Demetzos, C., and Perdetzoglou, D. (2002). Diurnal and seasonal variation of the essential oil labdanes and clerodanes from *Cistus monspeliensis* L. leaves. *Biochem. Syst. Ecol.* 30, 189–203. doi:10.1016/S0305-1978(01)00074-6.

Barrajón-Catalán, E., Fernández-Arroyo, S., Roldán, C., Guillén, E., Saura, D., Segura-Carretero, A., and Micol, V. (2011). A systematic study of the polyphenolic composition of aqueous extracts deriving from several *Cistus* genus species: evolutionary relationship. *Phytochem. Anal.* 22, 303–312. doi:10.1002/pca.1281.

Berti, G., Livi, O., and Segnini, D. (1970). Cistodiol and cistodioic acid, diterpenoids with a cis-fused clerodane skeleton. *Tetrahedron Lett.* 11, 1401–1404. doi:10.1016/S0040-4039(01)97980-8.

Berti, G., Livi, O., Segnini, D., and Cavero, I. (1967). Determination of constitution and synthesis of a new flavone from *Cistus monspeliensis* L. *Tetrahedron* 23, 2295–2300. doi:10.1016/0040-4020(67)80066-8.

Danne, A., Petereit, F., and Nahrstedt, A. (1994). Flavan-3-ols, prodelphinidins and further polyphenols from *Cistus salvifolius*. *Phytochemistry* 37, 533–538.

Demetzos, C., Angelopoulou, D., Kolocouris, A., Daliani, I., and Mavromoustakos, T. (2001a). Structure elucidation, conformational analysis and thermal effects on membrane bilayers of an antimicrobial myricetin ether derivative. *J. Heterocycl. Chem.* 38, 703–710. doi:10.1002/jhet.5570380327.

Demetzos, C., Angelopoulou, D., and Perdetzoglou, D. (2002a). A comparative study of the essential oils of *Cistus salviifolius* in several populations of Crete (Greece). *Biochem. Syst. Ecol.* 30, 651–665. doi:10.1016/S0305-1978(01)00145-4.

Demetzos, C., Dimas, K., Hatziantoniou, S., Anastasaki, T., and Angelopoulou, D. (2001b). Cytotoxic and anti-inflammatory activity of labdane and cis-clerodane type diterpenes. *Planta Med.* 67, 614–618. doi:10.1055/s-2001-17362.

Demetzos, C., Harvala, C., Philianos, S. M., and Skaltsounis, A. L. (1990a). A new labdane-type diterpene and other compounds from the leaves of *Cistus incanus* ssp. *creticus*. *J Nat Prod* 53, 1365–1368. doi:10.1021/np50071a039.

Demetzos, C., Katerinopoulos, H., Kouvarakis, A., Stratigakis, N., Loukis, A., Ekonomakis, C., Spiliotis, V., and Tsaknis, J. (1997). Composition and antimicrobial activity of the essential oil of *Cistus creticus* subsp. *eriocephalus*. *Planta Med.* 63, 477–479. doi:10.1055/s-2006-957742.

Demetzos, C., Kolocouris, A., and Anastasaki, T. (2002b). A simple and rapid method for the differentiation of C-13 manoyl oxide epimers in biologically important samples using GC-MS analysis supported with NMR spectroscopy and computational chemistry results. *Bioorg. Med. Chem. Lett.* 12, 3605–3609.

Demetzos, C., Loukis, A., Spiliotis, V., Zoakis, N., Stratigakis, N., and Katerinopoulos, H. E. (1995). Composition and Antimicrobial Activity of the Essential oil of *Cistus creticus* L. *J. Essent. Oil Res.* 7, 407–410. doi:10.1080/10412905.1995.9698549.

Demetzos, C., Mitaku, S., Couladis, M., Harvala, C., and Kokkinopoulos, D. (1994a). Natural metabolites of ent-13-epi-manoyl oxide and other cytotoxic diterpenes from the resin “LADANO” of *Cistus creticus*. *Panta Medica* 60, 590–591.

Demetzos, C., Mitaku, S., Hotellier, F., and Harvala, A. (1989). Polyphenolic glycosides from *Cistus creticus* L. leaves. *Ann. Pharm. Fr.* 47, 314–318.

Demetzos, C., Mitaku, S., Loukis, A., Harvala, C., and Gaily, A. (1994b). A New Drimane Sesquiterpene, Isomers of Manoyl Oxide and Other Volatile Constituents from the Resin “Ladano” of *Cistus incanus* subsp. *creticus* (L.) Heywood. *J. Essent. Oil Res.* 6, 37–41. doi:10.1080/10412905.1994.9698322.

Demetzos, C., Mitaku, S., Skaltsounis, A. L., Catherine Harvala, M. C., and Libot, F. (1994c). Diterpene esters of malonic acid from the resin “Ladano” of *Cistus creticus*. *Phytochemistry* 35, 979–981. doi:10.1016/S0031-9422(00)90651-4.

Demetzos, C. N., Chinou, J. B., Charvala, C. E., and Homatidou, V. I. (1990b). The essential oil of *Cistus parviflorus* and its antimicrobial activity in comparison with *C. monspeliensis*. *Fitoterapia* 61, 439–442.

Demetzos, C., Stahl, B., Anastassaki, T., Gazouli, M., Tzouvelekis, L., and Rallis, M. (1999). Chemical analysis and antimicrobial activity of the resin ladano, of its essential oil and of the isolated compounds. *Planta Med.* 65, 76–78. doi:10.1055/s-2006-960444.

Falara, V., Pichersky, E., and Kanellis, A. K. (2010). A copal-8-ol diphosphate synthase from the angiosperm *Cistus creticus* subsp. *creticus* Is a putative key enzyme for the formation of pharmacologically active, oxygen-containing labdane-type diterpenes. *Plant Physiol.* 154, 301 –310. doi:10.1104/pp.110.159566.

Fernández-Arroyo, S., Barrajón-Catalán, E., Micol, V., Segura-Carretero, A., and Fernández-Gutiérrez, A. (2010). High-performance liquid chromatography with diode array detection coupled to electrospray time-of-flight and ion-trap tandem mass spectrometry to identify phenolic compounds from a *Cistus ladanifer* aqueous extract. *Phytochem. Anal.* 21, 307–313. doi:10.1002/pca.1200.

Gomes, P. B., Mata, V. G., and Rodrigues, A. E. (2005). Characterization of the Portuguese-grown *Cistus ladanifer* essential oil. *J. Essent. Oil Res.* 17, 160–165. doi:10.1080/10412905.2005.9698864.

Hernández, I., Alegre, L., and Munné-Bosch, S. (2004). Drought-induced changes in flavonoids and other low molecular weight antioxidants in *Cistus clusii* grown under Mediterranean field conditions. *Tree Physiol.* 24, 1303–1311. doi:10.1093/treephys/24.11.1303.

Hernández, I., Alegre, L., and Munné-Bosch, S. (2011). Plant aging and excess light enhance flavan-3-ol content in *Cistus clusii*. *J. Plant Physiol.* 168, 96–102. doi:10.1016/j.jplph.2010.06.026.

Jemia, M. B., Kchouk, M. E., Senatore, F., Autore, G., Marzocco, S., Feo, V. D., and Bruno, M. (2013). Antiproliferative activity of hexane extract from Tunisian *Cistus libanotis*, *Cistus monspeliensis* and *Cistus villosus*. *Chem. Cent. J.* 7, 1–7. doi:10.1186/1752-153X-7-47.

Kalpoutzakis, E., Aligiannis, N., Skaltsounis, A.-L., and Mitakou, S. (2003). Cis-clerodane type diterpenes from *Cistus monspeliensis*. *J. Nat. Prod.* 66, 316–319. doi:10.1021/np0204388.

Kolocouris, A., Mavromoustakos, T., Demetzos, C., Terzis, A., and Grdadolnik, S. G. (2001). Structure elucidation and conformational properties of a novel bioactive clerodane diterpene using a combination of high field NMR spectroscopy, computational analysis and X-ray diffraction. *Bioorg. Med. Chem. Lett.* 11, 837–840. doi:10.1016/S0960-894X(01)00072-5.

Llusià, J., Peñuelas, J., Ogaya, R., and Alessio, G. (2010). Annual and seasonal changes in foliar terpene content and emission rates in *Cistus albidus* L. submitted to soil drought in Prades forest (Catalonia, NE Spain). *Acta Physiol. Plant.* 32, 387–394. doi:10.1007/s11738-009-0416-y.

Loizzo, M. R., Ben Jemia, M., Senatore, F., Bruno, M., Menichini, F., and Tundis, R. (2013). Chemistry and functional properties in prevention of neurodegenerative disorders of five *Cistus* species essential oils. *Food Chem. Toxicol.* 59, 586–594. doi:10.1016/j.fct.2013.06.040.

Maccioni, S., Baldini, R., Cioni, P. L., Tebano, M., and Flamini, G. (2007). In vivo volatiles emission and essential oils from different organs and pollen of *Cistus albidus* from Caprione (Eastern Liguria, Italy). *Flavour Fragr. J.* 22, 61–65. doi:10.1002/ffj.1759.

Mariotti, J. P., Tomi, F., Casanova, J., Costa, J., and Bernardini, A. F. (1997). Composition of the essential oil of *Cistus ladaniferus* L. cultivated in Corsica (France). *Flavour Fragr. J.* 12, 147–151. doi:10.1002/(SICI)1099-1026(199705)12:3<147::AID-FFJ631>3.0.CO;2-Q.

Müller, M., Siles, L., Cela, J., and Munné-Bosch, S. (2013). Perennially young: seed production and quality in controlled and natural populations of *Cistus albidus* reveal compensatory mechanisms that prevent senescence in terms of seed yield and viability. *J. Exp. Bot.*, ert372. doi:10.1093/jxb/ert372.

Oñate, M., and Munné-Bosch, S. (2010). Loss of flower bud vigour in the Mediterranean shrub, *Cistus albidus* L. at advanced developmental stages. *Plant Biol.* 12, 475–483. doi:10.1111/j.1438-8677.2009.00246.x.

Orhan, N., Aslan, M., Süküroğlu, M., and Deliorman Orhan, D. (2013). In vivo and in vitro antidiabetic effect of *Cistus laurifolius* L. and detection of major phenolic compounds by UPLC-TOF-MS analysis. *J. Ethnopharmacol.* 146, 859–865. doi:10.1016/j.jep.2013.02.016.

Ormeño, E., Fernandez, C., and Mévy, J.-P. (2007). Plant coexistence alters terpene emission and content of Mediterranean species. *Phytochemistry* 68, 840–852. doi:10.1016/j.phytochem.2006.11.033.

Paolini, J., Falchi, A., Quilichini, Y., Desjobert, J.-M., Cian, M.-C. D., Varesi, L., and Costa, J. (2009). Morphological, chemical and genetic differentiation of two subspecies of *Cistus creticus* L. (*C. creticus* subsp. *eriocephalus* and *C. creticus* subsp. *corsicus*). *Phytochemistry* 70, 1146–1160. doi:10.1016/j.phytochem.2009.06.013.

Paolini, J., Tomi, P., Bernardini, A.-F., Bradesi, P., Casanova, J., and Kaloustian, J. (2008). Detailed analysis of the essential oil from *Cistus albidus* L. by combination of GC/RI, GC/MS and 13C-NMR spectroscopy. *Nat. Prod. Res.* 22, 1270–1278. doi:10.1080/14786410701766083.

De Pascual Teresa, J., Urones, J. G., Marcos, I. S., Barcala, P. B., and Garrido, N. M. (1986). Diterpenoid and other components of *Cistus laurifolius*. *Phytochemistry* 25, 1185–1187. doi:10.1016/S0031-9422(00)81577-0.

De Pascual Teresa, J., Urones, J. G., Marcos, I. S., Bermejo, F., and Basabe, P. (1983). A rearranged labdane: Salmantic acid from *Cistus laurifolius*. *Phytochemistry* 22, 2783–2785. doi:10.1016/S0031-9422(00)97696-9.

Pomponio, R., Gotti, R., Santagati, N. A., and Cavrini, V. (2003). Analysis of catechins in extracts of *Cistus* species by microemulsion electrokinetic chromatography. *J. Chromatogr. A* 990, 215–223. doi:10.1016/S0021-9673(02)02010-1.

Qa’dan, F., Petereit, F., Mansoor, K., and Nahrstedt, A. (2006). Antioxidant oligomeric proanthocyanidins from *Cistus salvifolius*. *Nat. Prod. Res.* 20, 1216–1224. doi:10.1080/14786410600899225.

Qa’dan, F., Petereit, F., and Nahrstedt, A. (2003). Prodelphinidin trimers and characterization of a proanthocyanidin oligomer from *Cistus albidus*. *Pharm. - Int. J. Pharm. Sci.* 58, 416–419.

Ramalho, P. S., de Freitas, V. A. P., Macedo, A., Silva, G., and Silva, A. M. S. (1999). Volatile components of *Cistus ladanifer* leaves. *Flavour Fragr. J.* 14, 300–302. doi:10.1002/(SICI)1099-1026(199909/10)14:5<300::AID-FFJ830>3.0.CO;2-X.

Rivoal, A., Fernandez, C., Lavoir, A.-V., Olivier, R., Lecareux, C., Greff, S., Roche, P., and Vila, B. (2010). Environmental control of terpene emissions from *Cistus monspeliensis* L. in natural Mediterranean shrublands. *Chemosphere* 78, 942–949. doi:10.1016/j.chemosphere.2009.12.047.

Robles, C., Bousquet-Melou, A., Garzino, S., and Bonin, G. (2003). Comparison of essential oil composition of two varieties of *Cistus ladanifer*. *Biochem. Syst. Ecol.* 31, 339–343.

Robles, C., and Garzino, S. (2000). Infraspecific variability in the essential oil composition of *Cistus monspeliensis* leaves. *Phytochemistry* 53, 71–75. doi:10.1016/S0031-9422(99)00460-4.

Sadhu, S. K., Okuyama, E., Fujimoto, H., Ishibashi, M., and Yesilada, E. (2006). Prostaglandin inhibitory and antioxidant components of *Cistus laurifolius*, a Turkish medicinal plant. *J. Ethnopharmacol.* 108, 371–378. doi:10.1016/j.jep.2006.05.024.

Saracini, E., Tattini, M., Traversi, M. L., Vincieri, F. F., and Pinelli, P. (2005). Simultaneous LC-DAD and LC-MS determination of ellagitannins, flavonoid glycosides, and acyl-lycosyl flavonoids in *Cistus salvifolius* L. leaves. *Chromatographia* 62, 245–249. doi:10.1365/s10337-005-0623-7.

Teixeira, S., Mendes, A., Alves, A., and Santos, L. (2007). Simultaneous distillation–extraction of high-value volatile compounds from *Cistus ladanifer* L. *Anal. Chim. Acta* 584, 439–446. doi:10.1016/j.aca.2006.11.054.

Tomás-Lorente, F., Garcia-Grau, M. M., Nieto, J. L., and Tomás-Barberán, F. A. (1992). Flavonoids from *Cistus ladanifer* bee pollen. *Phytochemistry* 31, 2027–2029. doi:10.1016/0031-9422(92)80355-I.

Tomás-Menor, L., Morales-Soto, A., Barrajón-Catalán, E., Roldán-Segura, C., Segura-Carretero, A., and Micol, V. (2013). Correlation between the antibacterial activity and the composition of extracts derived from various Spanish *Cistus* species. *Food Chem. Toxicol.* 55, 313–322. doi:10.1016/j.fct.2013.01.006.

Urones, J. G., Basabe, P., Lithgow, A. M., Marcos, I. S., Jiménez, A., Díez, D., Gómez, A., White, A. J. P., Williams, D. J., Simmonds, M. S. J., et al. (1995a). New antifeedant neo-clerodane triol. Semisynthesis and antifeedant activity of neo-clerodane diterpenoids. *Tetrahedron* 51, 2117–2128. doi:10.1016/0040-4020(94)01085-E.

Urones, J. G., Basabe, P., Marcos, I. S., Jiménez, A., Lithgow, A. M., López, M., Moro, R., and Gómez, A. (1994). Ring a functionalized neo-clerodane diterpenoids from *Cistus populifolius*. *Tetrahedron* 50, 10791–10802. doi:10.1016/S0040-4020(01)89271-1.

Urones, J. G., Marcos, I. S., Basabe, P., Jimenez, A., Gomez, A., and Lithgow, A. M. (1995b). 2α,3β-dihydroxy-4(18)-neo-cleroden-15-oic acid from *Cistus populifolius*. *Phytochemistry* 38, 443–445. doi:10.1016/0031-9422(94)00709-3.

Vogt, T., and Gerhard Gul, P. (1994). Accumulation of flavonoids during leaf development in *Cistus laurifolius*. *Phytochemistry* 36, 591–597. doi:10.1016/S0031-9422(00)89780-0.

Vogt, T., Gülz, P.-G., and Wray, V. (1988). Epicuticular 5-O-methyl flavonols from *Cistus laurifolius*. *Phytochemistry* 27, 3712–3713. doi:10.1016/0031-9422(88)80812-4.

Vogt, T., Proksch, P., Gülz, P. G., and Wollenweber, E. (1987). Rare 6- and 8-O-methylated epicuticular flavonols from two *Cistus* species. *Phytochemistry* 26, 1027–1030. doi:10.1016/S0031-9422(00)82342-0.

Zidane, H., Elmiz, M., Aouinti, F., Tahani, A., Wathele, J., Sindic, M., and Elbachiri, A. (2013). Chemical composition and antioxidant activity of essential oil, various organic extracts of *Cistus ladanifer* and *Cistus libanotis* growing in Eastern Morocco. *Afr. J. Biotechnol.* 12, 5314–5320. doi:10.5897/AJB2013.12868.
