# Supplementary material for: Genus Cistus: a model for exploring labdane-type diterpenes' biosynthesis and a natural source of high value products with biological, aromatic, and pharmacological properties
Source: Front Chem. 2014 Jun 11;2:35. doi: 10.3389/fchem.2014.00035 (PMC4052220; doi:10.3389/fchem.2014.00035)
Supplement: Supplementary file 1 [file DataSheet1.ZIP › legends Supp Mat.docx]

**TABLES**

**Supplementary Table S1.** Distribution of the main 10 species of genus *Cistus* in the Mediterranean and around the Mediterranean region

**Supplementary Table S2.** The specific botanical characteristics of the main 10 species of genus *Cistus*: color of the flower petal, purple (P) or white (W); number of sepals (3 or 5); number of fruit compartments containing seeds (5 or 6-12); type of leaf base, petiolate (P) or sessile (S) and size of the style (XS, S, M, L) compared with flower stamens (Modified from Guzmán and Vargas, 2005).

**Supplementary Table S3**. Total number of secondary metabolites detected in the 10 *Cistus* species covered in this Review

**Supplementary Table S4.** Chemical composition of aerial parts and essential oils of *Cistus* species

**FIGURES**

**Figure S1.** Terpenoid biosynthesis: enzymes and metabolic intermediates. Mevalonic acid (MVA) pathway is cytosol localized and the 2-C-methyl-d-erythritol 4-phosphate (MEP) pathway is plastidial. The enzymes participating in these pathways are indicated in boxes: AACT, acetoacetyl CoA thiolase; HMGS, hydroxymethylglutaryl coenzyme A synthase; HMGR, hydroxymethylglutaryl coenzyme A reductase; MVK, mevalonate kinase; PMK, mevalonate 5-phosphate kinase; PMD, 5-diphosphomevalonate decarboxylase; IPPI, isopentenyl diphosphate isomerase; FPS, farnesyl diphosphate synthase; DXPS, deoxyxylulose 5-phosphate synthase; DXR, deoxyxylulose 5-phosphate reductoisomerase; CMS, 4-diphosphocytidyl-methylerythritol synthase; CMK, 4-diphosphocytidyl-methylerythritol kinase; MCS, methylerythritol 2,4-cyclodiphosphate synthase; HDS, hydroxymethylbutenyl 4-diphosphate synthase; HDR, hydroxymethylbutenyl 4-diphosphate reductase; GPS, geranyl diphosphate synthase; GGPPS, geranylgeranyl diphosphate synthase.

**Figure S2.** The phenylpropanoid pathway in *Cistus*. Single biosynthetic step is indicated by continuous arrow line and multiple steps by dotted arrow line. Thin lines indicate categories of metabolites synthesized in *Cistus.* Compounds with important biological functions synthesized in *Cistus* are included. PAL, phenylalanine ammonia-lyase; C4H, cinnamate 4-hydroxylase; 4CL, 4-coumarate CoA-ligase.
