# Supplementary material for: Genus Cistus: a model for exploring labdane-type diterpenes' biosynthesis and a natural source of high value products with biological, aromatic, and pharmacological properties
Source: Front Chem. 2014 Jun 11;2:35. doi: 10.3389/fchem.2014.00035 (PMC4052220; doi:10.3389/fchem.2014.00035)
Supplement: Supplementary file 1 [file DataSheet1.ZIP › Supp Table 1.PDF]

Table S1. Distribution of the main 10 species of genus *Cistus* in the Mediterranean and other neighbour regions.

Species / Area	***C. albidus***	***C. clusii***	***C. creticus***	***C. crispus***	***C. ladanifer***	***C. laurifolius***	***C. monspeliesis***	***C. parviflorus***	***C. populifolius***	***C. salviifolius***
Aegean Islands			+				+			+
Albania			+				+			+
Algeria	+	+	+	+	+		+			+
Andorra						+				
Bosnia-Herzegovina			+							
Bulgaria			+							+
Corsica	+		+	+		+	+			+
Crete			+				+			+
Croatia							+			
Cyprus			+		+		+	+		+
France	+			+	+	+	+		+	+
FYROM			+							
Gibraltar	+		+							+
Greece			+				+	+		+
Israel										+
Italy	+	+	+	+		+	+	+		+
Libya			+					+		
Madeira							+			
Malta			+				+			
Morocco	+	+	+	+	+	+			+	+
Portugal	+	+		+	+	+			+	+
Sardinia	+		+				+			
Sicily		+		+						
Spain	+	+	+	+	+	+	+		+	+
The Balearic Islands	+	+								
The Canary Islands					+		+			
Tunisia		+	+	+			+			+
Turkey			+			+		+		+

The information is from our observations and from the on line Distribution Map at University of Reading http://www.herbarium.rdg.ac.uk/mediplants/query.asp
