# Supplementary figures and images for: Genus Cistus: a model for exploring labdane-type diterpenes' biosynthesis and a natural source of high value products with biological, aromatic, and pharmacological properties
Source: Front Chem. 2014 Jun 11;2:35. doi: 10.3389/fchem.2014.00035 (PMC4052220; doi:10.3389/fchem.2014.00035)

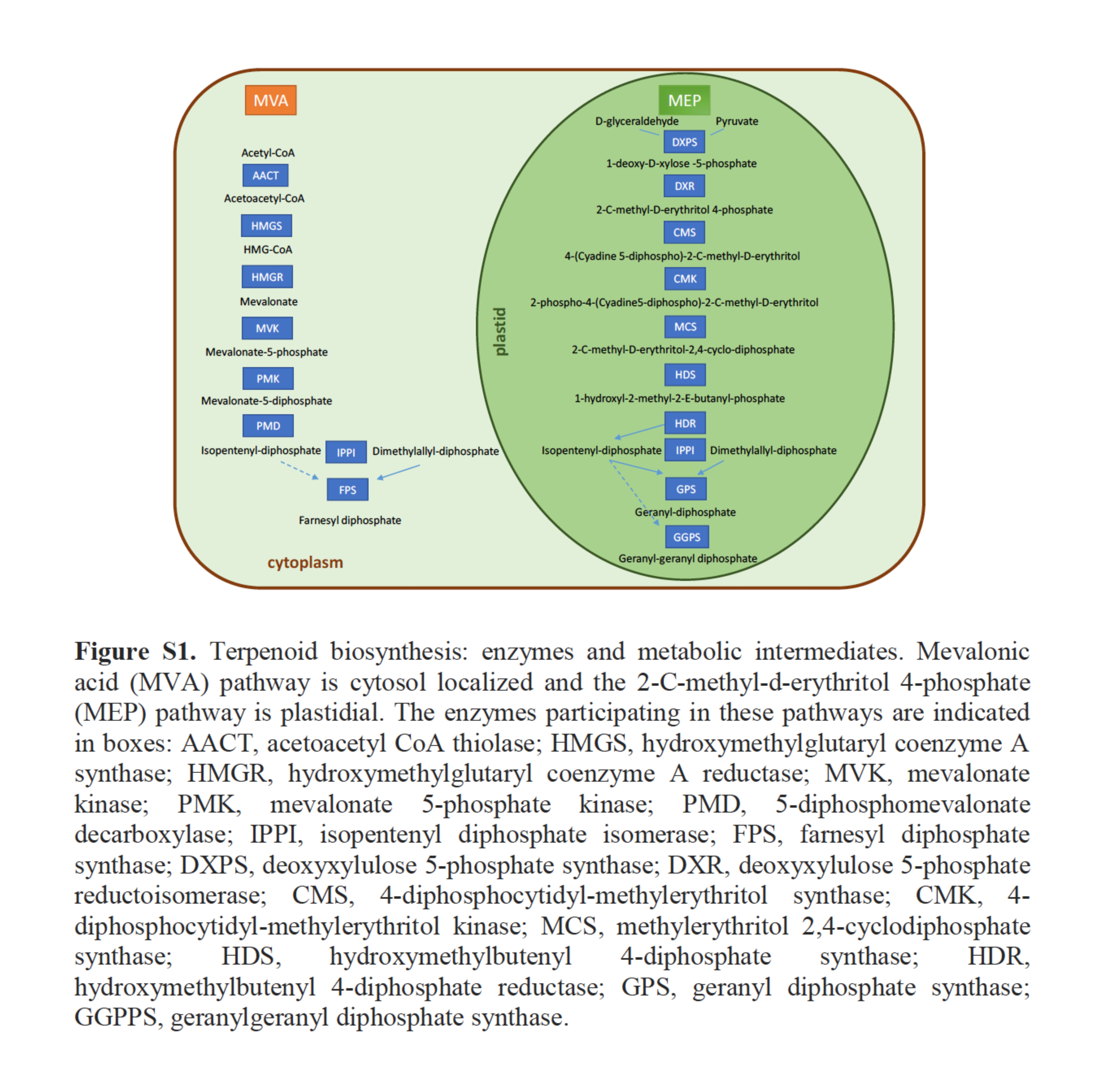

Supplement: Supplementary file 1 [file DataSheet1.ZIP › Supp Fig 1.JPEG]

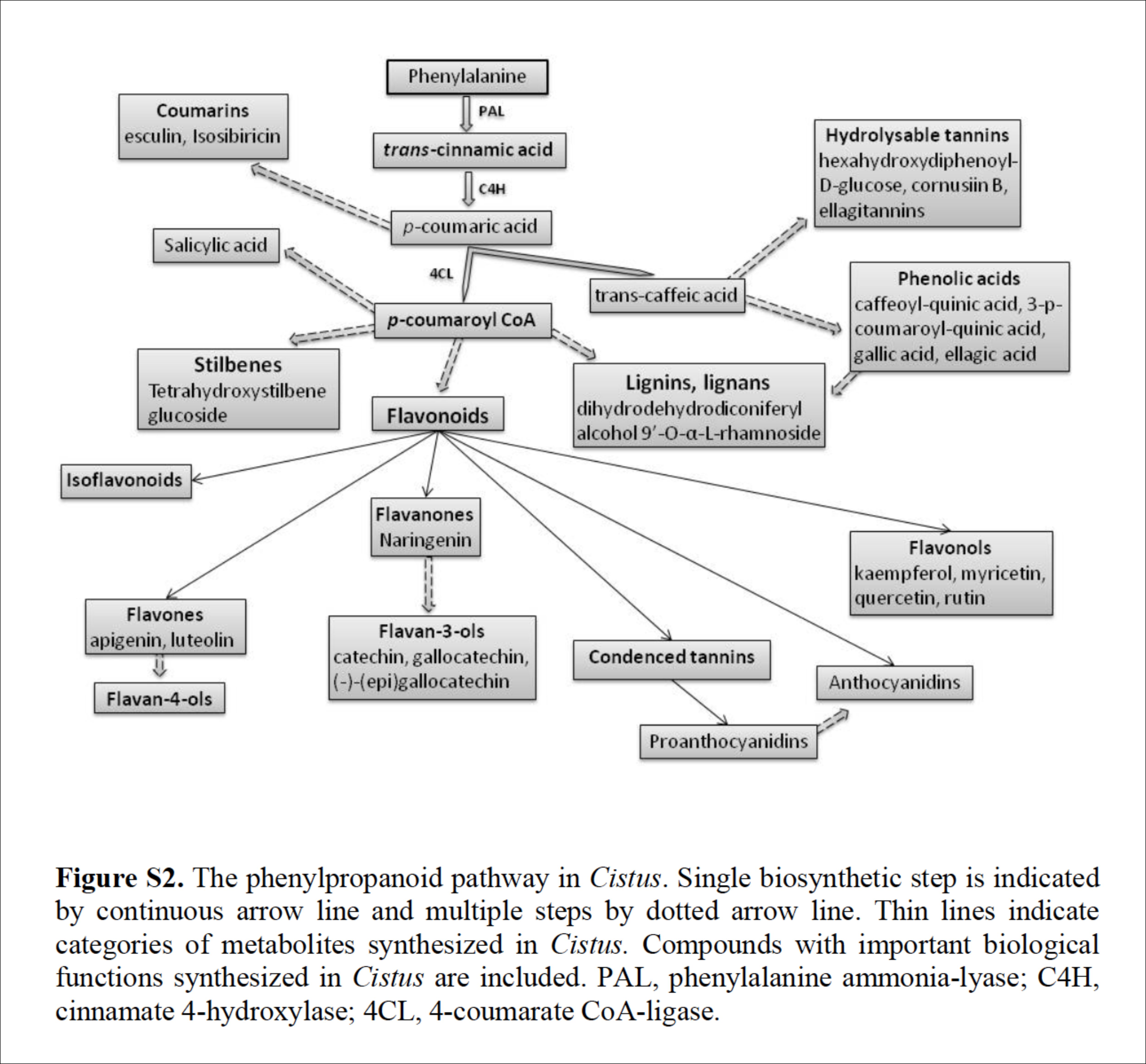

Supplement: Supplementary file 1 [file DataSheet1.ZIP › Supp Fig 2.JPEG]
